# Evidence for low nanocompaction of heterochromatin in living embryonic stem cells

**DOI:** 10.15252/embj.2021110286

**Published:** 2023-04-21

**Authors:** Claire Dupont, Dhanvantri Chahar, Antonio Trullo, Thierry Gostan, Caroline Surcis, Charlotte Grimaud, Daniel Fisher, Robert Feil, David Llères

**Affiliations:** ^1^ Institute of Molecular Genetics of Montpellier (IGMM), CNRS University of Montpellier Montpellier France; ^2^ Institute of Human Genetics (IGH), CNRS University of Montpellier Montpellier France; ^3^ Present address: Montpellier Ressources Imagerie, BioCampus, CNRS, INSERM University of Montpellier Montpellier France

**Keywords:** chromatin organisation, embryonic stem cells, FLIM‐FRET, heterochromatin, HP1, Chromatin, Transcription & Genomics

## Abstract

Despite advances in the identification of chromatin regulators and genome interactions, the principles of higher‐order chromatin structure have remained elusive. Here, we applied FLIM‐FRET microscopy to analyse, in living cells, the spatial organisation of nanometre range proximity between nucleosomes, which we called “nanocompaction.” Both in naive embryonic stem cells (ESCs) and in ESC‐derived epiblast‐like cells (EpiLCs), we find that, contrary to expectations, constitutive heterochromatin is much less compacted than bulk chromatin. The opposite was observed in fixed cells. HP1α knockdown increased nanocompaction in living ESCs, but this was overridden by loss of HP1β, indicating the existence of a dynamic HP1‐dependent low compaction state in pluripotent cells. Depletion of H4K20me2/3 abrogated nanocompaction, while increased H4K20me3 levels accompanied the nuclear reorganisation during EpiLCs induction. Finally, the knockout of the nuclear cellular‐proliferation marker Ki‐67 strongly reduced both interphase and mitotic heterochromatin nanocompaction in ESCs. Our data indicate that, contrary to prevailing models, heterochromatin is not highly compacted at the nanoscale but resides in a dynamic low nanocompaction state that depends on H4K20me2/3, the balance between HP1 isoforms, and Ki‐67.

## Graphical abstract

Quantitative FLIM‐FRET imaging of nanometre‐scale distances between nucleosomes shows that higher‐order chromatin organization in living cells is strikingly different than in fixed cells.

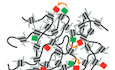

## Introduction

The structural and spatial organisation of chromatin is a major determinant of numerous biological processes, including lineage commitment and cell type‐specific gene expression (Clowney *et al*, 2012; Phillips‐Cremins & Corces, 2013). While most previous studies have addressed how chromatin is organised in fixed cells, the structure and dynamics of chromatin in living cells are becoming accessible through live imaging technologies. Heterochromatin is often thought to be a repressed chromatin state and participates in many functions, including heritable gene repression, genome stability and appropriate chromosome segregation (Allshire & Madhani, 2018). Heterochromatin is defined on the basis of its high DNA density, particularly at chromocenters, which comprise pericentromeric heterochromatin (Maison & Almouzni, 2004; Saksouk *et al*, 2015). Based on many earlier studies, mostly performed on fixed cells, constitutive heterochromatin is commonly assumed to be tightly packaged and inaccessible to certain transcription factors in differentiated cells (Grewal & Jia, 2007; Becker *et al*, 2016), and the DNA within heterochromatin shows high levels of methylation. The formation of constitutive heterochromatin involves histone H3 lysine‐9 trimethylation (H3K9me3), histone H4 lysine‐20 trimethylation (H4K20me3) and the recruitment of chromatin binding proteins such as the chromobox protein HP1α (Heterochromatin protein 1; CBX5), which recognises H3K9me3 and forms bridges between nucleosomes as dimers and oligomers (Hiragami‐Hamada *et al*, 2016; Machida *et al*, 2018).

Mechanistically, it has been proposed that heterochromatin repressive functions arise through the compaction of large chromatin regions (Allshire & Madhani, 2018). Although the mode of such compaction is still debated, several studies have reported that condensed chromatin domains are nevertheless accessible to large macromolecules (Verschure *et al*, 2003). For example, tandem fluorescent EGFP proteins can similarly access euchromatin and heterochromatin (Pack *et al*, 2006). In mouse embryonic stem cells (ESCs), constitutive heterochromatin shows a less repressed structure (Efroni *et al*, 2008), with recent evidence that major satellite repeat (MSR) transcripts can regulate such a permissive and dynamic environment within heterochromatin foci (Novo *et al*, 2022). Heterochromatin organisation has been largely inferred from genomic interaction studies and biochemical and biophysical analysis of chromatin‐associated proteins (Meshorer *et al*, 2006; Bancaud *et al*, 2009; Gaspar‐Maia *et al*, 2009; de Wit *et al*, 2013; Shaban *et al*, 2020). In recent years, nanodomain topology and dynamics have been explored by super‐resolution three‐dimensional fluorescence microscopy combined with DNA fluorescence in situ hybridization (FISH) or by single‐nucleosome tracking (Beliveau *et al*, 2015; Ricci *et al*, 2015; Boettiger *et al*, 2016; Wang *et al*, 2016; Nozaki *et al*, 2017; Szabo *et al*, 2020). These analyses have revealed the presence of discrete heterogeneous domains with irregular shapes driven by nucleosome interactions (Nozaki *et al*, 2017; Kantidze & Razin, 2020; Szabo *et al*, 2020). In parallel to these imaging‐based approaches, chromosome conformation capture (3C)‐based technologies have measured chromatin contact frequencies and revealed the existence of topologically associating domains (TADs), within which functional interactions between genes and distal cis‐regulatory elements occur (Robson *et al*, 2019). Single‐cell Hi‐C and microscopy studies showed a low frequency of TAD interactions and a high degree of heterogeneity between cells (Nagano *et al*, 2013; Cattoni *et al*, 2017). Whereas current microscopic and 3C technologies generate a spatial resolution of ≤ 50 nm and high‐resolution maps for detecting long‐range interactions, these approaches all require crosslinked chromatin and cell fixation processes, are performed on populations of millions of cells or involve the use of probes limited to a small number of genetic loci. Furthermore, studies are often performed on cancer or immortalised cells, where chromatin and particularly heterochromatin undergoes dramatic changes in its organisation and may not reflect its actual state in primary cells (Zink *et al*, 2004; Gurrion *et al*, 2017).

It therefore remains unclear how chromatin is structured at the nucleosomal level in living nontransformed cells and how such nanoscale compaction is regulated. To address this question, we applied a FLIM‐FRET‐based imaging methodology that uses H2B‐GFP and mCherry‐H2B fluorescent proteins to characterise the nanometre‐scale compaction of chromatin in individual living ground‐state ESCs and in differentiating primed cells. Our quantitative approach, which assays nanometre‐scale distances between labelled nucleosomes, reveals infrequent close proximity between nucleosomes within constitutive heterochromatin in naive ESCs and in ESC‐derived epiblast‐like cells (EpiLCs), indicative of a low degree of nanocompaction. We define the term “nanocompaction” as the result of any mechanism that brings labelled nucleosomes in close proximity to each other, meaning in the 1–10 nanometre range.

This low nanocompaction seems unique to pluripotent cells as we did not observe it in differentiated cells. We further investigated the molecular mechanisms that regulate nanocompaction. We found that HP1α plays an important role in the heterochromatin's low compaction state by decreasing the close proximity between nucleosomes, while HP1β, H4K20me2/3 and the cell proliferation marker Ki‐67 are important for maintaining a certain degree of close contacts between them. Our FLIM‐FRET‐based approach provides direct functional insights into the nanocompaction state of chromatin in living pluripotent cells, which contrasts with that in fixed cells most commonly used for chromatin structural studies.

## Results

### A FLIM‐FRET microscopy approach to monitor chromatin nanocompaction in living ESCs

**Figure 1:**
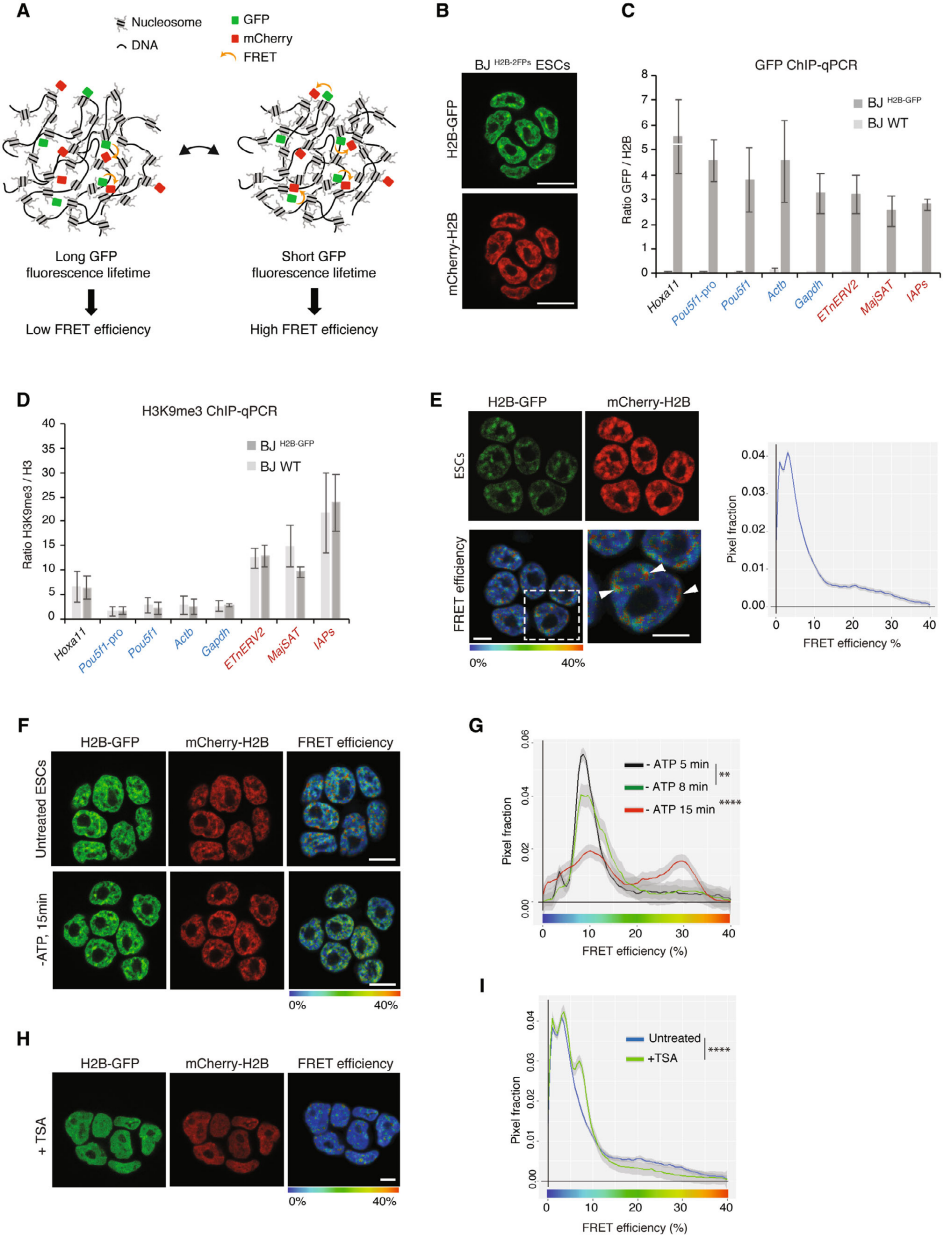
Naive ESCs present different levels of nanocompaction Theoretical representation of the FLIM‐FRET chromatin compaction assay.BJ ESCs stably co‐expressing H2B‐GFP (green) and mCherry‐H2B (red), hereafter named BJ^H2B‐2FPs^.ChIP‐qPCR analysis of GFP enrichment inside different repetitive elements (*ERV*, *MajSat*, *IAPs*) used as H3K9me3 domain controls, or at the *Hoxa11* gene used as a H3K27me3 domain control, or on transcribed genes (*Gapdh*, *Pou5f1*, *Actb*) or actively transcribed gene promoter (*Pou5f1* promoter) in BJ WT and BJ^H2B‐GFP^ ESCs. Locus names are colour‐coded in red whether they are associated with heterochromatic domains (enriched in H3K9me3); in black to Polycomb domains (enriched in H3K27me3); in blue to active chromatin domains (enriched in H3K4me3). Data are represented as relative enrichments of H2B‐GFP versus histone H2B. Data are means ± s.d. (*n* = 3 biological replicates for GFP and histone H2B).ChIP‐qPCR analysis of H3K9me3 enrichment inside different repetitive elements (*ERV*, *MajSat*, *IAPs*) used as H3K9me3 domain controls, or on *Hoxa11* gene used as a H3K27me3 domain control, or on transcribed genes (*Gapdh*, *Pou5f1*, *Actb*) *or* actively transcribed gene promoter (*Pou5f1* promoter) in BJ WT and BJ^H2B‐GFP^ ESCs. Locus names are colour‐coded as in Fig 1C. Data are represented as relative enrichments of H3K9me3 versus histone H3. Data are means ± s.d. (*n* = 3 biological replicates for H3K9me3 and histone H3).Left panel, *in vivo* FLIM‐FRET assay in BJ^H2B‐2FPs^ stably co‐expressing H2B‐GFP (green) and mCherry‐H2B (red). Mean FRET efficiency is displayed using a continuous pseudo‐colour scale from 0 to 40%. Magnification of the FRET map, with discrete high FRET regions indicated by white arrowheads. Scale bars, 5 μm. Right panel, mean distribution of the FRET efficiency related to the pixel fraction from BJ^H2B‐2FPs^ cells (*n* = 384 cells).
*In vivo* FLIM‐FRET measurements in untreated and ATP‐depleted ESCs (‐ATP, 15 min; *n* = 61 cells). Scale bars, 10 μm.Mean distribution of the FRET efficiency from ESCs related to the pixel fraction at different time points of ATP depletion (black, 5 min, *n* = 15 cells; green, 8 min, *n* = 17 cells; red, 15 min, *n* = 61 cells). ****P = 2.2e‐16; K–S test; **P: 0.004107; K–S test.
*In vivo* FLIM‐FRET assay after 9 h of TSA treatment in BJ^H2B‐2FPs^ ESCs. Scale bar, 10 μm.Mean distribution of the FRET efficiency related to the pixel fraction from untreated (blue, *n* = 384 cells) and TSA‐treated cells (green, *n* = 116 cells). ****P = 2.2e‐16; K–S test. Source data are available online for this figure.

**Figure EV1:**
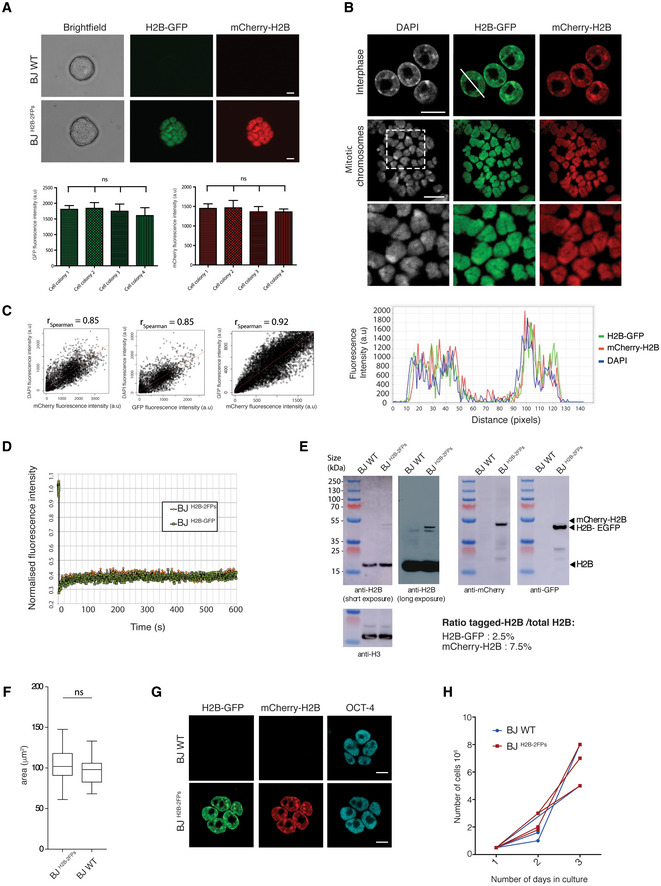
Characterisation of BJ ^H2B‐2FPs^ ESCs suitable to study chromatin nanoscale organisation Colony morphology and co‐expression of fluorophore‐tagged H2Bs in BJ ESCs. Scale bars, 20 μm. The GFP and mCherry fluorescence intensities from different ESC colonies are compared (bottom graphs). The data are presented as means and the error bars represent the standard deviations (*n* = 4 technical independent samples). Each colony contains around  20 cells.DAPI staining on BJ ^H2B‐2FPs^ ESCs in interphase and after mitotic spread. Line scan intensity is indicated by a white line. Scale bars, 10 μm. Fluorescence intensity profiles from this interphase nucleus are displayed below.Spearman correlation between DAPI and mCherry fluorescence intensities (left panel), between DAPI and GFP fluorescence intensities (middle panel) and between GFP and mCherry fluorescence intensities (right panel) obtained from BJ ^H2B‐2FPs^ ESCs (Each correlation, *n* = 65 cells).Quantitative FRAP analysis of H2B‐GFP histones from BJ ^H2B‐2FPs^ and BJ ^H2B‐GFP^ ESCs.Chromatin fractions from BJ^H2B‐2FPs^ ESCs analysed by western blotting with antisera against H2B (short and long exposure), mCherry and GFP. An antiserum against the histone H3 was used as a loading control. The ratios between tagged H2B and total histone H2B are calculated from three western blotting experiments from three biological replicates.Quantification of the nuclei area (μm^2^) of wild‐type BJ ESCs (BJ WT) and BJ ^H2B‐2FPs^ ESCs. The box plots indicate the median values (middle lines), first and third quartiles (box edges) and the whiskers cover the minimum to maximum value range of nucleus area (μm^2^). Data are means of *n* = 3 biological replicates. The number of nuclei analysed is between 50–100. Significance values are computed using Mann–Whitney test, ns = *P* > 0.05.Representative images of immunostaining for POU5F1 (OCT‐4) in BJ WT (top panels) and BJ ^H2B‐2FPs^ ESCs (bottom panels). Scale bars, 10 μm.Proliferation curves of the parental BJ WT (blue, *n* = 3) and BJ ^H2B‐2FPs^ ESCs (red, *n* = 3).

To spatially monitor and quantify close proximity (< 10 nm) between nucleosomes in living cells, we applied Fluorescence Lifetime Imaging Microscopy (FLIM) to measure Förster Resonance Energy Transfer (FRET) between fluorophore‐tagged histones H2B (Fig 1A). This FLIM‐FRET assay was previously applied to cancer cells (Llères *et al*, 2009); here, we adapted it to living ESCs. Low passage naive ESCs (“line BJ”; Sanli *et al*, 2018), derived under serum‐free conditions, were used to integrate vectors expressing H2B‐GFP and mCherry‐H2B. First, a control ESC line expressing only the fluorophore donor H2B‐GFP was derived (hereafter named BJ^H2B‐GFP^), enabling us to assess the fluorescence lifetime values in the absence of the mCherry acceptor proteins. Second, following cell sorting by FACS, this initial cell line was used to derive ESCs that stably co‐expressed tagged H2B‐GFP and mCherry‐H2B (hereafter named BJ^H2B‐2FPs^, Figs 1B and EV1). Fluorophore‐tagged H2B levels were homogenous within and between cell colonies (Figs 1B, and EV1A and B). Line intensity scan profiling and global Spearman correlation coefficient analysis revealed that both H2B‐GFP and mCherry‐H2B co‐localised with DAPI‐stained DNA throughout the nucleus (Spearman coefficient = 0.85), both in interphase and mitotic cells (Fig EV1B and C). To assess the distribution of fluorescent‐tagged H2B histones in different genomic contexts, we performed ChIP‐qPCR experiments against GFP on parental BJ WT and BJ^H2B‐GFP^ ESCs. This revealed that fluorophore‐tagged H2B histones show similar distributions in repetitive sequences characteristic of heterochromatin ‐including intracisternal A particle sequences (IAPs), ETn (Early Transposon) elements (ETnERV2) and pericentromeric major satellite DNA repetitive sequences (Bulut‐Karslioglu *et al*, 2014)‐ as in facultative heterochromatin (*Hoxa11*; Sanli *et al*, 2018) and actively transcribed genes (*Gapdh*, *Actb*, *Pou5f1* gene body and *Pou5f1 promoter*; Kota *et al*, 2014; Fig 1C). Stable incorporation of the tagged H2B histones into chromatin was confirmed by FRAP experiments (Fig EV1D). The incorporation of fluorophore‐tagged H2B histones did not alter the enrichment for H3K9me3 at heterochromatin regions (Fig 1D) and immunoblotting of chromatin‐enriched protein fractions showed that the H2B fusion proteins migrated at the expected molecular sizes (Fig EV1E). The incorporated H2B‐GFP (FRET donor) represented about 2.5% and the incorporated mCherry‐H2B (FRET acceptor) about 7.5%, of total H2B (Fig EV1E). This implies that around 20% of nucleosomes contained one of the two species of fluorescent‐tagged H2B, while less than 1% of nucleosomes are expected to contain both H2B‐GFP and mCherry‐H2B. This, and the respective position of GFP and mCherry, fused to the histone H2B carboxy and amino termini, respectively, considerably limits the possibility of intra‐nucleosomal FRET (Llères *et al*, 2009).

The moderate levels of incorporation had no apparent effects on the ESCs, which showed unaltered colony morphology and an unaltered size of the nuclei (Fig EV1A and F, respectively). The pluripotency transcription factor POU5F1 (OCT4) was expressed similarly in BJ^H2B‐2FPs^ cells as in the original ES line (Fig EV1G), and also showed a similar growth kinetics (Fig EV1H). Together, these data indicate that the BJ^H2B‐2FPs^ ESCs stably co‐expressed H2B‐GFP and mCherry‐H2B at low levels, with a similar distribution, and showed identical behaviour to the original ESC line.

FRET efficiency images were generated by measuring the decrease in the nanosecond fluorescence lifetime of H2B‐GFP due to proximal interactions with mCherry‐H2B. We named the monitored chromatin organisation “nanocompaction,” since the measured FRET is triggered by nanometre proximity (< 10 nm) of the fluorophore‐tagged nucleosomes.

**Figure EV2:**
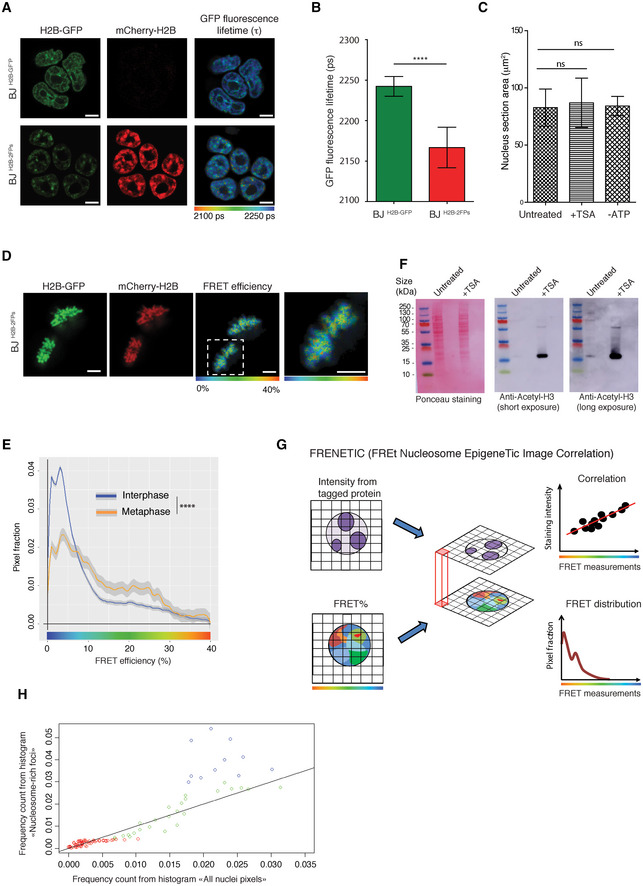
FRET efficiency depends on the proximity between nucleosomes *In vivo* FLIM‐FRET assay in BJ ^H2B‐GFP^ and BJ ^H2B‐2FPs^ ESCs (top and bottom panels, respectively). The mean GFP fluorescence lifetime (τ) is displayed using a continuous pseudo‐colour scale from 2,100 to 2,250 ps. Scale bars, 5 μm.Histogram of the H2B‐GFP fluorescence lifetime from BJ ^H2B‐GFP^ (*n* = 100 cells) and BJ ^H2B‐2FPs^ ESCs (*n* = 101 cells). The data are presented as means and the error bars represent standard deviations. ****P < 0.0001, Mann–Whitney test.Comparison of nuclei area between untreated (*n* = 153 cells), TSA‐treated (*n* = 101 cells) and ATP‐depleted (*n* = 61 cells) BJ ^H2B‐2FPs^ ESCs. The data are presented as means, and the error bars represent standard deviations. ns, *P* = 0.16 for untreated/TSA, *P* = 0.50 for untreated/ATP‐depleted, Mann–Whitney test.
*In vivo* FLIM‐FRET assay on BJ ^H2B‐2FPs^ cells in metaphase. The mean FRET efficiency is displayed using a continuous pseudo‐colour scale from 0 to 40%. Scale bar, 5 μm.Mean distribution of the FRET efficiency (%) related to the pixel fraction from BJ ^H2B‐2FPs^ ESCs at interphase (blue, *n* = 384 cells) and metaphase (orange, *n* = 56 cells). ****, *P* = 2.2e‐16; K–S test.Total cell extracts from untreated and TSA‐treated BJ ^H2B‐2FPs^ ESCs analysed by western blotting with an antiserum against acetyl‐H3 (short and long exposure). Loading control was performed by red ponceau staining.Schematic presentation of the developed FRENETIC (FREt Nucleosome EpigeneTic Image Correlation) workflow to correlate FRET measurements and fluorescence intensity from tagged proteins in living cells.Comparison of the distributions of the “Nucleosome‐rich foci” and “All nuclei pixels” data (see Fig 2E). Blue circles represent data between 0–12% FRET efficiencies, green circles 12–25%, red circles 25–40%. Source data are available online for this figure.

Within BJ^H2B‐2FPs^ ESC colonies, the cells exhibited comparable H2B‐GFP and mCherry‐H2B fluorescence intensities (Fig 1B). H2B‐GFP fluorescence lifetime was measured following two‐photon excitation at 890 nm (see Materials and Methods). A reduced H2B‐GFP mean fluorescence lifetime (217 ± 25 ps) was apparent in BJ^H2B‐2FPs^ ESCs compared with the unquenched lifetime of H2B‐GFP (225 ± 6 ps) in the parental BJ^H2B‐GFP^ ESCs (Fig EV2A and B).

FRET efficiencies were highly heterogeneous throughout the nucleus (Fig 1E, FRET efficiency map). Concordantly, the distribution of the FRET efficiencies (measured on 384 nuclei) suggests a broad range of nanocompaction levels, varying from 0 to 40% (Fig 1E, blue curve). Importantly, the heterogeneity and spatial distribution of nanocompaction were both highly comparable between individual interphase cells (Fig 1E, blue curves). Within the nuclei, FRET efficiencies showed a distinct spatial distribution with the presence of discrete regions of highly‐nanocompacted chromatin (Fig 1E, orange foci highlighted by white arrowheads).

To determine to what extent chromatin nanocompaction in ESCs is dynamic and depends on the metabolic state of the cells, we first assessed the nuclear consequences of ATP depletion. Amongst other effects, ATP depletion leads to an increase in the intracellular pool of divalent cations and polyamines triggering chromatin compaction (Visvanathan *et al*, 2013). Upon chemical ATP depletion with sodium azide (NaN3) and 2‐deoxy‐glucose (2‐DG) in living ESCs, we observed the appearance of multiple bright dense structures comprising both H2B‐GFP and mCherry‐H2B (Appendix Fig S1A and B) and, within 15 min, a marked overall increase in FRET efficiency across interphase nuclei (Fig 1F). A high fraction of the pixels (54 versus 26% in control cells) now showed high FRET between 15 and 40% (green‐orange colour) indicative of high nanocompaction (Fig 1G), without apparent changes in the nuclear section area during the first 15 min of ATP depletion (Fig EV2C). By performing fluorescence recovery after photobleaching (FRAP) experiments of H2B‐GFP at chromocenters in ESCs during ATP depletion (Appendix Fig S1B), we excluded the possibility that the FRET changes monitored were due to changes in the histone H2B dynamics on chromatin at larger spatial (micrometres) and longer temporal (minutes) scales. As previously reported (Maeshima *et al*, 2018), in mitotic cells where ATP levels are naturally decreased, we observed an increase in FRET efficiency levels reflecting the condensation of sister chromatids, with marked differences between chromosomal regions along the metaphase plates (Fig EV2D and E). Because liquid–liquid phase separation (LLPS) has been suggested to be one of the drivers of the global and local dynamics of genome organisation (Shin *et al*, 2018), we explored in living ESCs the effects of 1,6‐hexanediol (1,6‐HD), a widely used tool to disrupt phase‐separated condensates such as heterochromatin foci (Strom *et al*, 2017). It has been reported that high concentrations (5 or 10%) of 1,6‐HD cause chromatin hyper‐condensation and “freeze” chromatin (Itoh *et al*, 2021). We first confirmed that membrane‐less Cajal bodies stained for coilin disappeared upon increasing concentration of 1,6‐HD in ESCs (Appendix Fig S1C). Then, we investigated by FLIM‐FRET whether 1,6‐HD perturbs chromatin nanocompaction in living ESCs treated with 5 or 10% of 1,6‐HD for 5 min (Appendix Fig S1D). In line with previous observations (Itoh *et al*, 2021), we confirmed that nanoscale chromatin compaction was markedly increased upon 5–10% 1,6‐HD treatment (Appendix Fig S1D). Because 5% and 10% of 1,6‐HD drastically affect chromatin motion (Itoh *et al*, 2021), we cannot exclude that besides the effects on nanocompaction, the observed increased FRET efficiency was not in part due to other biophysical parameters such as a reduction in the chromatin mobility and phase separation.

Next, we assessed the role of histone acetylation in chromatin compaction, which weakens the histone tail binding to the DNA and disrupts nucleosome‐nucleosome interactions (Görisch *et al*, 2005; Ricci *et al*, 2015; Otterstrom *et al*, 2019). Thus, we inhibited histone deacetylases (HDACs) with trichostatin‐A (TSA) before performing FRET analysis. TSA treatment increased histone tail acetylation as shown for H3 (Fig EV2F), and significantly reduced FRET efficiencies across the nuclei, again without apparent changes in nuclear area (Figs 1H and EV2C). Changes were especially pronounced in the higher FRET efficiency range after treatment (FRET efficiency > 10%), which indicated decompaction of the most highly compacted chromatin and a concomitant increase in the low nanocompaction states (Fig 1I, shoulder part of the green curve).

Next, we verified whether our FRET measurements provide a *bona fide* readout of the chromatin compaction levels, by comparing it with a technically independent method that assesses chromatin density based on the measurement of the coefficient of variation (CV; Casas‐Delucchi *et al*, 2012). Since in our study H2B‐GFP co‐localises with DAPI‐stained DNA throughout the nucleus, H2B‐GFP signals were used to measure the CV in living cells (Fig EV1C, Spearman coefficient = 0.85). This approach has been extensively used to quantify changes in heterochromatin organisation upon drug treatments affecting histone modifications or genetic modifications (Casas‐Delucchi *et al*, 2012; Grézy *et al*, 2016; Erdel *et al*, 2020; Martin *et al*, 2021; Neguembor *et al*, 2021). We calculated the CV as (σ/μ), where σ represents the standard deviation of the H2B‐GFP intensity values and μ the mean value of H2B‐GFP intensity of individual nuclei. Importantly, as previously reported, our CV measurements show an increased chromatin density upon ATP depletion and, inversely, decompaction in TSA‐treated ESCs (Appendix Fig S1E; Casas‐Delucchi *et al*, 2012). The combined data confirm that compaction levels measured by FRET depend on inter‐nucleosomal proximity and that nanocompaction is regulated by HDAC activity and the metabolic state of the cell.

### Nanocompaction levels do not reflect nucleosome density

**Figure 2:**
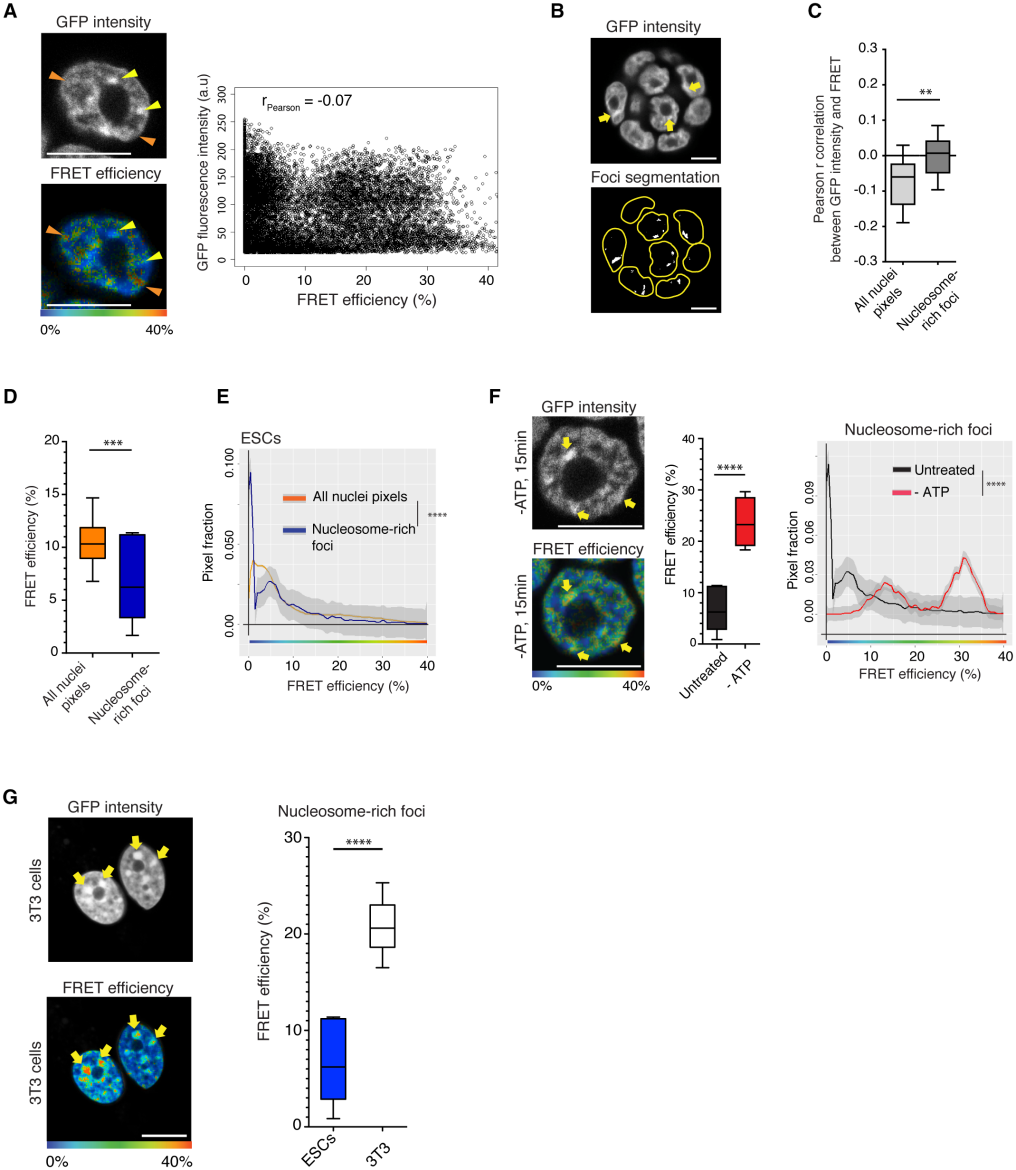
Nucleosome‐rich heterochromatin foci have low levels of nanocompaction in living ESCs, but not in differentiated cells Top and bottom panels depict a representative nucleus of BJ^H2B‐2FPs^ ESC by GFP intensity (nucleosome concentration) and FRET efficiency, respectively. Yellow arrowheads: regions with high nucleosome density but low FRET efficiency; Orange arrowheads: regions with low nucleosome density but high FRET efficiency. Scale bars, 10 μm. The right panel correlates FRET efficiency with GFP fluorescence intensity (Pearson correlation coefficient, *r* = −0.07, *n* = 106 cells).Segmentation of the nucleosome‐rich foci in a representative colony of BJ^H2B‐2FPs^ ESCs by applying the FRENETIC pipeline. Yellow arrows indicate the segmented nucleosome‐rich foci as GFP bright foci. Scale bars, 10 μm.Box‐and‐Whisker plots representing the Pearson correlation coefficients between global GFP intensity with FRET measurements (light grey box), or GFP intensity related to nucleosome‐rich foci (dark grey box). The horizontal lines represent the median, the boxes correspond to the Pearson correlation values from the 25–75^th^ percentiles of the median, with the whiskers covering the Minimum to Maximum value range. **P < 0.01 (Mann–Whitney test, *n* = 106 cells for each condition).Comparison of the FRET efficiency (%) from all nuclear pixels (orange) and pixels exclusively associated with the GFP‐brightest foci (“nucleosome‐rich foci,” blue). FRET efficiency was represented as Box‐and‐Whisker plots. The horizontal lines represent the median, the boxes correspond to the FRET % values from the 25–75^th^ percentiles of the median, and the whiskers cover the Minimum to Maximum value range. ***P < 0.001 (Mann–Whitney test, *n* = 162 cells).Mean distribution of the FRET efficiency of BJ^H2B‐2FPs^ cells from all nuclear pixels (orange) and pixels exclusively associated with nucleosome‐rich foci (dark blue). ****P = 2.2e‐16; K–S test (each condition, *n* = 162 cells).Top and bottom panels depict a representative nucleus of BJ^H2B‐2FPs^ ESC after 15 min of ATP depletion by GFP intensity (i.e. nucleosome concentration) and FRET efficiency, respectively. Yellow arrows indicate nucleosome‐rich foci associated with high levels of FRET. Scale bars, 10 μm. The FRET efficiencies (%) from nucleosome‐rich foci in untreated and ATP‐depleted cells are depicted as Box‐and‐Whisker plots. The horizontal lines represent the median, the boxes correspond to the FRET efficiency (%) values from the 25–75^th^ percentiles of the median, and the whiskers cover the Minimum to Maximum value range. ****P < 0.0001 (Mann–Whitney test, *n* = 61 cells). Mean distribution of the FRET efficiency of BJ^H2B‐2FPs^ from nucleosome‐rich foci in untreated (black, *n* = 162) and ATP‐depleted cells (red, *n* = 61 cells). ****P = 2.2e‐16; K–S test.Left panel, representative image of *in vivo* FLIM‐FRET measurements from differentiated 3T3 cells co‐expressing H2B‐GFP and mCherry‐H2B. Yellow arrows indicate high FRET efficiencies at H2B‐GFP bright chromocenters. Scale bars, 10 μm. Right panel, comparison of the FRET efficiency (%) from ESCs (blue) and 3T3 cells (white) in the GFP‐brightest foci (nucleosome‐rich foci). FRET efficiency (%) was represented as Box‐and‐Whisker plots. The horizontal lines represent the median, the boxes correspond to the FRET % values from the 25–75^th^ percentiles of the median, and the whiskers cover the Minimum to Maximum value range. ****P < 0.0001 (Mann–Whitney test, *n* = 42 cells). Source data are available online for this figure.

Due to its relatively high nucleosome density, heterochromatin can be visualised as DAPI‐bright foci (Probst & Almouzni, 2008). Concordantly, in ESCs we detect a small number of large bright fluorescent nuclear foci (i.e. H2B‐GFP nucleosome‐rich). However, the FRET efficiencies, indicative of chromatin nanocompaction, did not reflect the local density of histones. Some regions showed high fluorescence intensities (i.e. high nucleosome density) but displayed low FRET efficiency (Fig 2A, yellow arrowheads). Conversely, some high FRET regions had a low density of nucleosomes (Fig 2A, orange arrowheads). Combining data from multiple experiments, we observed no correlation between fluorescence intensity levels (i.e. H2B‐GFP density) and FRET efficiency (Pearson coefficient *r* = −0.07, *n* = 106 cells; Fig 2A). Therefore, although our FLIM‐FRET methodology assays close proximity between nucleosomes across the nucleus, it does not simply reflect nucleosome density, as measured conventionally through fluorescence intensity of H2B‐GFP or through DAPI staining.

### Constitutive heterochromatin shows low nanocompaction in ESCs but not in differentiated cells

Next, we assessed nanocompaction specifically within the nucleosome‐rich heterochromatin foci. For this, we segmented the heterochromatic bright foci based on their fluorescence intensity (of H2B‐GFP/mCherry‐H2B) and performed pixel‐based correlation with consecutive measurement of the FRET efficiencies in the segmented sub‐areas. We named this approach “FRENETIC” (FREt Nucleosome EpigeneTic Image Correlation; Fig EV2G, and Materials and Methods).

Following segmentation of the nucleosome‐rich foci (Fig 2B), there was no positive correlation between H2B‐GFP intensity (“nucleosome density”) and FRET efficiency (*r* = 0.01, *n* = 106 cells; Fig 2C). In the segmented H2B‐GFP‐dense foci comprising heterochromatin, we detected significantly lower FRET efficiencies than for the bulk chromatin (6.5 versus 10.5% average FRET efficiency, Mann–Whitney test *P* < 0.001; Fig 2D). Although within the nucleosome‐rich foci the FRET efficiency distribution was broad, most of the pixels showed lower FRET efficiencies as compared to the bulk chromatin profile (Fig 2E, blue and orange curves, respectively; Kolmogorov–Smirnov test *P* < 0.0001 and Fig EV2H). To exclude that the low nanocompaction levels at nucleosome‐rich foci were not due to the accumulation of DNA damages, we monitored the presence of DNA double‐strand breaks (DSBs) during the FRET acquisitions by transiently expressing GFP tagged to the tumour suppressor p53‐binding protein 1 (GFP‐53BP1) in ESCs. It has been shown that in yeast and mammalian cells, DSBs are formed following irradiation with 254 nm UV‐C, as part of the excision repair of pyrimidine dimers (Bradley & Taylor, 1981). Immunostainings for γH2AX in fixed 3T3 cells following UV‐C irradiation confirmed that the construct was functional and that GFP‐53BP1 localised to newly formed DSBs foci within 30 min (Appendix Fig S2A). Next, we performed FLIM‐FRET acquisitions in live ESCs (as well as 3T3 cells) transfected with EGFP‐53BP1 and found no appearance nor accumulation of DSB foci during the process of FRET imaging (Appendix Fig S2B and C).

Importantly, we observed that ATP depletion, which led to the rapid clustering of chromatin and formation of dense nucleosome‐rich foci, caused a strong increase in nanocompaction levels (Fig 2F, yellow arrows and graphs). This result indicates that the low nanocompaction that we measured in nucleosome‐rich heterochromatin foci is not due to the increased chromatin density preventing both fluorescent‐tagged H2B from condensing and interacting with each other to generate FRET. Instead, this is a process that is maintained by active mechanisms that require ATP. Collectively, the above results indicate that nucleosome‐rich regions within ESC nuclei have a relatively low level of nanocompaction.

To investigate whether this is a feature of pluripotent ESCs, we also performed FLIM‐FRET measurements in living 3T3 mouse embryonic fibroblast, which have prominent dense chromocenters (Fig 2G, arrowheads). Within the fibroblast chromocenters, we detected much higher FRET efficiencies as compared to ESCs (Fig 2G, FRET efficiencies map and graph). This demonstrates that the low nanocompaction in dense heterochromatin foci is a feature of pluripotent cells not observed in embryonic differentiated cells.

**Figure 3:**
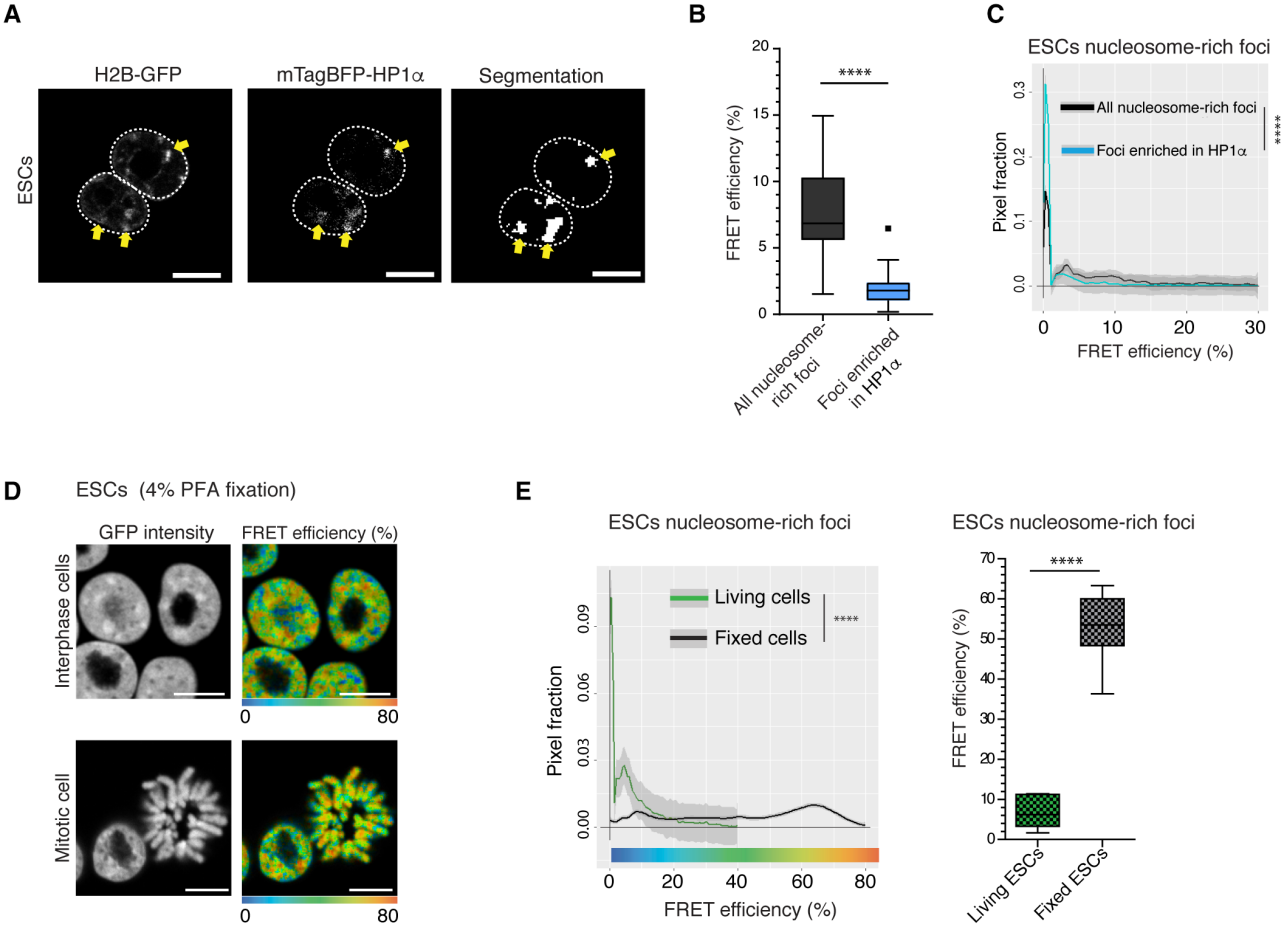
Constitutive heterochromatin shows low nanocompaction levels in living ESCs Expression of mTagBFP‐tagged HP1α in living BJ^H2B‐2FPs^ and segmentation of HP1α positive foci. Yellow arrowheads: chromocenters enriched in H2B‐GFP (left panel), in mTagBFP‐HP1α (middle panel) and following segmentation (right panel). Scale bars, 10 μm.Box plot of the mean FRET efficiencies of all nucleosome‐rich foci (black, *n* = 68 cells) and foci enriched in HP1α (blue, *n* = 68 cells). FRET efficiency (%) represented as Box‐and‐Whisker plots. The horizontal lines represent the median, the boxes correspond to the FRET % values from the 25–75^th^ percentiles of the median, and the whiskers cover the 10–90 percentiles value range. ****P < 0.0001, Mann–Whitney test.Mean distribution of the FRET efficiency of BJ^H2B‐2FPs^ nucleosome‐rich foci associated with HP1α positive foci (blue, *n* = 68 cells) versus all nucleosome‐rich foci (black, *n* = 68 cells). ****P = 2.2e‐16; K–S test.Representative image of the FRET efficiency map of interphase and mitotic ESCs (top and bottom panels, respectively) after fixation with 4% PFA. The mean FRET efficiency is displayed using a continuous pseudo‐colour scale from 0 to 80%. Scale bars, 10 μm.Mean distribution of FRET efficiency from nucleosome‐rich foci in living cells (green, *n* = 162 cells) and fixed cells (black, *n* = 157 cells). ****P = 2.2e‐16; K–S test. Right panel, box plot of the mean FRET efficiency from nucleosome‐rich foci in living and fixed ESCs. FRET efficiency (%) was represented as Box‐and‐Whisker plots. The horizontal lines represent the median, the boxes correspond to the FRET % values from the 25–75^th^ percentiles of the median, and the whiskers cover the Minimum to Maximum value range. ****P < 0.0001, Mann–Whitney test. Source data are available online for this figure.

Chromocenters consist of constitutive heterochromatin and are characterised by the presence of HP1, which binds to the trimethylated form of histone H3 (H3K9me3; Bannister *et al*, 2001). To specifically visualise this HP1‐enriched constitutive heterochromatin, we transiently expressed blue fluorescent protein‐tagged mTagBFP‐HP1α in the BJ^H2B‐2FPs^ cells, in living ESCs (Fig 3A, yellow arrows). mTagBFP‐HP1α localised mostly in the large chromocenters and represented around 32% of all the DAPI‐positive foci, similarly to the endogenous HP1α protein. Next, to quantify the nanocompaction of heterochromatin, we segmented chromocenters based on the mTagBFP‐HP1α marker and analysed their FRET efficiencies using the FRENETIC pipeline (Fig 3A, segmentation panel). FRET efficiencies ranged between 0 and 10%, with an average FRET efficiency of 2% only, which is much lower than the values associated with the chromatin‐dense foci in general (Fig 3B; Mann–Whitney test; *P* < 0.0001). Notably, none of the HP1α‐enriched populations was in the high FRET efficiency range of > 20% (Fig 3C; K–S test; *P* < 0.0001). Combined, the above findings indicate that HP1α‐enriched heterochromatin has a very low level of nanocompaction in living ES cells.

**Figure EV3:**
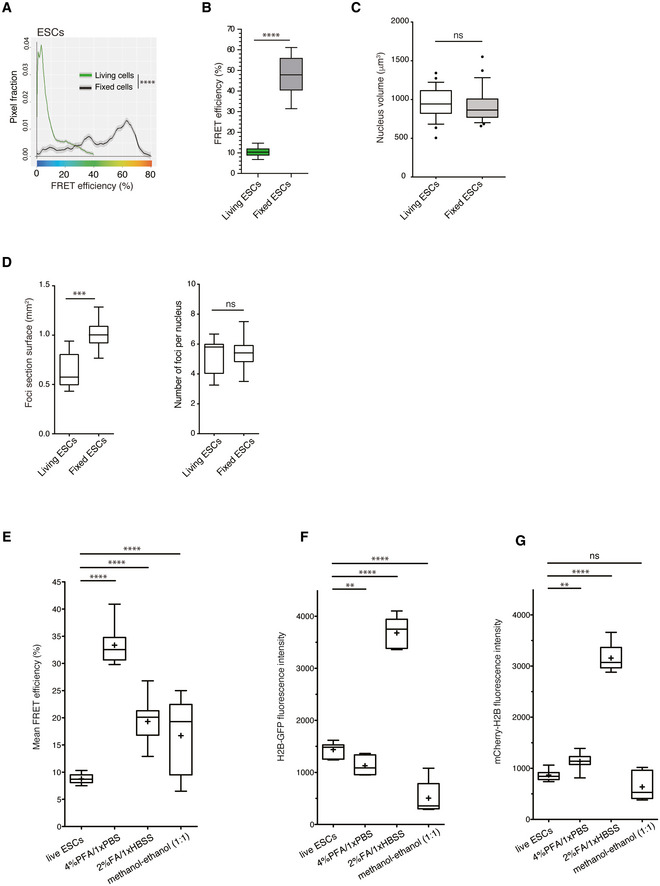
Cell fixation alters the nucleosomal organisation of chromatin Mean distribution of the FRET efficiencies from living ESCs (green, *n* = 384 cells) and fixed cells (black, *n* = 157 cells). ****P = 2.2e‐16; K–S test.Box‐and‐whisker plots of the mean FRET efficiency from living and fixed ESCs. The box plots indicate the median values (middle lines), first and third quartiles (box edges) and the whiskers cover the minimum to maximum value range. Data are means of *n* = 2 biological replicates; *n* = 18 cells for living ESCs and *n* = 19 cells for fixed ESCs. ****P < 0.0001, Mann–Whitney test.Quantification of the nuclear volume of living ESCs and 4% PFA‐fixed ESCs. The Box‐and‐Whisker plots indicate median values (middle lines), first and third quartiles (box edges) and the whiskers cover the 10–90 percentiles value range. Data are means of *n* = 2 biological replicates; *n* = 24 cells for living ESCs and *n* = 26 cells for 4% PFA‐fixed ESCs. Statistical significance was determined by unpaired two‐tailed Student's *t*‐test, ns, *P* = 0.4786.Left panel, Box‐and‐Whisker plots representing the mean section surfaces of foci in living (*n* = 384 cells) and fixed (*n* = 157 cells) ESCs. ***P < 0.001, Mann–Whitney test. Right panel, boxplot of the number of foci per nucleus in living (*n* = 384 cells) and fixed (*n* = 157 cells) ESCs. ns, *P* = 0.40, Mann–Whitney test. The box plots indicate median values (middle lines), first and third quartiles (box edges) and the whiskers cover the minimum to maximum value range.Box‐and‐Whisker plots representing the mean FRET efficiency from living ESCs (*n* = 23) and fixed ESCs using different fixative procedures (4%PFA/1xPBS (*n* = 16); 2%FA/1xHBSS (*n* = 19) and methanol‐ethanol (1:1) (*n* = 9)). The box plots indicate the median values (middle lines), the mean values (middle crosses), first and third quartiles (box edges) and the whiskers cover the minimum to maximum value range. ****P < 0.0001, unpaired two‐tailed Student's *t*‐test.Quantification of the H2B‐GFP fluorescence intensity for living ESCs (*n* = 26) and fixed ESCs using different fixative procedures (4%PFA/1xPBS (*n* = 16); 2%FA/1xHBSS (*n* = 24) and methanol‐ethanol (1:1) (*n* = 13)). The Box‐and‐Whisker plots indicate median values (middle lines), mean values (middle crosses), first and third quartiles (box edges) and the whiskers cover the 10–90 percentiles value range. **P < 0.01; ****P < 0.0001, unpaired two‐tailed Student's *t*‐test.Quantification of the mCherry‐H2B fluorescence intensity for living ESCs (*n* = 26) and fixed ESCs using different fixative procedures (4%PFA/1xPBS (*n* = 16); 2%FA/1xHBSS (*n* = 24) and methanol‐ethanol (1:1) (*n* = 13)). The Box‐and‐Whisker plots indicate median values (middle lines), mean values (middle crosses), first and third quartiles (box edges) and the whiskers cover the 10–90 percentiles value range. **P < 0.01; ****P < 0.0001; ns, *P* = 0.0624, unpaired two‐tailed Student's *t*‐test.

Although heterochromatic genome domains have been described as more open and decondensed in ESCs (Meshorer *et al*, 2006), constitutive heterochromatin is commonly referred to as tightly packed and inaccessible, in accordance with its nucleosome density and diffusion properties of its components (Bancaud *et al*, 2009; Shaban *et al*, 2020). In living ESCs, the pericentromeric heterochromatin foci were indeed dense in nucleosomal material and proteins such as HP1α (Figs 2A and 3A). However, the proximity between nucleosomes measured by FRET in living ESCs indicated a low nanocompaction level. Cell fixation processes strongly alter the density of heterochromatin foci (Imai *et al*, 2017) and the overall nuclear ultrastructure (Guillot *et al*, 2004). Therefore, we investigated whether the commonly used paraformaldehyde (PFA) cell fixation procedure could be responsible for the apparent discrepancy between our FRET efficiency measurements on living ESCs and the conclusions from many other studies, often performed on fixed cells (Linhoff *et al*, 2015; Ricci *et al*, 2015; Boettiger *et al*, 2016; Ou *et al*, 2017; Szabo *et al*, 2020). We therefore performed FLIM‐FRET on PFA‐fixed BJ^H2B‐2FPs^ cells. Indeed, fixation drastically increased the FRET efficiencies within the bulk interphase chromatin, and within mitotic chromosomes, to values reaching 70% (Figs 3D, and EV3A and B). We quantified the nuclear volume and found that this was similar in living and fixed ESCs (Fig EV3C). Next, we examined the nanocompaction levels specifically within constitutive heterochromatin foci. These regions were no longer weakly nanocompacted as in living conditions (Fig 2B) but, instead, were associated with the highest FRET efficiency values within the distribution profile (Fig 3D). The average FRET efficiency level in nucleosome‐rich foci was five times higher than that in living cells (Fig 3E). This tremendous increase in nanocompaction upon cell fixation was accompanied by an increased size of the heterochromatic foci, while their numbers remained unchanged (Fig EV3D). We extended the FRET experiments using different other chemical fixation procedures based on formaldehyde (2%FA/1XHBSS; Nozaki *et al*, 2017) or organic solvent (methanol:ethanol; Ricci *et al*, 2015) and in all cases, we observed higher FRET efficiencies than in living cells (Fig EV3E). These results are independent of the variation of both H2B‐GFP and mCherry‐H2B fluorescence intensity levels upon fixations (Fig EV3F and G). However, different fixation procedures strongly impair chromatin motion (Itoh *et al*, 2021), suggesting that some of the observed high nanocompaction may therefore come from the suppression of the chromatin mobility effects. Together, the above data show that cell fixation procedures completely alter the nucleosomal environment/organisation and consequently increase the nanocompaction of chromosomes and chromatin. This insight highlights the interest of using live‐cell imaging for exploring the structure of (hetero)chromatin.

### HP1α restricts, and H4K20me2/3 increases, the nanocompaction levels of heterochromatin

**Figure 4:**
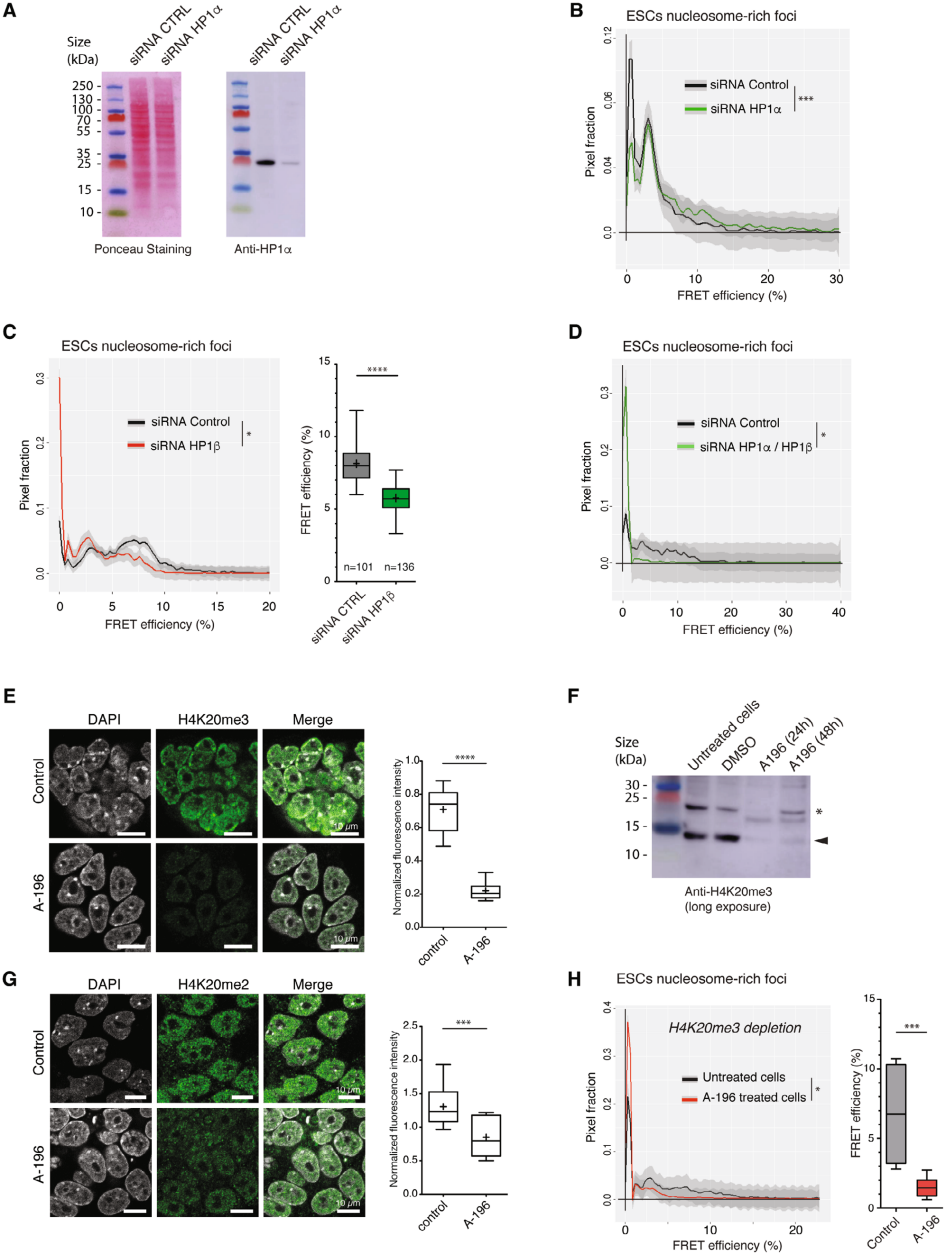
HP1α restricts and H4K20me3 increases, nanocompaction levels within heterochromatin Total cell extracts from BJ^H2B‐2FPs^ cells incubated during 24 h with untargeted siRNA or an siRNA targeting HP1α, analysed by western blotting with an antiserum against HP1α. Loading control was performed by red ponceau staining.Mean distribution of the FRET efficiency from nucleosome‐rich foci of BJ^H2B‐2FPs^ cells incubated with a control siRNA (black, *n* = 72 cells) or an siRNA against HP1α (green, *n* = 96 cells). ***P = 0.0010; K–S test.Left panel, mean distribution of the FRET efficiency from nucleosome‐rich foci of BJ^H2B‐2FPs^ cells incubated with a control siRNA (black, *n* = 101 cells) or an siRNA against HP1β (red, *n* = 136 cells); *P: 0.02651; K–S test. Right panel, box plot of the mean FRET efficiency from nucleosome‐rich foci of living of BJ^H2B‐2FPs^ cells incubated with a control siRNA (grey, *n* = 101 cells) or an siRNA against HP1β (green, *n* = 136 cells). Box‐and‐Whisker plots represent the FRET efficiency (%). The horizontal lines represent the median, the middle crosses represent the mean, the boxes correspond to the FRET % values from the 25–75^th^ percentiles of the median, and the whiskers cover the Minimum to Maximum value range. ****P < 0.0001, Mann–Whitney test.Mean distribution of the FRET efficiency from nucleosome‐rich foci of BJ^H2B‐2FPs^ cells incubated with a control siRNA (black, *n* = 41 cells) or an siRNA against HP1α and HP1β (green, *n* = 100 cells). *P: 0.02651; K–S test.Left panel, representative images of immunostaining for H4K20me3 in control ESCs (top row) and ESCs treated with A‐196 during 2 days (bottom row). Scale bars, 10 μm. Right panel, Box‐and‐Whisker plots represent the normalised fluorescence intensity of immunostaining for H4K20me3. The box plots indicate median values (middle lines), mean values (middle crosses), first and third quartiles (box edges) and the whiskers cover the minimum to maximum value range of the fluorescence intensity. Data are means of *n* = 2 biological replicates. Number of nuclei = 76/60 for control ESCs/A‐196 treated ESCs. Statistical significance was determined by unpaired two‐tailed Student's *t*‐test, ****P < 0.0001.Total cell extracts from untreated, DMSO treated or A‐196 treated BJ^H2B‐2FPs^ during 24 h or 48 h, analysed by western blotting with an antibody against H4K20me3. Arrowhead indicates the H4K20me3‐specific bands. The asterisk indicates unspecific bands.Left panel, representative images of immunostaining for H4K20me2 in control ESCs (top row) and ESCs treated with A‐196 during 2 days (bottom row). Scale bars, 10 μm. Right panel, Box‐and‐Whisker plots represent the normalised fluorescence intensity of immunostaining for H4K20me2. The box plots indicate median values (middle lines), mean values (middle crosses), first and third quartiles (box edges) and the whiskers cover the minimum to maximum value range of the fluorescence intensity. Data are means of *n* = 2 biological replicates. Number of nuclei = 102/111 for control ESCs/A‐196 treated ESCs. Statistical significance was determined by unpaired two‐tailed Student's *t*‐test, ***P = 0.0008.Left panel, mean distribution of the FRET efficiency of untreated BJ^H2B‐2FPs^ cells (black, *n* = 106 cells) and cells treated with A‐196 during 2 days (red, *n* = 115 cells). *P = 0.0231; K–S test. Right panel, Box‐and‐Whisker plots represent the FRET efficiency of nucleosome‐rich foci from untreated BJ ^H2B‐2FPs^ (grey, *n* = 106 cells) and A‐196 treated cells (red, *n* = 115 cells). The box plots indicate median values (middle lines), first and third quartiles (box edges) and the whiskers cover the minimum to maximum value range of the FRET efficiency; ***P < 0.001, Mann–Whitney. Source data are available online for this figure.

**Figure EV4:**
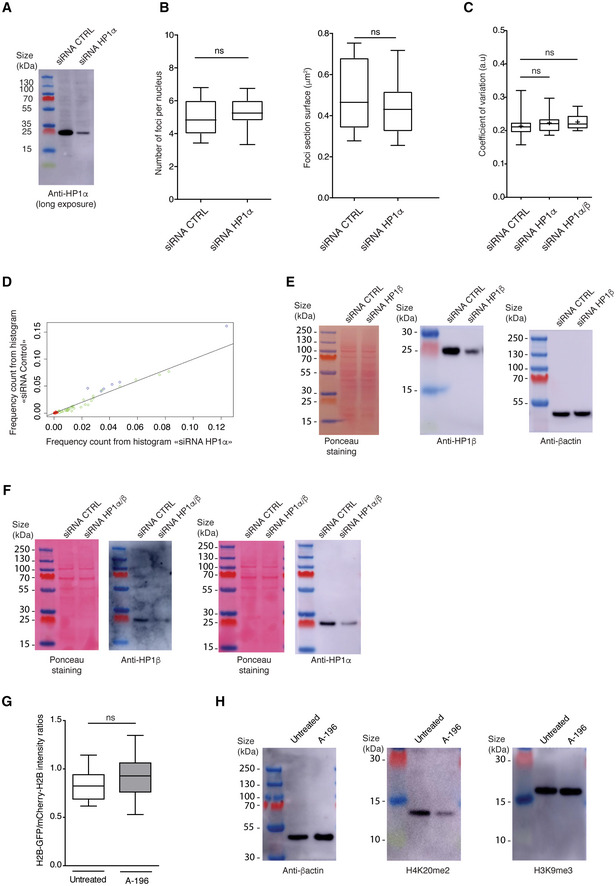
Characterisation of HP1α and HP1β siRNA depletions and A‐196 treatment in BJ ^H2B‐2FPs^ ESCs Total cell extracts from BJ^H2B‐2FPs^ cells incubated during 24 h with untargeted siRNA (siRNA CTRL) and siRNA targeting HP1α, analysed by western blotting with an antiserum against HP1α (long exposure).Left panel, Box‐and‐Whisker plot indicating the number of nuclear foci in BJ^H2B‐2FPs^ treated with siRNA Control (*n* = 180 cells) and siRNA HP1α (*n* = 170 cells). The Box‐and‐Whisker plots indicate the median values (horizontal lines), first and third quartiles (box edges) and the whiskers cover the minimum to maximum value range. ns, *P* = 0.96, Mann–Whitney test. Right panel, boxplot of the mean area of individual focus in BJ^H2B‐2FPs^ treated with siRNA Control (*n* = 180 cells) and siRNA HP1α (*n* = 170 cells). ns, *P* = 0.44, Mann–Whitney test.Compaction of chromocenters was assessed by calculating the coefficient of variation (CV) of the H2B‐GFP signals in ESCs treated with siRNA Control (*n* = 33 cells), siRNA HP1a (*n* = 20 cells; ns, *P* = 0.2415) and siRNA HP1a/b (*n* = 20 cells; ns, *P* = 0.1105). This method has previously been used to measure heterochromatin compaction. The distribution of values is represented by Box‐and‐Whisker plots indicating the median values (horizontal lines), the mean (middle crosses), first and third quartiles (box edges) and the whiskers cover the minimum to maximum value range. Statistical significance was determined by Mann–Whitney test.Comparison of the distributions from “siRNA Control” and “siRNA HP1α” data (see Fig 4B).Total cell extracts from BJ^H2B‐2FPs^ cells incubated during 24 h with untargeted siRNAs (CTRL) and siRNAs targeting HP1β, analysed by western blotting with an antiserum against HP1β (left panels). Loading control was assessed by western blotting with an antiserum against βactin and red ponceau staining.Total cell extracts from BJ^H2B‐2FPs^ cells incubated during 24 h with untargeted siRNAs (CTRL) and siRNAs targeting both HP1α and HP1β, analysed by western blotting with antisera against HP1β (left panels) and HP1α (right panels). Loading control was assessed by red ponceau staining.Box‐and‐Whisker plots of the H2B‐GFP/mCherry‐H2B intensity ratios in untreated ESCs (white box, *n* = 13 cells) and ESCs treated with A‐196 during 2 days (grey box, *n* = 11 cells). The Box‐and‐Whisker plots indicate the median values (horizontal lines), first and third quartiles (box edges) and the whiskers cover the minimum to maximum value range. ns, *P* = 0.06, Mann–Whitney test.Total cell extracts from untreated cells or A‐196 treated BJ^H2B‐2FPs^ cells during 48 h analysed by western blotting with antisera against H4K20me2 (middle panel), or H3K9me3 (right panel). Loading control was assessed by western blotting with an antiserum against βactin (left panel).

HP1α is an essential component of constitutive heterochromatin. Although HP1 proteins association is dynamic in the order of seconds, they are thought to control constitutive heterochromatin assembly and maintain stable heterochromatin subdomains (Bannister *et al*, 2001; Lachner *et al*, 2001; Nakayama *et al*, 2001; Cheutin *et al*, 2003). Recent findings suggest that HP1α proteins can undergo liquid–liquid phase separation (LLPS) *in vitro* to condense DNA (Larson *et al*, 2017). However, another recent study reported that the global chromatin compaction state of chromocenters is independent of HP1 in living differentiated cells (Erdel *et al*, 2020). In this context, it is important to assess, in living ESCs, the role of HP1 proteins in heterochromatin compaction at the nanoscale. SiRNA‐mediated HP1α depletion in BJ^H2B‐2FPs^ ESCs (Figs 4A and EV4A) did not alter the number of H2B‐GFP bright foci, and their surface area also remained constant (Fig EV4B). In nucleosome‐rich foci (chromocenters), measuring the CV in the HP1α siRNA‐treated ESCs, we found that it was similar to that of control siRNA‐treated cells (Fig EV4C). Our results are consistent with prior work showing that heterochromatin density measured by CV is independent of HP1α binding (Erdel *et al*, 2020). Next, we assessed the nanocompaction measured by FLIM‐FRET in the nucleosome‐rich foci (H2B‐GFP bright loci). In the HP1α siRNA‐treated ESCs, the distribution profile of FRET efficiencies was markedly different compared with control siRNA‐treated cells (Fig 4B; K–S test; *P* = 0.0010 and Fig EV4D). In the HP1α‐depleted cells there was a clear reduction in the very low FRET efficiency values range between 0 and 3%, and a moderate but visible increase in the 5–15% FRET efficiency range, indicative of increased nanocompaction levels (Fig 4B). We did not observe any changes in the H2B histone dynamics at chromocenters by FRAP in HP1α siRNA‐treated ESCs (Appendix Fig S3A). These data suggest that the chromatin‐associated HP1α binding or HP1α local concentration in the immediate vicinity of chromatin disturbs the nucleosomal environment and organisation, decreasing nanocompaction.

The HP1 isotype HP1β partly localises to constitutive heterochromatin and broadly participates in the regulation of heterochromatin (Bannister *et al*, 2001; Bosch‐Presegué *et al*, 2017). Upon siRNA‐mediated HP1β depletion in BJ^H2B‐2FPs^ ESCs (Fig EV4E), we observed a significant reduction of the FRET efficiencies in the whole nuclei and particularly within the nucleosome‐rich foci (Fig 4C), while the histone H2B dynamics at chromocenters was unchanged (Appendix Fig S3B). These results suggest that HP1α and HP1β have different roles in the nanoscale organisation of the heterochromatin. Next, we wondered what would be the effect of co‐depleting both HP1α and HP1β on chromatin nanocompaction. In contrast to the HP1α‐single depletion, upon siRNA‐mediated HP1α/β co‐depletion in BJ^H2B‐2FPs^ ESCs (Fig EV4F), the FRET efficiencies within the nucleosome‐rich foci were drastically reduced compared with control siRNA‐treated cells (Fig 4D). Again, as observed for the single HP1 isoform depletions, the rate of exchange of histone H2B was not perturbed upon HP1α/β co‐depletion (Appendix Fig S3C). This finding suggests that HP1β is the major determinant of nanocompaction, which it promotes, and HP1α is important to limit this effect.

A recent study has suggested a direct role for HP1β in the deposition of H4K20me3, which is a hallmark of pericentromeric heterochromatin in differentiated cells (Bierhoff *et al*, 2014; Bosch‐Presegué *et al*, 2017). We found that in ESCs, H4K20me3 displays a punctuate pattern and co‐localises with the nucleosome‐rich foci (Fig 4E). To assess the structural importance of the H4K20me3 enrichment in heterochromatin, we treated the ESCs with a highly‐specific SUV4‐20 H1 and H2 inhibitor, A‐196, which blocks the catalytic SET domain of these KMT (Bromberg *et al*, 2017). This led to a major loss of H4K20me3 and H4K20me2 after 1 day (Fig 4E–G). Importantly, no significant changes were observed in the H2B‐GFP donor/mCherry‐H2B acceptor intensity ratios nor H3K9me3 levels upon A‐196 treatment (Fig EV4G and H). However, at constitutive heterochromatin, the distribution of the FRET efficiencies was significantly altered (Fig 4H, left panel), with a massive reduction in FRET efficiencies towards values below 2% (Fig 4H, right panel) but with no changes in histone H2B dynamics (Appendix Fig S4A). These FRET results were confirmed by applying the CV method to assess changes in DNA compaction (Appendix Fig S4B). These data suggest that H4K20me2/3 contributes strongly to the nanocompaction of constitutive heterochromatin in stem cells. Combined, the above findings indicate that in living ESCs, constitutive heterochromatin presents low levels of nucleosomal compaction. HP1α appears to play a role in this loose organisation by decreasing the proximity between nucleosomes within heterochromatin regions. Conversely, H4K20me2/3 and HP1β are required to maintain nanocompaction levels within heterochromatin.

### Ki‐67 promotes heterochromatin compaction in naive ESCs

**Figure 5:**
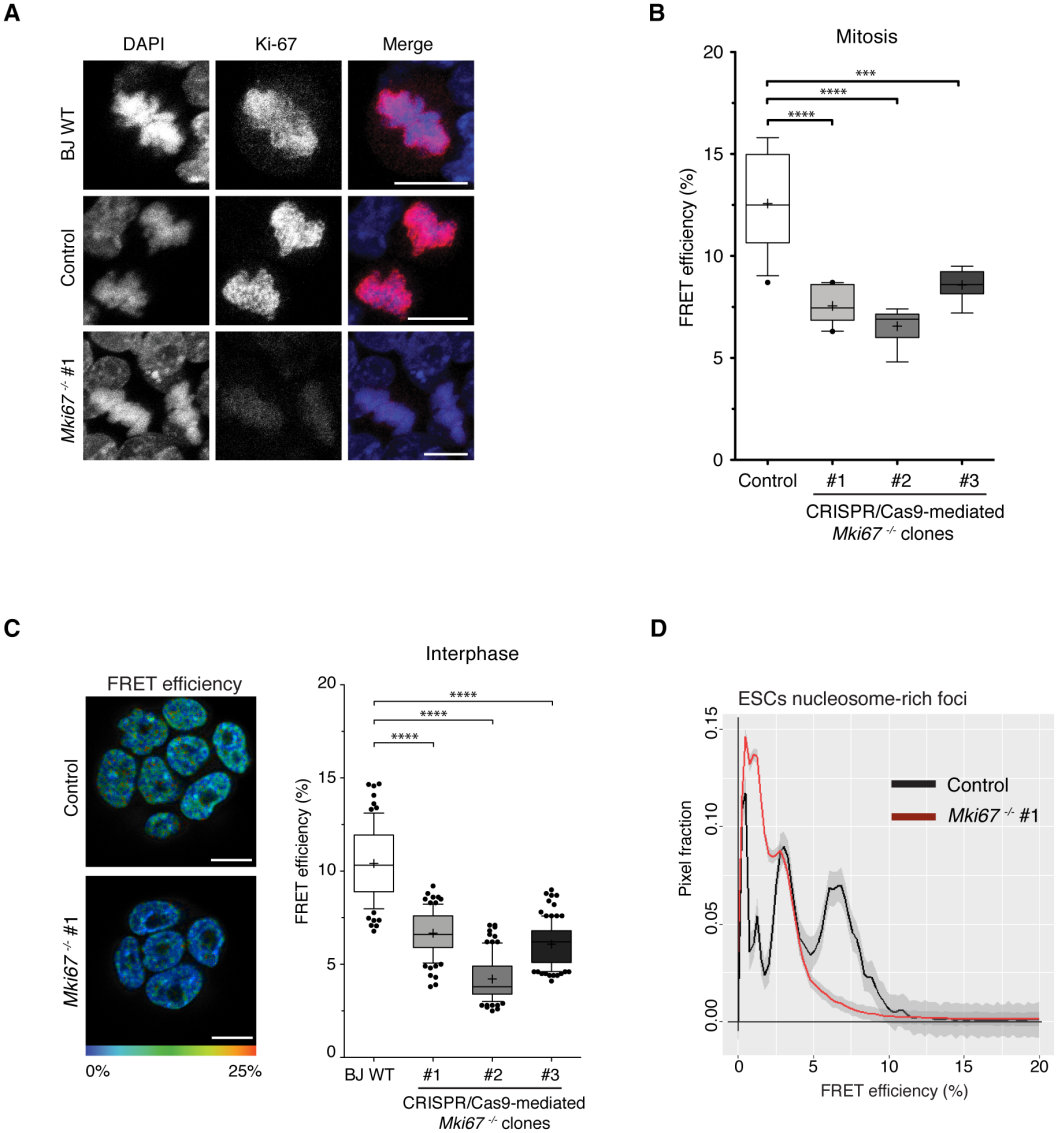
Ki‐67 is dispensable for maintenance of H3K9me3, H4K20me3 and HP1α levels but promotes heterochromatin nanocompaction in naive ESCs Representative images of immunostainings for Ki‐67 in mitotic WT, control (CTRL) and *Mki67* ^−/−^ ESCs. Scale bar, 10 μm.Box‐and‐Whisker plots represent the FRET efficiency (%) from control mitotic ESCs (*n* = 12 cells) and from three different *Mki67* ^−/−^ ESC clones named #1 (*n* = 10 cells), #2 (*n* = 9 cells) and #3 (*n* = 8 cells). The horizontal lines represent the median, the middle crosses represent the mean, the boxes correspond to the FRET % values from the 25–75^th^ percentiles of the median, and the whiskers cover the 10–90 percentiles value range; ***P = 0.0007; ****P < 0.0001, Mann–Whitney test.Left panel, *in vivo* FLIM‐FRET measurements in interphase BJ^H2B‐2FPs^ control ESCs (top) and BJ^H2B‐2FPs^ Mki67^−/−^ ESCs (clone #1). Mean FRET efficiency is displayed using a continuous pseudo‐colour scale from 0 to 25%. Scale bars, 10 μm. Right panel, box‐and‐Whisker plots represent the FRET efficiency (%) in WT ESCs (*n* = 77 cells) and *Mki67* ^−/−^ ESC clones clone #1 (*n* = 86 cells), #2 (*n* = 95 cells) and #3 (*n* = 110 cells). The horizontal lines represent the median, the middle crosses represent the mean, the boxes correspond to the FRET % values from the 25–75^th^ percentiles of the median, and the whiskers cover the 10–90 percentiles value range. ****P < 0.0001, Mann–Whitney test.Mean distribution of the FRET efficiencies in the pixel fraction corresponding to nucleosome‐rich foci from control ESCs (black, *n* = 77 cells) and *Mki67* ^−/−^ ESCs (clone #1, red, *n* = 86 cells). Source data are available online for this figure.

**Figure EV5:**
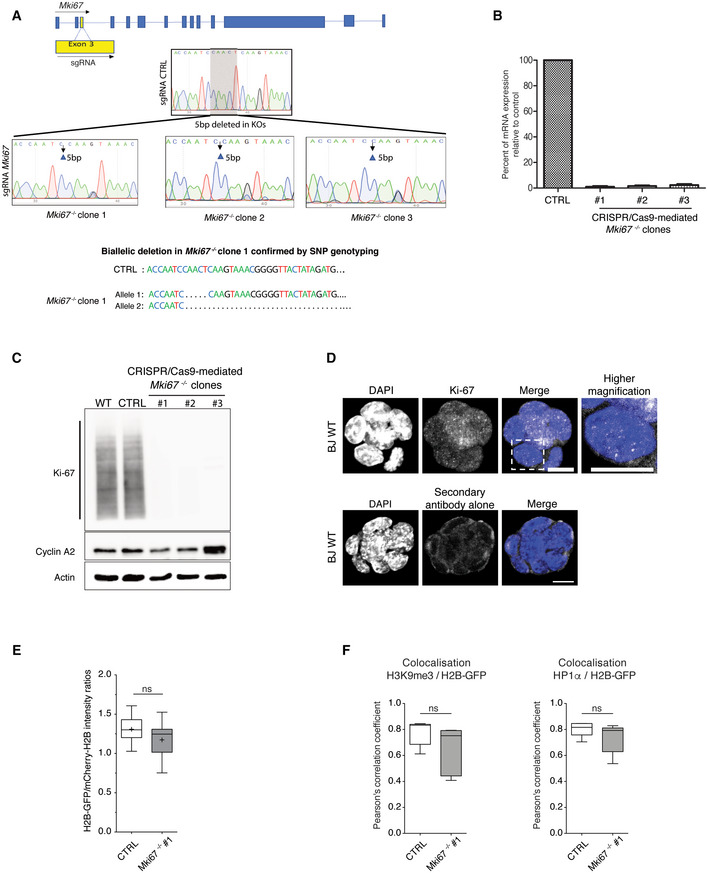
Characterisation of CRISPR/Cas9‐mediated *Mki67* ^−/−^ ES clones Location of the CRISPR‐Cas9‐mediated disruption of the *Mki67* gene in mouse ES cells (targeting exon 3 and resulting in a 5 nt deletion) and sequencing assessment of the deletion in the *Mki67* ^−/−^ clones generated.Expression levels of *Mki67* mRNA in parental control ESCs and in three *Mki67* ^−/−^ ESC clones.Western blotting analysis of the indicated proteins in wild‐type ESCs (WT), control ESCs (CTRL), and *Mki67* ^−/−^ clone #1, clone #2 and clone #3.Top panels, representative images of Immunostaining for Ki‐67 in WT ESCs. Bottom panels, control immunostaining with only the secondary antibody in WT ESCs. Scale bar, 10 μm.Box‐and‐Whisker plot representation of the H2B‐GFP/mCherry‐H2B intensity ratios in control ESCs (white box, *n* = 13 cells) and *Mki67* ^−/−^ clone #1 (grey box, *n* = 15 cells). The Box‐and‐Whisker plots indicate the median values (horizontal lines), the mean values (middle crosses), first and third quartiles (box edges) and the whiskers cover the minimum to maximum value range. ns, *P* = 0.0792, Mann–Whitney test.Left panel, Pearson correlation coefficient represented as a Box‐and‐Whisker plot between H3K9me3 and H2B‐GFP in control ESCs (white box, *n* = 5 ESC colonies) and *Mki67* ^−/−^ clone #1 (grey box, *n* = 4 ESC colonies). The Box‐and‐Whisker plots indicate the median values (horizontal lines), first and third quartiles (box edges) and the whiskers cover the minimum to maximum value range. *P* = 0.2204, unpaired *t*‐test. Right panel, Pearson correlation coefficient represented as a box plot between HP1α and H2B‐GFP in control ESCs (white box, *n* = 6 ESC colonies) and *Mki67* ^−/−^ clone #1 (grey box, *n* = 6 ESC colonies). *P* = 0.2547, unpaired *t*‐test.

Previous studies have shown that the Ki‐67 nuclear cell proliferation antigen accumulates in the nucleolar and heterochromatin regions and interacts with all three mammalian HP1 isoforms, both *in vitro* and *in vivo* (Starborg *et al*, 1996; Scholzen *et al*, 2002). Recently, we reported that Ki‐67 is an important mediator of heterochromatin organisation in proliferating cells (Sobecki *et al*, 2016). To investigate the effects of Ki‐67 on chromatin nanocompaction in naive ESCs, we disrupted the *Mki67* gene in BJ^H2B‐2FPs^ ESCs using CRISPR/Cas9 targeting exon 3 (Figs 5A and EV5A–C).

Given the important role of Ki‐67 in chromosome individualization during mitosis (Booth *et al*, 2014; Cuylen *et al*, 2016), we first verified whether the lack of Ki‐67 affects chromosome compaction in mitotic cells. FLIM‐FRET measurements in three independent *Mki67* ^−/−^ ESC clones indeed revealed a significant decompaction of chromatin in mitotic chromosomes (Fig 5B). Since in interphase ESCs, Ki‐67 has a punctate nuclear localisation throughout nuclei despite its lower expression (Fig EV5D), we also quantified chromatin compaction at this cell cycle stage. First, we controlled that the H2B‐GFP donor/mCherry‐H2B acceptor intensity ratios in *Mki67* ^−/−^ ESCs were similar as in control ESCs (Fig EV5E). FRET measurements showed that in interphase, the *Mki67* ^−/−^ ESCs (three independent clones) had reduced bulk chromatin nanocompaction compared with WT BJ ESCs (Fig 5C). Using our FRENETIC analysis pipeline, we assessed the distribution of the FRET efficiencies at constitutive heterochromatin in the CTRL and *Mki67* ^−/−^ ESCs and found a similar decrease in the absence of Ki‐67 (Fig 5D). In addition, we quantified the colocalisation of both H3K9me3 and HP1α with H2B‐GFP enriched foci and found that both were similar in *Mki67* ^−/−^ and control ESCs (Fig EV5F). Together, these data indicate that Ki‐67 is required to maintain heterochromatin nanocompaction and that its role is likely downstream of HP1 proteins and histone lysine methylation in naive ESCs.

**Figure 6:**
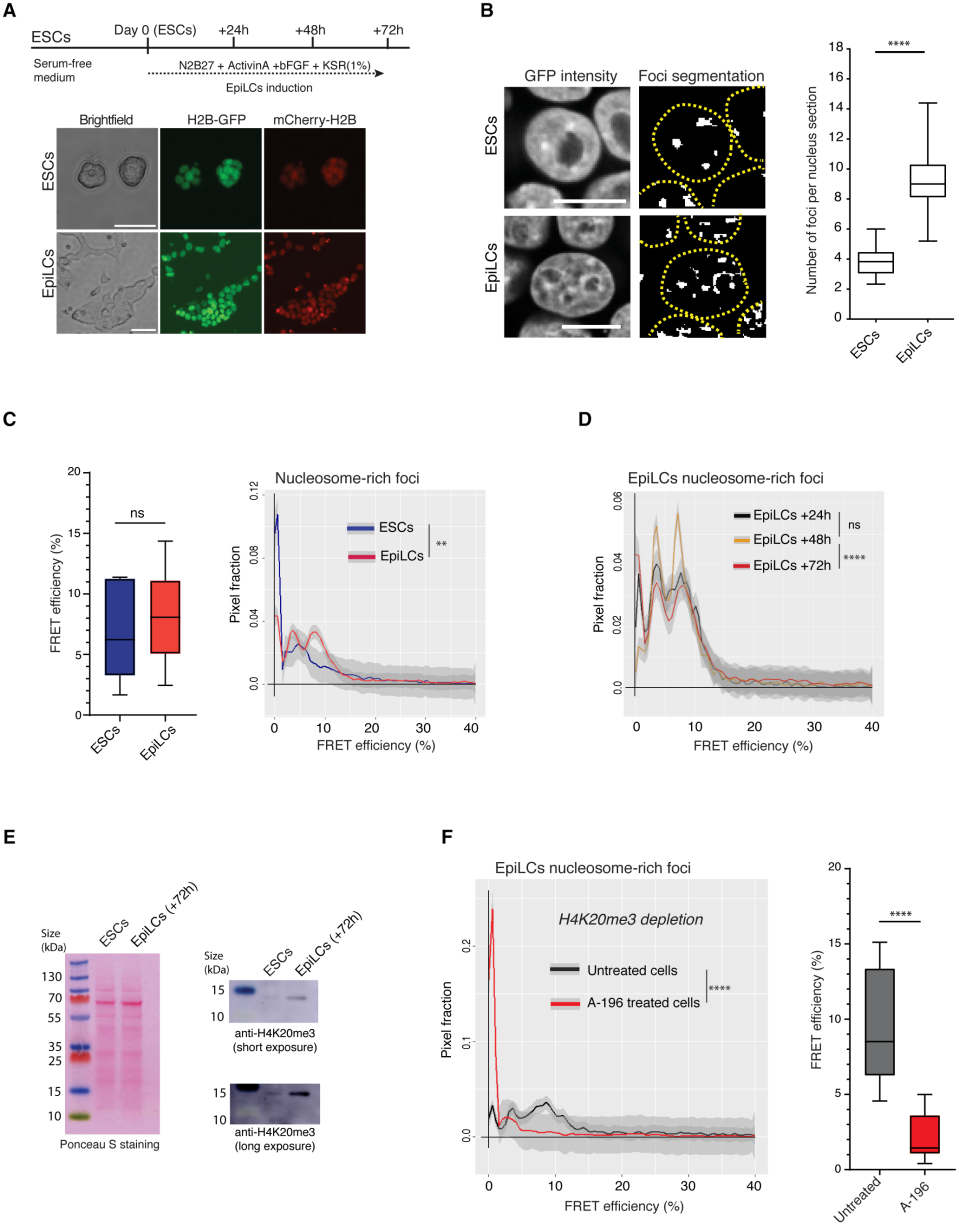
Differentiation into EpiLCs increases the abundance of nanocompacted chromatin that is dependent on H4K20me3 Schematic presentation of the epiblast‐like cell (EpiLC) induction. Colony morphology and co‐expression of tagged H2B in naive ESCs and EpiLCs (line BJ^H2B‐2FPs^). Scale bars, 400 μm.Left panels, nucleosome‐rich foci segmentation in naive ESCs and EpiLCs using the FRENETIC tool. Nuclei are outlined with yellow dashed lines. Scale bars, 10 μm. Right panel, Box‐and‐Whisker plots of the mean number of foci per nucleus in naive BJ^H2B‐2FPs^ ESCs (*n* = 150 cells) and in primed EpiLCs (*n* = 137 cells). The horizontal lines represent the median, the boxes correspond to the number of foci from the 25–75^th^ percentiles of the median, and the whiskers cover the minimum to maximum value range of the number of foci per nucleus section; ****P < 0.0001, Mann–Whitney test.Left panel, Box‐and‐Whisker plots represent the FRET efficiencies (%) from naive ESCs (blue, *n* = 162 cells) and EpiLCs (red, *n* = 266 cells). The box plots indicate median values (middle lines), first and third quartiles (box edges) and the whiskers cover the minimum to maximum value range of the FRET efficiency; ns, *P* = 0.29, Mann–Whitney test. Right panel, mean distribution of the FRET efficiency related to the pixel fraction in nucleosome‐rich foci from naive ESCs (blue, *n* = 162 cells) and EpiLCs (red, *n* = 266 cells). **P < 0.01; K–S test.Mean distribution of the FRET efficiency related to the pixel fraction in nucleosome‐rich foci from EpiLCs at different time points of cellular induction (black, +24 h, *n* = 57 cells; orange, +48 h, *n* = 48 cells; red, +72 h, *n* = 266 cells). ****P = 2.2e‐16; K–S test.Total cell extracts from naive ESCs and EpiLCs (+72 h) analysed by western blotting with an antiserum against H4K20me3. Short and long exposures of the signal are shown. Loading control was achieved by Ponceau staining.Left panel, mean distribution of the FRET efficiency related to the pixel fraction in nucleosome‐rich foci from untreated EpiLCs (black, *n* = 124 cells) and A‐196 EpiLCs treated cells (red, *n* = 113 cells). ****P = 2.2e‐16; K–S test. Right panel, Box‐and‐Whisker plots indicate the FRET efficiency (%) from untreated EpiLCs (black, *n* = 124 cells) and treated with A‐196 during 2 days (red, *n* = 113 cells). The box plots indicate median values (middle lines), first and third quartiles (box edges) and the whiskers cover the minimum to maximum value range of the FRET efficiency. ****P < 0.0001, Mann–Whitney test. Source data are available online for this figure.

### Epiblast‐like cells (EpiLCs) show increased nanocompaction fraction representing heterochromatin

**Figure EV6:**
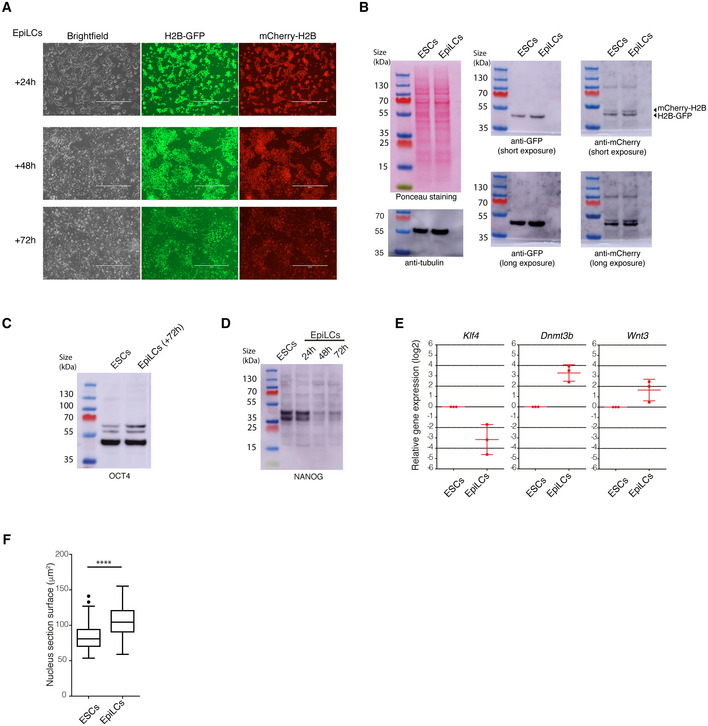
Characterisation of the early differentiation of BJ^H2B‐2FPs^ ESCs into EpiLCs EpiLCs induction from BJ^H2B‐2FPs^ ESCs. Bright‐field and fluorescence images from the H2B‐GFP and mCherry‐H2B reporters are shown. Scale bars, 400 μm.Total cell extracts from naive and EpiLCs BJ^H2B‐2FPs^ (+72 h) analysed by western blotting with antisera against GFP and mCherry. Loading control was assessed by ponceau staining and by western blotting with an antiserum against tubulin.Total cell extracts from naive and EpiLCs BJ^H2B‐2FPs^ (+72 h) analysed by western blotting with an antiserum against POU5F1.Total cell extracts from naive and EpiLCs BJ^H2B‐2FPs^ at different time points after induction analysed by western blotting with an antiserum against NANOG.Gene expression profiles during EpiLCs induction measured by RT‐qPCR. For each gene (*Klf4*, *Dnmt3b* and *Wnt3*), the ΔCT was calculated from two housekeeping genes *Arbp* and *Ppia*. The values are presented on the log2 scale, with ESC values were set up at 0. Data are means of *n* = 3 biological replicates and the error bars represent standard deviations.Box‐and‐Whisker plot representation of the nucleus section surface from ESCs (*n* = 46 cells) and EpiLCs (*n* = 27 cells). The Box‐and‐Whisker plots indicate median values (horizontal lines), first and third quartiles (box edges) and the whiskers cover the 10–90 percentiles value range. ****P < 0.0001, Mann–Whitney test.

Pluripotency progresses through a dynamic continuum of cell states from “naive” to “primed,” which are accompanied by transcriptomic and epigenomic changes (Kalkan *et al*, 2017; Chovanec *et al*, 2021; Nagano *et al*, 2022). Although major changes in chromatin organisation occur between these states (Becker *et al*, 2016; Bromberg *et al*, 2017; Nagano *et al*, 2022), whether heterochromatin structure is altered is unknown. To explore the impact of early differentiation on heterochromatin organisation, we differentiated the naive ESCs into formative pluripotent epiblast‐like cells (EpiLCs; Hayashi *et al*, 2011), which correspond to the epiblast at embryonic stage E6.0, and examined chromatin nanocompaction by FRET. This protocol induced a characteristic morphological change into flattened epithelial cells (Figs 6A and EV6A). Importantly, western blot analyses showed unaltered levels of the fluorophore‐tagged H2B proteins in the obtained EpiLCs (Fig EV6B). As previously reported, after 3 days of EpiLC induction, western blot analyses showed that POU5F1 (OCT4) remained expressed, while NANOG expression was strongly decreased (Fig EV6C and D). During EpiLCs differentiation, the naive state marker gene *Klf4* was downregulated, whereas epiblast markers *Dnmt3b* and *Wnt3* were strongly increased (Fig EV6E), as previously reported (Hayashi *et al*, 2011). We also observed a significant increase in nuclear size after EpiLC differentiation (Fig EV6F).

Segmentation analysis based on H2B‐GFP fluorescence intensity revealed that EpiLC induction triggered a reorganisation of nucleoli and perinucleolar heterochromatin, as well as increasing the number and reducing the size of chromocenters (Fig 6B), which are all characteristic features of differentiated cells (Novo *et al*, 2016). To determine chromatin nanocompaction, we performed FRET measurements in EpiLCs. Interestingly, within the nucleosome‐rich regions, no increased nanocompaction towards higher FRET efficiencies (%) was observed at heterochromatin (Fig 6C, left panel). Instead, the data revealed an enrichment in the fraction of pixels associated with the 5–15% heterochromatic FRET efficiency population that pre‐existed in ESCs (Fig 6C, right panel). In addition, we noticed that two different populations of FRET efficiencies seemed to co‐exist, possibly indicative of two distinct classes of nanocompaction or of two different cell populations co‐existing along the first days of EpiLC induction (Fig 6C, right panel). This multi‐modal profile of nanocompaction associated with heterochromatin is established very early, already after 24 h of EpiLC induction (Fig 6D). Furthermore, western blot analysis indicated that H4K20me3 levels were strongly increased in the induced EpiLCs (Fig 6E). Upon chemical inhibition of SUV4‐20H1/H2 enzymes with A‐196, strikingly, an almost complete loss of nanocompaction was detected in the EpiLCs (Fig 6F).

These findings show that upon early differentiation of ESCs into living EpiLCs, a spatial reorganisation of chromatin occurs, which is associated with changes in the overall distribution of FRET efficiencies. We observed an increased number of heterochromatin regions corresponding to an increased fraction of the chromatin being compacted. However, these regions retained their low FRET efficiency levels, suggestive of a limited compaction level controlled largely by SUV4‐20H1/H2 mediated H4K20me2/3.

## Discussion

Using diverse imaging‐based approaches, the assessment of chromatin compaction in cells has heavily relied frequently on the measurement of parameters that reflect indirectly the different states of organisation. For example, in living cells, the diffusion and dynamics of chromatin‐associated proteins or the local molecular crowding have been used to characterise chromatin compaction (Bancaud *et al*, 2009; Martin & Cardoso, 2010). More broadly, in fixed cells, the density of DNA or the size and number of chromatin compartments, such as heterochromatin foci, are routinely used to quantify their state of chromatin compaction (Martin & Cardoso, 2010; Ricci *et al*, 2015; Martin *et al*, 2021). However, a major outstanding challenge has been to measure directly the physical compaction of chromatin at the nucleosomal scale in living cells. By applying a rigorous quantitative FRET imaging approach, our studies on living cells allowed us to directly assay close distances between fluorophore‐labelled nucleosomes, at the nanometre scale (1–10 nm). We achieved a nontoxic, moderate level of incorporation of both fluorophore‐tagged H2B histones into chromatin—resulting in around 20% of labelled nucleosomes—with a homogenous distribution throughout the genome (Table EV3 and Fig 1C). Therefore, it is unlikely that our FRET imaging assay monitors direct interactions between consecutive nucleosomes in the chromatin fibre. Rather, FRET would occur mostly through direct interactions between different sequences that are brought together in 3D close proximity (Fig 1A).

We define “nanocompaction” as the result of any mechanism(s) that mediates close proximities between nucleosomes, within the 1–10 nanometre range where FRET can occur. Indeed, it is plausible that the physical distance between fluorophore‐tagged histones measured by the FRET efficiency is affected by, or could result from, a combination of molecular and biophysical factors that act either on chromatin organisation, on chromatin motion or on its close environment.

It was reported that local nucleosome fluctuations caused by Brownian motion can facilitate chromatin accessibility in living cells, even in condensed regions (Hihara *et al*, 2012; Ide *et al*, 2022; Lakadamyali, 2022). Our experimental data on ESCs show that chromatin nanocompaction is strongly increased by ATP depletion and by 1,6‐hexanediol exposure. Based on recent work on differentiated cells, these particular conditions likely reduce the local nucleosome motion (Gómez‐García *et al*, 2021; Itoh *et al*, 2021; Iida *et al*, 2022). From these observations, it is tempting to consider that chromatin regions with higher nucleosome motion correspond to lower FRET efficiencies and *vice versa*. However, our FLIM‐FRET measurements are performed at the nanosecond scale, while local fluctuations of individual nucleosomes occur in the time frame of milliseconds (50 nm movement /30 ms; Hihara *et al*, 2012). Furthermore, we observed a drastic increase in nanocompaction levels after cell fixation (Figs 3D and E, and EV3E), while it has been reported that local nucleosome mobility is maintained in fixed cells (Hihara *et al*, 2012). Finally, we revealed by FRET a low nanocompaction in nucleosome‐dense heterochromatin foci (Fig 2), while the local chromatin motion was reported to be independent of chromatin density (Iida *et al*, 2022). For these different reasons, it seems unlikely that local fluctuations of nucleosomes explain the chromatin nanocompaction that we measured by FLIM‐FRET. Other features of chromatin at different scales of organisation are likely more important.

In our FLIM‐FRET approach, we find that the nanocompaction of heterochromatin is limited in naive ESCs, and this is also true for formative pluripotent EpiLCs. In ESCs, HP1α plays an essential role in the nucleosomal organisation of heterochromatin, by decreasing the close proximity between nucleosomes, while the HP1β isoform and the histone modification H4K20me2/3 are important for maintaining contacts between nucleosomes.

A unique aspect of our approach is that chromatin compaction is studied in the context of living cells. Previous studies by others have used cell fixation and permeabilisation protocols and reported that the global chromatin structure of ESCs is more open, dispersed and rather homogeneous *in vivo* (Ahmed *et al*, 2010) and that nucleosomes are organised into discrete “clutches” or “chromatin nanodomains” (Ricci *et al*, 2015; Szabo *et al*, 2020). Here, our data provide direct evidence that chromatin is heterogeneously compacted in live cells, with spatially discrete domains that show high levels of compaction at the nanometre scale. This finding implies that, overall, the chromatin in pluripotent cells is not fully “open” but, instead, adopts various nucleosomal structures. Despite the broad range of compaction levels that we detected within the nuclei, the distribution of the chromatin nanocompaction levels was remarkably similar between individual ESCs. This suggests that the chromatin nanocompaction profiles arise from multiple interrelated dynamic nucleosome interactions that are comparable between cells. It is consistent with the behaviour of chromatin as a polymer forming a variety of local conformations identified as fractal structures inside defined supra‐nucleosomal compacted domains (Li *et al*, 2021). Such different conformations of the chromatin polymer would result not only from self‐interactions between monomer units (i.e. histones) that can be affected by covalent modifications but also by constraints exerted by physical factors such as chromatin proteins, molecular crowding and the surrounding nucleoplasmic environment (divalent cations; De Gennes, 1979). In support of this general model, we find that increased acetylation on the histone tails triggers nanoscale chromatin decompaction and, conversely, that H4K20me2/3 is required for maintaining heterochromatin nanocompaction. Our data are also in line with photo‐activated localization microscopy and with single‐nucleosome tracking studies that have suggested the possible formation of melted polymer nucleosome domains (Nozaki *et al*, 2017). In addition, we find that the higher‐order structures detected by the FLIM‐FRET approach are dynamic and regulated by ATP‐dependent active processes or by ATP‐dependent changes in cation concentrations. In future experiments, it should be relevant to investigate to what extent loop extrusion processes controlled by chromatin proteins like CTCF/cohesin complexes and transcription‐dependent remodelling of chromatin may contribute to the process as well.

Another important main finding is that regions that have a higher nucleosome density do not correspond to areas with higher FRET values. Because of the relatively low level of fluorescent‐histone incorporation, our FRET‐based approach mostly measures physical proximity between nucleosomes that are positioned at intermediate‐to‐long genomic distances. The obtained data reflect a nanoscale organisation of the chromatin that is different from the mesoscale organisation measured by DNA density. Notably, our experiments in living cells show that constitutive heterochromatin is poorly compacted at the nanoscale, a finding that contrasts with prevailing models of condensed and inactive chromosomal domains based on DNA‐FISH, ChIP‐seq and super‐resolution imaging microscopies on fixed chromatin (Ricci *et al*, 2015; Boettiger *et al*, 2016; Ou *et al*, 2017; Miron *et al*, 2020; Szabo *et al*, 2020). The low nanocompaction of heterochromatin observed in living cells might arise from the perpetual dynamics of nucleosome‐nucleosome interactions. This biophysical property of heterochromatin would be consistent with the proposition that chromatin is liquid‐like at the nanoscale and solid‐like at the mesoscale (Nozaki *et al*, 2017; Strickfaden *et al*, 2020). Such a model is supported by our findings on fixed cells, where the environment of nucleosomes is steadily formed and stable due to the formation of covalent chemical bonds and the precipitation of proteins. This dense solid‐like state of chromatin after fixation overstates the number of close interactions between nucleosomes and, consequently, high levels of nanocompaction were measured. Our insights could not have been obtained without the preserved context of chromatin in living cells. The detection of aberrant compaction levels because of cell fixation indicates that studies on living cells are preferable for exploring chromatin structure.

Previous studies by others have revealed that HP1 proteins play major roles in heterochromatin assembly, and this might involve the formation of oligomers (Canzio *et al*, 2011) or the formation of liquid‐like phase‐separated compartments with chromatin *in vitro* (Larson *et al*, 2017; Keenen *et al*, 2021). Although HP1α was characterised as a chromatin crosslinker (Strom *et al*, 2021), interestingly, HP1α proteins bind chromatin transiently with an exchange time on the order of seconds (Cheutin *et al*, 2003; Phair *et al*, 2004). At the molecular level, it remains unclear how precisely HP1 regulates nanoscale heterochromatin structure in living cells. Our work in primary cells suggests that HP1α may contribute to the plasticity of nucleosomes, by reducing the inter‐nucleosome proximity within heterochromatin. Mechanistically, we cannot rule out that perturbations of the chromatin stiffness due to the partial loss of HP1α binding (Strom *et al*, 2021) may alter the distance between nucleosomes or the frequency of nucleosome proximities monitored by FRET. This increased nanoscale plasticity of the chromatin could depend on the HP1‐mediated increase in viscosity associated with its phase separation capability as reported in *in vitro* studies (Keenen *et al*, 2021). Our results are consistent with a recent study reporting that in *S. pombe*, HP1 protein Swi6 favours the dynamics and accessibility of histone residues that affect chromatin compaction (Sanulli *et al*, 2019). This notion is further reinforced by recent multi‐scale chromatin modelling simulations that demonstrate that nucleosome plasticity likely favours liquid‐like chromatin organisation and compaction (Farr *et al*, 2021).

The HP1β isoform is enriched at pericentromeric heterochromatin and specific euchromatic regions and participates in the heterochromatin structure (Bannister *et al*, 2001). HP1β is also an effective competitor of HP1α *in vitro* and was suggested to mediate and stabilise condensed chromatin (Hiragami‐Hamada *et al*, 2016; Keenen *et al*, 2021). Because of the observed heterochromatin nanocompaction increases upon HP1α depletion in ESCs, we consider the possibility that a higher occupancy of HP1β dimers at unoccupied HP1α‐binding sites, may favour nucleosome contacts and, consequently increase nanocompaction. Such a model is supported by our other finding that upon combined HP1α/β depletion, heterochromatin becomes highly decompacted. In addition, recent work has suggested that HP1β is functionally linked to H4K20me3 and SUV4‐20H2 (Bosch‐Presegué *et al*, 2017). Consistent with these observations, our FRET analyses revealed a complete loss of nanocompaction of heterochromatin upon H4K20me3 depletion in ESCs. Inversely, an increased fraction of the chromatin shows high nanocompaction in EpiLCs, which correlated with higher overall levels of H4K20me3.

More broadly, our finding that heterochromatin regions ‐which appear to be stable structures and have a high nucleosome density‐ show significant plasticity of nanoscale compaction is consistent with the notion that stable steady states (nuclear bodies, chromatin states) can emerge from dynamic components (Misteli, 2001).

In conclusion, the promiscuity of nucleosome‐nucleosome interactions and also the probability of interactions within the heterochromatin environment can be modulated dynamically by multiple factors. These include H4K20me3, histone acetylation and rigidity of the chromatin polymer via the action of ATP‐dependent complexes and also the dynamic nature of protein‐chromatin interactions as shown for Ki‐67, a nuclear protein that is intrinsically disordered, and might promote liquid–liquid phase separation (Yamazaki *et al*, 2022). In the context of rapidly proliferating pluripotent cells, which express Ki‐67, ESCs and EpiLCs display low levels of heterochromatin nanocompaction. Our data indicate that Ki‐67 is required to sustain a certain level of compaction. It remains to be determined whether it interacts with components of heterochromatin, including HP1α, and whether its depletion affects the abundance of these proteins within heterochromatin and, consequently, its nanoscale organisation. However, our data are consistent with a recent genome‐wide mapping study which found that Ki‐67 binds centromere‐proximal regions marked with H3K9me3 in interphase (preprint: van Schaik *et al*, 2021). Also, the ability of Ki‐67 to adapt to different chromatin environments remains to be explored. Since Ki‐67 is a large multivalent intrinsically disordered protein (Andrés‐Sánchez *et al*, 2022; Yamazaki *et al*, 2022), it could provide a platform for the binding of numerous chromatin‐related factors involved in regulating chromatin organisation, which is consistent with its interactions identified in human osteosarcoma cells (Sobecki *et al*, 2016). Additional work is necessary to unravel the interplay between these components, and how this regulates chromatin compaction at the nanoscale in living pluripotent and differentiated cells.

## Materials and Methods

### Experimental ESC clone derivation and maintenance

ESCs were derived in serum‐free (2i) medium supplemented with LIF, Mek inhibitor (PD0325901, at 1 mM) and Gsk3 inhibitor CHIR99021 (3 mM) and maintained in ESGRO 1i medium (LIF and Gsk3 inhibitor; Millipore). The ESC line BJ is male and was cultured on gelatin‐coated plates and was serially passaged using Accutase (Millipore, SF006) (Sanli *et al*, 2018). The BJ^H2B‐2FPs^ cell line, which co‐expresses histone H2B fused at its carboxy terminus to GFP with a 6 amino acid linker and histone H2B fused at its amino terminus to mCherry fluorescent proteins with a 16 amino acid linker, was generated in a step‐wise manner. 3.0 × 10^6^ growing BJ ESCs were electroporated with 5 μg H2B‐GFP plasmid using Amaxa Nucleofector reagent (Lonza). 48 h after electroporation, cells expressing GFP were sorted by flow cytometry (FACS Aria, Becton Dickinson) and expanded to generate the negative control cell line BJ^H2B‐GFP^. BJ^H2B‐GFP^ cells were then electroporated with 5 μg of mCherry‐H2B plasmid by following the Amaxa Nucleofector reagent guidelines. 48 h after electroporation, positive single cells expressing both GFP and mCherry signals were sorted by flow cytometry. One clone was selected and expanded into a cell line called BJ^H2B‐2FPs^. Cells were regularly subjected to mycoplasma testing. Metaphase spreads were prepared from BJ^H2B‐2FPs^ cells after mitotic shake‐off, swollen in prewarmed 0.56% KCl for 10 min at 37°C and air‐dried on slides by using a cytocentrifuge (Shandon Cytospin; Thermo Fisher Scientific).

### ESC differentiation into EpiLCs

EpiLCs were induced as described previously (Hayashi *et al*, 2011). Briefly, 1.0 × 10^5^ ESCs were plated onto 12‐well plates precoated with human plasma fibronectin (16.7 ng/ml) and grown in N2B27 medium containing activin A (20 ng/ml), bFGF (12 ng/ml) and KSR (1%). The medium was changed every 24 h for 3 days.

### Fibroblast cell culture

NIH3T3 cells were grown and maintained in Dulbecco's Modified Eagle's Medium (DMEM; GlutaMax™ with high glucose concentration (4.5 g/l)), supplemented with 10% FBS and 1% penicillin/streptomycin (1,000 U/ml penicillin and 1,000 μg/ml streptomycin) at 37°C and 5% CO_2_.

### Antisera and plasmids

For western blotting experiments, the antisera used were as follows: anti‐H2B (1:1,000; Cell Signalling, #8135), anti‐GFP (1:1,000; Roche, #11814460001), anti‐RFP (1:1,000; Chromotek, #3F5), anti‐HP1α (1:500; Upstate, #15.19s2), anti‐HP1β (1:1,000; Cell Signalling, #8676S), anti‐G9a (1:1,000; Millipore #07‐551), anti‐H4K20me2 (1:1,000; Cell Signalling, #9759S), anti‐H4K20me3 (1:2,000; Upstate #07‐463), anti‐H3 (1:1,000; Millipore #06‐755), anti‐H3acetyl (1:1,000; Upstate #07‐352), anti‐Pou5f1 (1:1,000; Abcam, #ab19857), anti‐Nanog (1:1,000; Abcam, #ab80892), anti‐Ki‐67 (1:1,000; Abcam #ab15580), anti‐cyclinA2 (1:2,000; Abcam [EPR17351] #ab181591), anti‐actin (1:1,500; Sigma #A2066); For immunostaining experiments, anti‐HP1α (1:500; Euromedex, clone #2HP‐1H5), anti‐Pou5f1 (1:200; Abcam, #ab19857), anti‐H3K9me3 (1:500; Abcam #ab8898), anti‐H4K20me3 (1:300; Active Motif, #91108), anti‐Ki‐67 (1:250; Abcam, clone SP6, #ab16667). The H2B‐GFP expression vector (pBOS‐H2B‐GFP‐N1) contained a blasticidin resistance gene as a selection marker (Kanda *et al*, 1998). The mCherry‐H2B expression vector was generated by cutting out the H2B‐coding sequence from the pBOS–H2B‐GFP vector with the restriction enzymes *Kpn*I and *Bam*HI and sub‐cloning it into the pEF1a‐mCherry‐C1 vector (Clontech, #631972). The mTagBFP‐HP1α was generated by cutting the HP1α‐coding sequence from the pcDNA4T0 HP1α‐HA vector (a generous gift from Dr. A. Vaquero‐Garcia, Spain) with *Hpa*I and *Eco*RI and subcloned into the cloning site of the pTagBFP‐C1 expression vector (Evrogen, #FP171). The GFP‐53BP1 plasmid was constructed by insertion of the 53BP1‐coding sequence into the cloning site of the EBFP‐C1 expression vector using *Kpn*I and *Xho*I restriction enzymes.

### ATP depletion, HDAC inhibition and SUV4‐20 inhibition

ATP depletion was achieved at 10 mM Na Azide (Sigma‐Aldrich) in combination with 50 mM 2‐DG (Sigma‐Aldrich). For HDAC inhibition, trichostatin‐A (TSA; Sigma‐Aldrich) was added to the cells to a final concentration of 200 ng/ml, for 9 h. Cells were grown in the presence of the SUV4‐20H1/H2 inhibitor A‐196 (Sigma‐Aldrich) at a final concentration of 10 μM for the indicated times.

### siRNAs

siRNAs directed against HP1α and HP1β (siRNA‐HP1α and siRNA‐HP1β; 100 nM) were transfected into ESCs using Lipofectamine RNAiMAX according to the manufacturer's protocol (Thermofisher). Transfection medium was changed after 6 h and ESCs were left for 24 or 48 h before performing microscopy. For siRNA experiments we used:

ON‐TARGET plus Mouse *Cbx5* (HP1α; gene 12419) siRNA SMARTpool (Horizon Discovery, ID: L‐040799‐01‐005).

ON‐TARGET plus Mouse *Cbx1* (HP1β; gene 12412) siRNA SMARTpool (Horizon Discovery # L‐060281‐01‐005).

### CRISPR‐Cas9‐mediated deletion of Ki‐67

The sgRNAs targeting mouse *Mki67* exon 3 and nontargeting control sequences were previously designed (Sanjana *et al*, 2014) and cloned into the LentiCRISPRV2 lentiviral vector. Lentiviruses encoding the sgRNA targeting sequences were produced in HEK cells transfected with LentiCRISPRv2, pMD2.G and psPAX2 and used to transduce the BJ1^H2B‐2FPs^ ESCs. Twenty‐four hours post‐transduction, cells were selected using puromycin. Resistant cells were sorted as single cells on 96‐well plates. After 10–12 days of culture, individual colonies were picked and grown in 6‐well plates. and screened for the loss of Ki‐67 through western blotting. Genomic DNA was extracted from the positive clones, and the region around exon 3 was amplified, purified and sequenced. The deletion/addition was confirmed by PCR and DNA sequencing. Knockout clones were also validated by PCR, western blotting and immunofluorescence analysis.

### Western blotting

For total protein extraction, cells were lysed in RIPA buffer (Sigma‐Aldrich; 30 min, on ice), centrifuged at 13,000 *g* (15 min, 4°C) and supernatants were quantified using the BCA protein assay (Thermo Scientific #RC230518). For western blotting, 2 μg of proteins were boiled in 1× Laemmli buffer (Biorad, #161‐0747) for 15 min. Proteins were separated on the NuPAGE™ 12% Bis–Tris gel (#NP0321BOX), for 1.5 h at RT. Samples were transferred onto PVDF membranes (overnight at 4°C). After 1 h of blocking in 5% w/v milk/TBST at RT, membranes were incubated with primary antisera overnight at 4°C. The membranes were washed 3× with TBST and incubated with secondary antibody, donkey anti‐rabbit‐HRP (GE Healthcare, #NA934) or donkey anti‐mouse‐HRP (Biorad, #170‐6516). Signals were visualised using an ECL method (Western Lightning Ultra, Pelkin‐Elmer, #NEL113001EA) and captured with a CCD imager (Ozyme).

### RNA expression analysis

Total RNA from ESCs and EpiLCs was extracted using miRNEasy Kit (Qiagen) and treated with RNase‐free recombinant DNase‐I (Qiagen). Then, cDNA was synthesised using random hexamers and SuperScript III (Invitrogen, #18080‐044) reverse transcriptase. Expression was quantified by RT‐PCR using SYBR Green I master mix (Roche, #04707516001) on a Lightcycler LC480 apparatus. Obtained values were normalised to the geometric mean of two housekeeping genes (*Arbp* and *Ppia*) and relative expression levels were quantified using the delta–delta *C*_t_ (ΔΔ*C*_t_) method. Primer sequences used are listed in Table EV1.

### Chromatin ImmunoPrecipitation: ChIP‐qPCR

ChIP‐qPCR experiments were conducted using a previously described protocol (Barral *et al*, 2022). Two 15 cm dishes with 2 × 10^7^ cells in 20 ml of culture media were used for each ChIP experiment. Cells were crosslinked with 1% Formaldehyde for 10 min at room temperature on an orbital shaker. Formaldehyde was quenched by adding glycine to 75 mM, incubated at RT for 5 min and rinsed twice with PBS. Cells were scrapped and transferred into 15 ml‐Falcon tubes, spin at 300 *g* for 5 min at 4°C, resuspended with 15 ml Buffer A (20 mM HEPES pH = 7,4, 10 mM EDTA, 0.5 mM EGTA, 0.25% Triton X‐100) and incubated at 4°C for 5 min on a rotating wheel. Cells were centrifuged at 300 *g* for 5 min at 4°C then resuspended with 15 ml Buffer B (20 mM HEPES pH = 7.4, 150 mM NaCl, 10 mM EDTA, 0.5 mM EGTA) and incubate at 4°C for 5 min on a rotating wheel. Cells were centrifuged at 300 *g* for 5 min at 4°C and resuspended in 1 ml of Buffer C (20 mM HEPES pH = 7.4, 10 mM EDTA, 0.5 mM EGTA, 0.1% SDS, 1XPIC). After 10 min on ice, cells were transferred into a 15‐ml sonication tube. Sonication was performed with a Bioruptor: 30 s ON – 30 s OFF, 10 cycles, at high power for a total of 20 cycles with a 10 min pause on ice every 10 cycles. Sonicated chromatin was centrifuged at full speed for 10 min at 4°C. To check sonication efficiency, 10 μl of the sample was reverse‐crosslinked by adding 40 μl ChIP elution buffer (10 mM Tris–pH = 8, 300 mM NaCl, 5 mM EDTA, 0.5% SDS). DNA was incubated at 65°C for 1 h to 6 h with 1,000 rpm shaking and 1 μl RNase A (10 mg/ml) was added to the sonicated DNA and incubated at 37°C for 1 h. Finally, 5 μl Proteinase K (20 mg/ml) was added, incubating at 55°C for 2 h. A 1.5% agarose gel was run to check DNA size distribution. On average, DNA fragments were 350 bp in length.

10 μl Protein A Dynabeads and 10 μl Protein G Dynabeads (20 μl in total) per IP were washed twice in 750 μl 1× Incubation buffer (10 mM Tris–pH = 8, 150 mM NaCl, 1 mM EDTA, 0.5 mM EGTA, 0.15% SDS, 1% Triton X‐100, 0.1% BSA, 1XPIC). Beads were resuspended in 25 μl/IP 1× Incubation buffer and incubated O/N at 4°C on a rotating wheel. For ChIP reaction, 20 μg chromatin +2 μg antibody for histones were used. For a 500 μl reaction, we added chromatin (x) + antisera (y) and 390‐(x + y) μl H_2_O + 100 μl 5× incubation buffer (50 mM Tris–pH = 8, 750 mM NaCl, 5 mM EDTA, 2.5 mM EGTA, 0.75% SDS, 5% Triton X‐100) + 5 ml 10% BSA + 5 ml PIC. 1% was saved for input. ChIP reactions were incubated O/N at 4°C on a rotating wheel. The next day, 25 μl washed beads were added per IP, and then incubated at 4°C for 6 h on a rotating wheel. IP reactions were washed with 1 ml wash buffer 1 (10 mM Tris–pH = 8, 150 mM NaCl, 1 mM EDTA, 0.5 mM EGTA, 0.1% SDS, 0.1% DOC, 1% Triton x‐100); with 1 ml wash buffer 2 (10 mM Tris–pH = 8, 500 mM NaCl, 1 mM EDTA, 0.5 mM EGTA, 0.1% SDS, 0.1% DOC, 1% Triton x‐100); with 1 ml wash buffer 3 (10 mM Tris–pH 8, 250 mM LiCl, 1 mM EDTA, 0.5 mM EGTA, 0.5% DOC, 0.5% NP‐40); with 1 ml wash buffer 4 (10 mM Tris–pH = 8, 300 mM NaCl, 5 mM EDTA, 0.5% SDS). IP reactions were resuspended into 100 μl ChIP elution buffer, and then incubated at 65°C for 15 min with 1,000 rpm shaking. The elution step was repeated one time, and then supernatants were pooled to a final volume of 200 μl. 1% Input was resuspended into 200 μl of ChIP elution buffer. The eluted ChIP‐DNA and inputs were incubated at 65°C O/N, 1,000 rpm shaking; then 2 μl RNase A (10 mg/ml) was added and incubated at 37°C for 1 h. 8 μl of Proteinase K (20 mg/ml) were added and incubated at 55°C for 2 h with interval mix, 30 s ON 500 rpm shaking and 8 min OFF. ChIP‐DNA was extracted with phenol/chloroform and ethanol precipitated and resuspended into 100 μl of water. ChIP experiments were performed three times from independent chromatin preparations and quantitative PCR analyses of ChIP DNAs were performed using an SYBR green quantitative PCR kit (Invitrogen, Thermo Fisher Scientific) and a LightCycler 480 II instrument (Roche). The amount of DNA in ChIP samples was extrapolated from standard curve analysis of chromatin DNA before immunoprecipitation (input), and values were represented as the ratio between the percentage of input obtained for each antibody to the ones obtained for histone H2B and H3 as indicated. Primer sequences used are listed in Table EV2.

### Immunofluorescence studies

ESCs (80,000 cells) were plated on gelatin‐coated glass coverslips. 24 h later, cells were fixed for 10 min in 4% paraformaldehyde in 1× PBS at room temperature. After a 10‐min permeabilisation step with 0.5% Triton X‐100 in 1× PBS, cells were blocked with 0.3% Triton X‐100, 1% goat serum, 1% BSA for 30 min and then incubated with primary antisera for 1 h, washed three times with 1× PBS, 5 min at RT and incubated with secondary antisera for 50 min, followed by DAPI (Sigma‐Aldrich). After a final set of washes with 1× PBS, cells were mounted in Vectashield medium (Vector Laboratories). All images were acquired with a laser scanning confocal microscope (LSM780; Zeiss). Imaging was performed at room temperature using a 63× oil immersion NA 1.4 Plan‐Apochromat objective from Zeiss. Zen (black edition) software (Zeiss) was used for image acquisition. Visualisation and analysis of images were done by using OMERO Open Microscopy Environment (OME) and ImageJ tools. For volume measurements, we performed a 3D analysis using the 3D plugin suite in ImageJ.

### DNA compaction measurements by analysis of the coefficient of variation (CV)

The coefficient of variation (CV) of individual nuclei is calculated as CV = $\frac{\sigma }{\mu }$, where σ represents the standard deviation of the H2B‐GFP intensity values and μ represents the mean value of the H2B‐GFP intensity of the nucleus. CV is a method previously used to measure changes in DNA compaction upon drugs treatments or genetic alterations perturbing the chromatin organisation (Casas‐Delucchi *et al*, 2012; Jeanblanc *et al*, 2012; Grézy *et al*, 2016; Erdel *et al*, 2020; Martin *et al*, 2021; Neguembor *et al*, 2021). The highly condensed, bright chromocenters result in a broader intensity distribution and, therefore, in a higher standard deviation than treated cells with decondensed heterochromatin and a more homogenous H2B‐GFP staining.

### Histone H2B mobility measurements by FRAP experiments

FRAP experiments were conducted using a Zeiss LSM 880 confocal microscope equipped with a 63× oil immersion objective. All chromocenter FRAP experiments were conducted with a pixel dwell of 3.29 μs, an image size of 124 × 124 pixels and a pixel size of 0.14 μm. The 488 nm argon laser (100% laser power) and the laser pulse duration were adjusted to photo‐bleach 60% of GFP fluorescence intensity. Time‐lapse sequences of single optical sections for imaging fluorescence were collected every second for 180 s. The displacement of the nucleus during the time of acquisition was corrected using a custom‐made macro in ImageJ. The fluorescence intensities in the bleached and nonbleached nucleus regions, and background before and after laser photobleaching, were extracted using ImageJ software. Analysis of FRAP data was performed by using the web‐based application EasyFrap‐web (Koulouras *et al*, 2018).

### FLIM‐FRET measurements

FLIM measurements were performed at 37°C with a 40× oil immersion lens, NA 1.4 Plan‐Apochromat objective, with an inverted laser scanning multiphoton LSM780 microscope (Zeiss) equipped with an environmental black‐walled chamber. GFP two‐photon excitation was realised at 890 nm by using a tuneable Chameleon Ultra II (tuning range from 680 to 1,080 nm) laser (Coherent) that provided sub‐150‐fs pulses at an 80‐MHz repetition rate. Detection of the emitted photons from excited GFP was achieved through the use of an HPM‐100 module (Hamamatsu R10467‐40 photomultiplier tube). Laser power was adjusted to give a mean photon count rate of about 5 × 10^4^–10^5^ photons per second. The fluorescence lifetime imaging, corresponding to the time elapsed between laser pulses and the fluorescence photons detection, was provided by time‐correlated single‐photon counting (TCSPC) electronics (SPC‐830; Becker & Hickl). Fluorescence lifetime measurements were acquired over 70s, and fluorescence lifetimes were calculated for each individual pixel in the field of view (256 × 256 pixels). The pixel size is x: 260 nm; y: 260 nm. FLIM analyses were performed using SPCImage software (Becker & Hickl). FRET causes a decrease in the fluorescence lifetime of the donor molecules (GFP). In all the FRET measurements performed in this study (drug treatments, siRNAs, fixation procedures, cellular differentiation, etc), first, the mean fluorescence lifetime τD of the donor (H2B‐GFP) expressed in BJ^H2B‐GFP^ in the absence of the acceptor (corresponding to the non‐FRET conditions) was calculated by applying a mono‐exponential decay model to fit the fluorescence lifetime decays. On the contrary, we applied a bi‐exponential fluorescence decay model to fit the experimental decay curves in the FRET conditions where τDA is the mean fluorescence lifetime of the donor (H2B‐GFP) in the presence of the acceptor (mCherry‐H2B) expressed in BJ^H2B‐2FPs^. By fixing the noninteracting proteins' lifetime τD using data from control experiments (BJ^H2B‐GFP^), the value of τDA was estimated. Then, the conversion of fluorescence lifetime into FRET efficiency for each pixel in the images was achieved according to the formula: FRET efficiency = 1 − (τ_DA_/τ_D_) and the obtained spatial FRET efficiencies were depicted using pseudo‐colours at each pixel in a selected region of interest (ROI) using SPCImage software (Llères *et al*, 2007). FRET distributions were extracted from SPCImage and then normalised and graphically represented using Excel and R programming, respectively. Importantly, we considered the volume of one voxel, that is 0.25 × 0.25 × 0.759 μm = 0.047 μm^3^. Then, we approximated the mean volume of ESC nuclei V_nucleus_ = 733 μm^3^, which corresponds to 15,596 voxels/nucleus. Mammalian cell DNA contains around 3 × 10^7^ nucleosomes. This represents approximately 1924 nucleosomes/ voxel. Considering that 19% of total nucleosomes comprise one fluorophore‐tagged H2B (see Table EV3), each voxel contains on average around 385 tagged nucleosomes that potentially could be involved in FRET. This suggests that the fluorescence lifetime values measured during one FLIM acquisition correspond to the average fluorescence lifetime of many FRET events.

### Comparative image analysis

The image analysis pipeline FRENETIC was structured into three parts: segmentation of whole nuclei or sub‐nuclear regions using fluorescence intensity signal, analysis of the FRET efficiency using SPCimage software and then pixel‐based merging of the segmented matrix (intensity) and FRET efficiency matrix. Briefly, whole ESC nuclei were segmented based on H2B‐GFP two‐photons' fluorescence or tagged protein intensity. Nucleosome‐rich foci were segmented with a manual threshold. Finally, for each associated pixel within the nucleus, the correlation coefficient between intensity and FRET values was analysed. The image analysis software and associated graphical user interface developed for this study are available from the corresponding authors upon request.

### Statistical analysis

Statistical comparisons of average FRET efficiencies, nuclei section surfaces, foci numbers and surfaces were performed using the unpaired student *t*‐test and nonparametric Mann–Whitney test in GraphPad Prism with a two‐tailed *P*‐value at 95% confidence interval. Correlations between GFP, mCherry and DAPI intensity but also between GFP intensity and FRET efficiency were performed on R using Spearman's rank correlation coefficient. Statistical comparisons between FRET efficiency distributions were calculated using the Kolmogorov–Smirnov test in *R* programming on raw data.

## Author contributions

**Claire Dupont:** Conceptualization; data curation; software; formal analysis; validation; investigation; visualization; methodology; writing – review and editing. **Dhanvantri Chahar:** Data curation; formal analysis; investigation; visualization; methodology. **Antonio Trullo:** Software; formal analysis; visualization; methodology. **Thierry Gostan:** Data curation; software; formal analysis; visualization; methodology. **Caroline Surcis:** Data curation; investigation; visualization; methodology. **Charlotte Grimaud:** Data curation; formal analysis; investigation; visualization; methodology. **Daniel Fisher:** Conceptualization; funding acquisition; writing – review and editing. **Robert Feil:** Conceptualization; supervision; funding acquisition; project administration; writing – review and editing. **David Llères:** Conceptualization; data curation; formal analysis; supervision; funding acquisition; investigation; visualization; methodology; writing – original draft; project administration; writing – review and editing.

## Disclosure and competing interests statement

The authors declare that they have no conflict of interest.

## Supporting information



Appendix

Expanded View Figures PDF

Table EV1

Table EV2

Table EV3

Source Data for Expanded View

PDF+

Source Data for Figure 1

Source Data for Figure 2

Source Data for Figure 3

Source Data for Figure 4

Source Data for Figure 5

Source Data for Figure 6

## Data Availability

This study includes no data deposited in external repositories.
