# Supplementary material for: Evidence for low nanocompaction of heterochromatin in living embryonic stem cells
Source: EMBO J. 2023 Apr 21;42(12):e110286. doi: 10.15252/embj.2021110286 (PMC10267699; doi:10.15252/embj.2021110286)
Supplement: Supplementary file 1 — Appendix [file EMBJ-42-e110286-s001.pdf]

Appendix

Evidence for Low Nanocompaction of Heterochromatin in Living Embryonic Stem Cells

Authors: Claire Dupont, Dhanvantri Chahar, Antonio Trullo, Thierry Gostan, Caroline Surcis, Charlotte Grimaud, Daniel Fisher, Robert Feil, David Llères

Correspondence to: david.lleres@igmm.cnrs.fr; Robert.feil@igmm.cnrs.fr

Table of contents

Appendix Figure S1: p.2

Appendix Figure S2: p.4

Appendix Figure S3: p.6

Appendix Figure S4: p.8

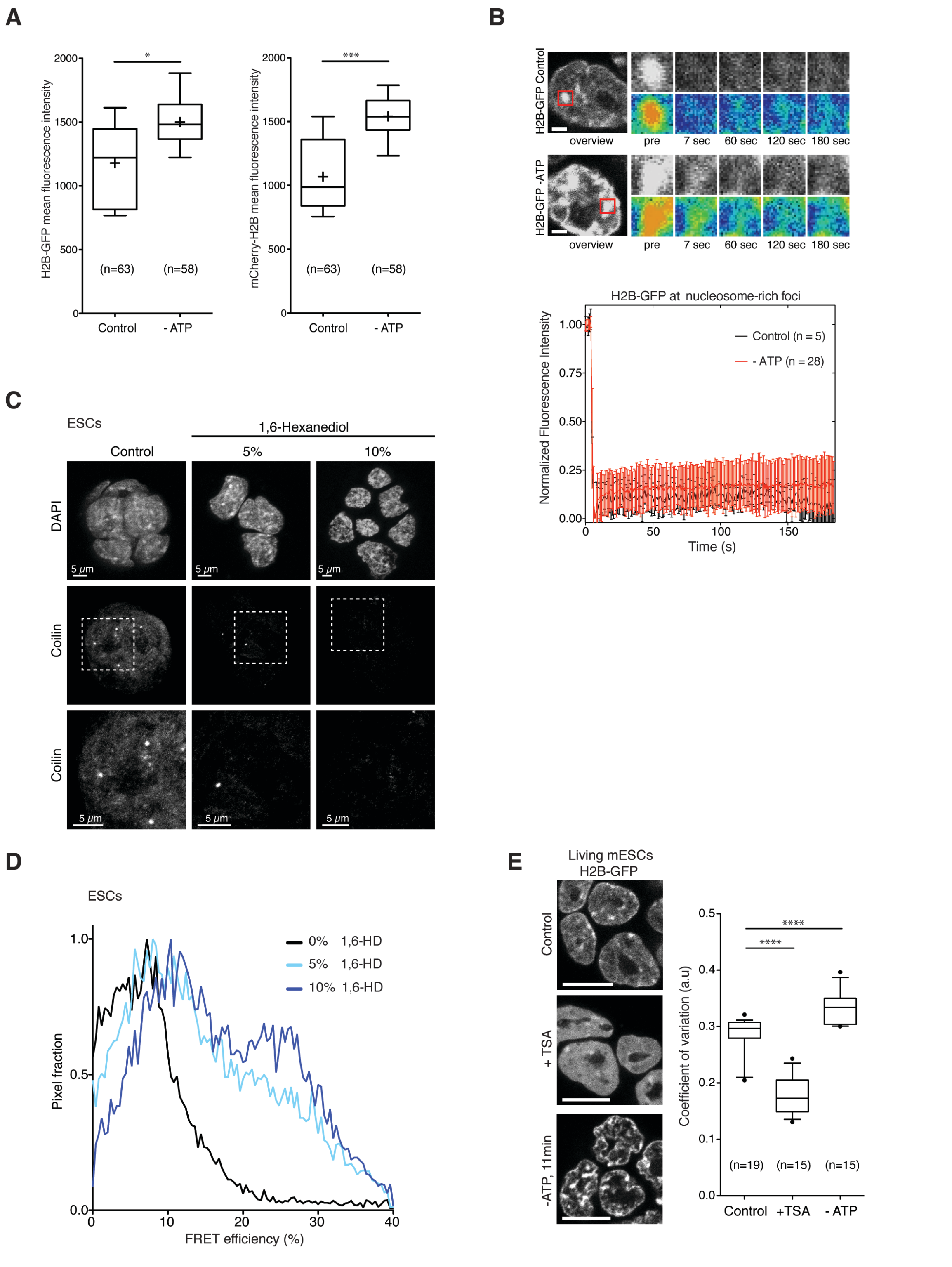

Appendix Figure S1. Characterization of the H2B-GFP levels and dynamics at chromocenters in ESCs following ATP depletion. Effect of 1,6-Hexanediol treatment on chromatin nanocompaction.

A. Left panel, quantification of the H2B-GFP mean fluorescence intensity for living control ESCs and after ATP depletion treatment. n, number of cells analysed. *, p = 0,0156, unpaired t test. Right panel, quantification of the mCherry-H2B fluorescence intensity for living control ESCs and after ATP depletion treatment. n, number of cells analysed. ***, p = 0,0003, unpaired t test.

B. Chromocenter bleach for cells expressing H2B-GFP in control conditions (top panels) or upon ATP depletion (bottom panels). The inset shows the intensity of the pre-bleached and bleached chromocenter during the first 180 s of the experiment. FRAP analysis of H2B-GFP at chromocenters (nucleosome-rich foci) in control (black data points) and ATP depleted ESCs. n, number of chromocenters analysed.

C. Effects of 5% and 10% 1,6-Hexanediol treatments on Cajal bodies immunostained against coilin protein in ESCs. Top row, DNA is stained with DAPI. Middle row, anti-coilin fluorescence images. Third row, high magnification images of the boxed regions define in the middle row.

D. Mean distribution of the FRET efficiency (%) related to the pixel fraction from BJ $^{H2B\text{-}2FPs}$ ES cells (black curve, control cells: 0% 1,6-HD) and after 5 min treatment with 5% 1,6-HD (cyan curve), or 10% 1,6-HD (blue dark curve).

E. The coefficient of variation (CV) is calculated to quantify changes in DNA compaction of individual nuclei in control living mESCs or upon trichostatin (TSA) treatment, or ATP depletion. Representative images of living ESC nuclei in each experimental condition are shown. n, number of cells analysed. ****, p < 0,0001, unpaired t test.

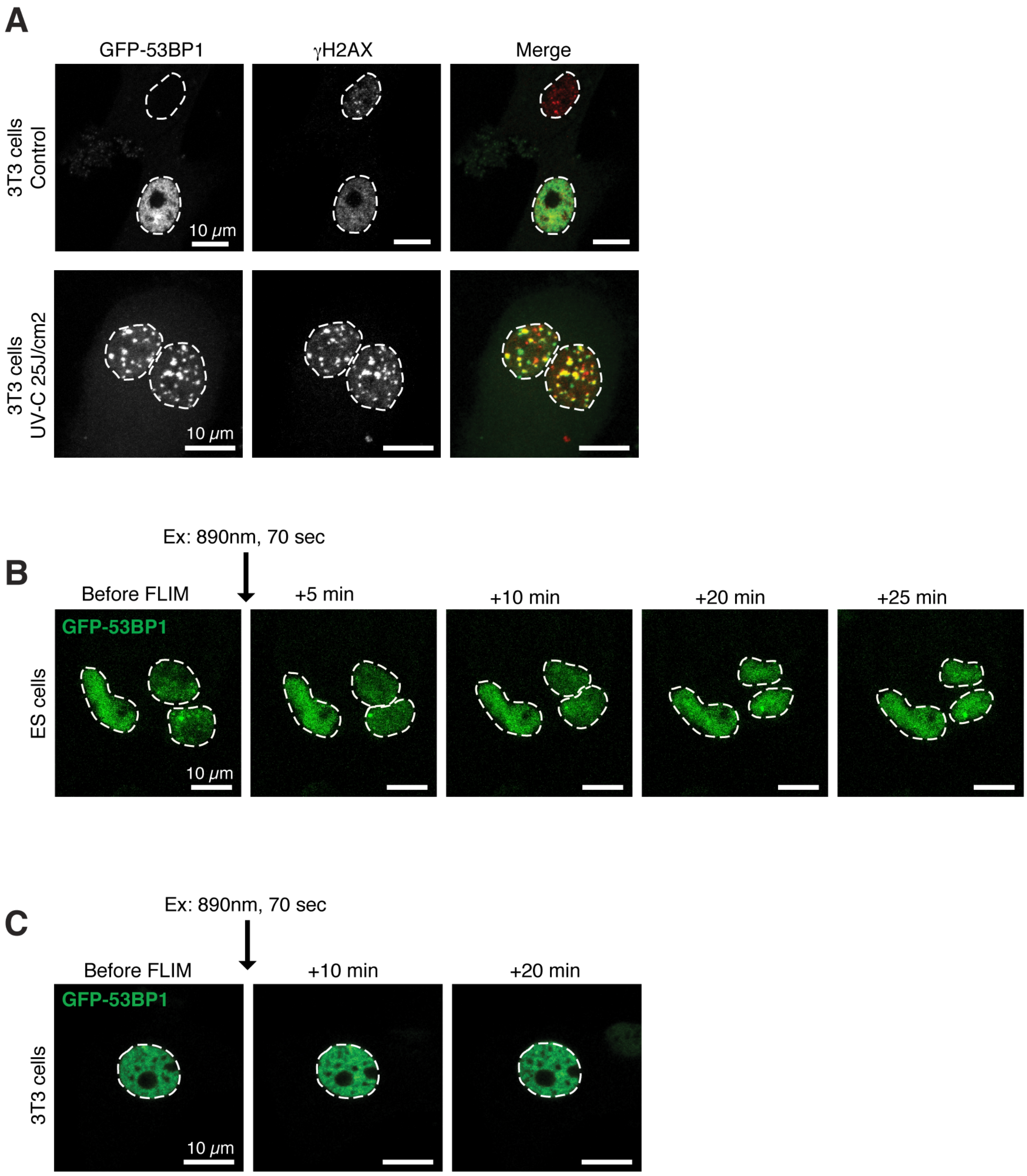

Appendix Figure S2. Absence of DNA damage accumulation during FLIM imaging procedure.

- **A.** 3T3 fibroblast cells transiently expressing GFP-53BP1 protein were exposed to UV-C (25J/cm^2) and immunostained against γH2AX. After UV-C exposure, GFP-53BP1 localise into γH2AX foci. Scale bars, 10 μm.
- **B.** ESCs transiently expressing GFP-53BP1 protein were imaged before and after biphotonic pulsed laser excitation at 890 nm for 70sec. The nuclear localisation of GFP-53BP1 was followed during 25 min post-illumination. Scale bars, 10 μm.

C. 3T3 fibroblast cells transiently expressing GFP-53BP1 protein were imaged before and after biphotonic pulsed laser excitation at 890 nm for 70sec. The nuclear localisation of GFP-53BP1 was followed during 20 min post-excitation. Scale bars, 10 μm.

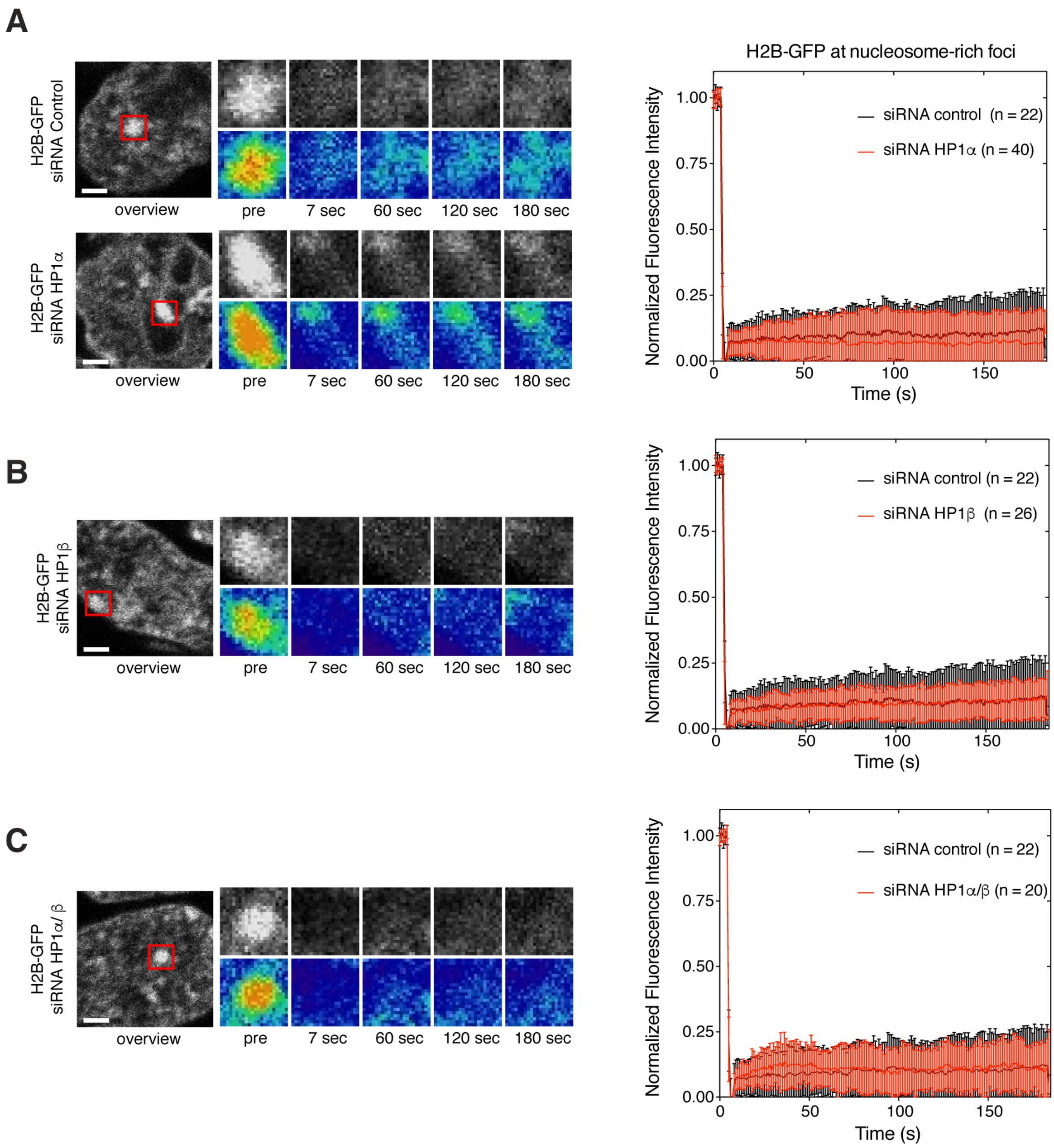

Appendix Figure S3. Characterization of the H2B-GFP dynamics by FRAP at chromocenters in ESCs incubated with siRNA targeting HP1 isoforms.

- **A.** Chromocenter (nucleosome-rich focus) bleach from cells expressing H2B-GFP incubated during 24h with untargeted siRNA (Control, top panels) and siRNA targeting HP1α (bottom panels). The inset shows the intensity of the pre-bleached and bleached chromocenter during the first 180 s of the experiment. FRAP analysis of H2B-GFP at chromocenters (nucleosome-rich foci) in siRNA control (black data points) and siRNA targeting HP1α (red data points). n, number of chromocenters analysed.
- **B.** Same as **A** but after incubation during 24h with siRNA targeting HP1β.

C. Same as **A** but after incubation during 24h with siRNAs targeting both HP1α and HP1β.

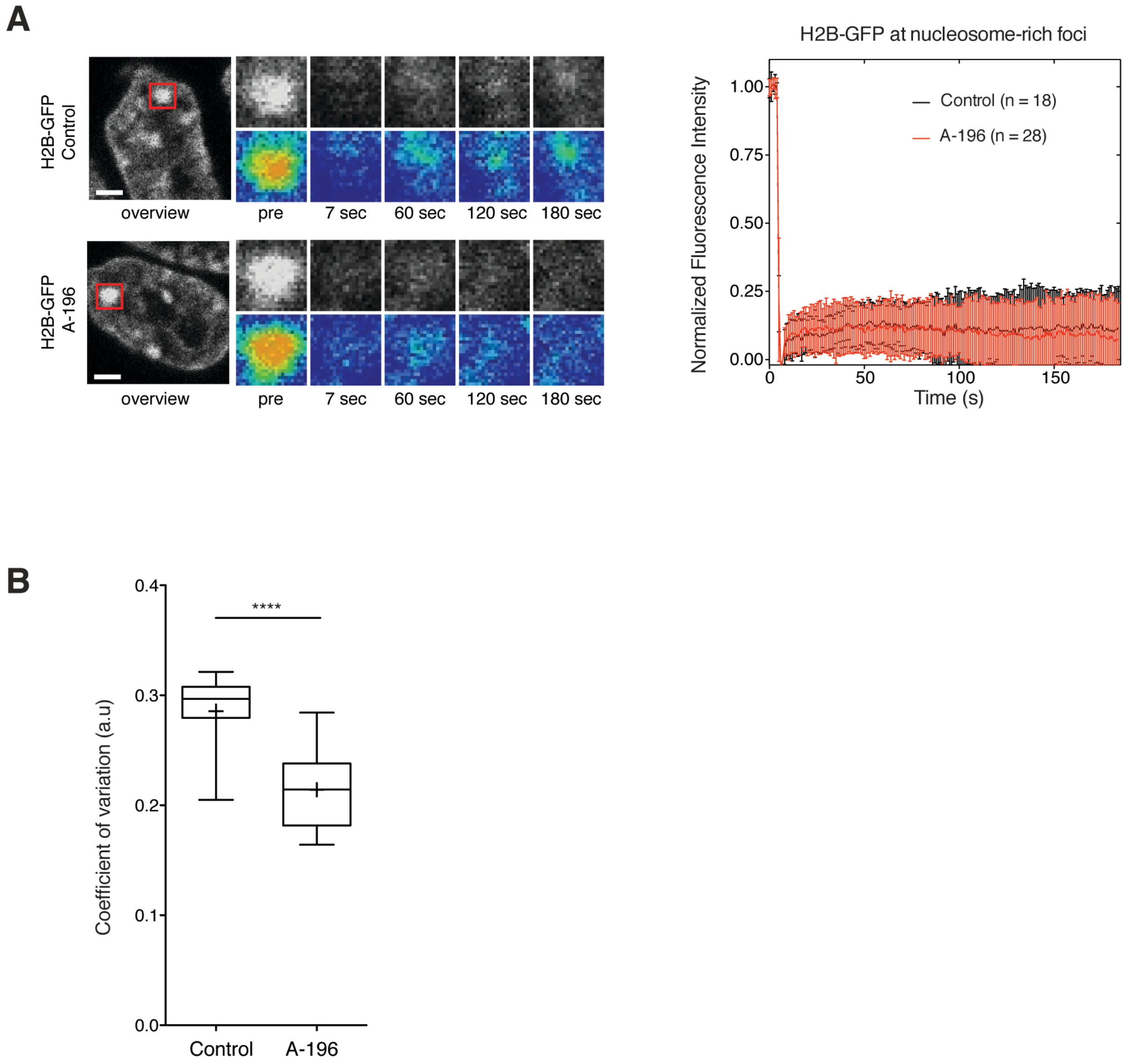

Appendix Figure S4. Characterization of the SUV4-20 H1/2 inhibitor A-196 treatment on H2B-GFP dynamics by FRAP and chromatin compaction using CV analysis in ESCs.

- **A.** Chromocenter bleach for ES cells expressing H2B-GFP in control condition (top panels) or upon SUV4-20 inhibitor A-196 (bottom panels). n, number of chromocenters analysed.
- **B.** The coefficient of variation (CV) is calculated in control ESCs or following SUV4-20 inhibitor A-196 treatment. ****, $p < 0{,}0001$, unpaired t test.
