# Supplementary material for: Evidence for low nanocompaction of heterochromatin in living embryonic stem cells
Source: EMBO J. 2023 Apr 21;42(12):e110286. doi: 10.15252/embj.2021110286 (PMC10267699; doi:10.15252/embj.2021110286)
Supplement: Supplementary file 2 — Expanded View Figures PDF [file EMBJ-42-e110286-s010.pdf]

Expanded View Figures

Figure EV1. Characterisation of BJ $^{H2B\text{-}2FPs}$ ESCs suitable to study chromatin nanoscale organisation.

A Colony morphology and co-expression of fluorophore-tagged H2Bs in BJ ESCs. Scale bars, 20 μm. The GFP and mCherry fluorescence intensities from different ESC colonies are compared (bottom graphs). The data are presented as means and the error bars represent the standard deviations ($n = 4$ technical independent samples). Each colony contains around 20 cells.

B DAPI staining on BJ $^{H2B\text{-}2FPs}$ ESCs in interphase and after mitotic spread. Line scan intensity is indicated by a white line. Scale bars, 10 μm. Fluorescence intensity profiles from this interphase nucleus are displayed below.

C Spearman correlation between DAPI and mCherry fluorescence intensities (left panel), between DAPI and GFP fluorescence intensities (middle panel) and between GFP and mCherry fluorescence intensities (right panel) obtained from BJ $^{H2B\text{-}2FPs}$ ESCs (Each correlation, $n = 65$ cells).

D Quantitative FRAP analysis of H2B-GFP histones from BJ $^{H2B\text{-}2FPs}$ and BJ $^{H2B\text{-}GFP}$ ESCs.

E Chromatin fractions from BJ$^{H2B\text{-}2FPs}$ ESCs analysed by western blotting with antisera against H2B (short and long exposure), mCherry and GFP. An antiserum against the histone H3 was used as a loading control. The ratios between tagged H2B and total histone H2B are calculated from three western blotting experiments from three biological replicates.

F Quantification of the nuclei area (μm^2) of wild-type BJ ESCs (BJ WT) and BJ $^{H2B\text{-}2FPs}$ ESCs. The box plots indicate the median values (middle lines), first and third quartiles (box edges) and the whiskers cover the minimum to maximum value range of nucleus area (μm^2). Data are means of $n = 3$ biological replicates. The number of nuclei analysed is between 50–100. Significance values are computed using Mann–Whitney test, ns = $P > 0.05$.

G Representative images of immunostaining for POU5F1 (OCT-4) in BJ WT (top panels) and BJ $^{H2B\text{-}2FPs}$ ESCs (bottom panels). Scale bars, 10 μm.

H Proliferation curves of the parental BJ WT (blue, $n = 3$) and BJ $^{H2B\text{-}2FPs}$ ESCs (red, $n = 3$).

© 2023 The Authors

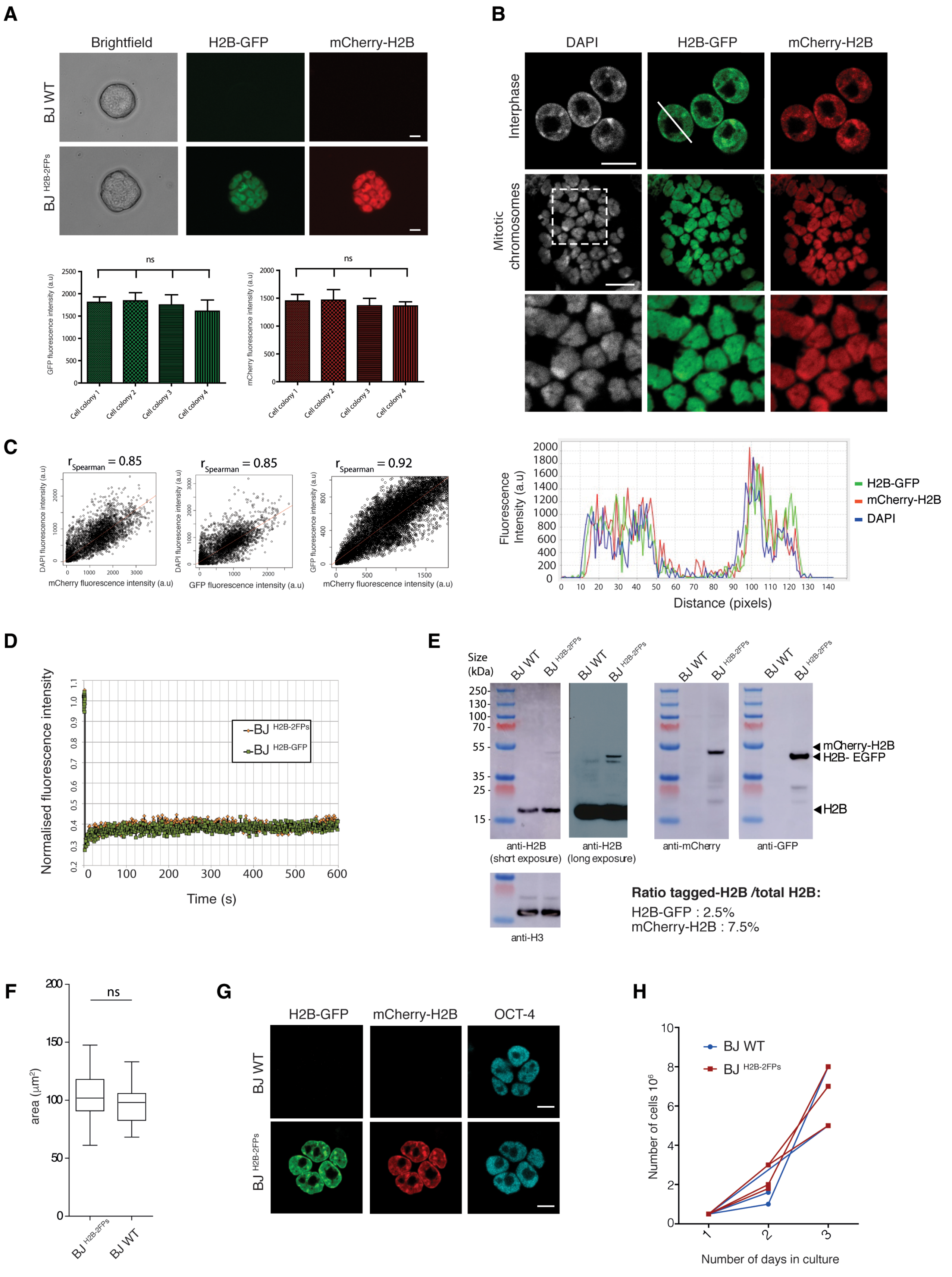

Figure EV1.

 © 2023 The Authors

Figure EV2. FRET efficiency depends on the proximity between nucleosomes.

A *In vivo* FLIM-FRET assay in BJ $^{H2B\text{-}GFP}$ and BJ $^{H2B\text{-}2FPs}$ ESCs (top and bottom panels, respectively). The mean GFP fluorescence lifetime (τ) is displayed using a continuous pseudo-colour scale from 2,100 to 2,250 ps. Scale bars, 5 μm.

B Histogram of the H2B-GFP fluorescence lifetime from BJ $^{H2B\text{-}GFP}$ ($n = 100$ cells) and BJ $^{H2B\text{-}2FPs}$ ESCs ($n = 101$ cells). The data are presented as means and the error bars represent standard deviations. ****$P < 0.0001$, Mann–Whitney test.

C Comparison of nuclei area between untreated ($n = 153$ cells), TSA-treated ($n = 101$ cells) and ATP-depleted ($n = 61$ cells) BJ $^{H2B\text{-}2FPs}$ ESCs. The data are presented as means, and the error bars represent standard deviations. ns, $P = 0.16$ for untreated/TSA, $P = 0.50$ for untreated/ATP-depleted, Mann–Whitney test.

D *In vivo* FLIM-FRET assay on BJ $^{H2B\text{-}2FPs}$ cells in metaphase. The mean FRET efficiency is displayed using a continuous pseudo-colour scale from 0 to 40%. Scale bar, 5 μm.

E Mean distribution of the FRET efficiency (%) related to the pixel fraction from BJ $^{H2B\text{-}2FPs}$ ESCs at interphase (blue, $n = 384$ cells) and metaphase (orange, $n = 56$ cells). ****, $P = 2.2e\text{-}16$; K–S test.

F Total cell extracts from untreated and TSA-treated BJ $^{H2B\text{-}2FPs}$ ESCs analysed by western blotting with an antiserum against acetyl-H3 (short and long exposure). Loading control was performed by red ponceau staining.

G Schematic presentation of the developed FRENETIC (FREt Nucleosome EpigeneTic Image Correlation) workflow to correlate FRET measurements and fluorescence intensity from tagged proteins in living cells.

H Comparison of the distributions of the "Nucleosome-rich foci" and "All nuclei pixels" data (see Fig 2E). Blue circles represent data between 0–12% FRET efficiencies, green circles 12–25%, red circles 25–40%.

Source data are available online for this figure.

© 2023 The Authors

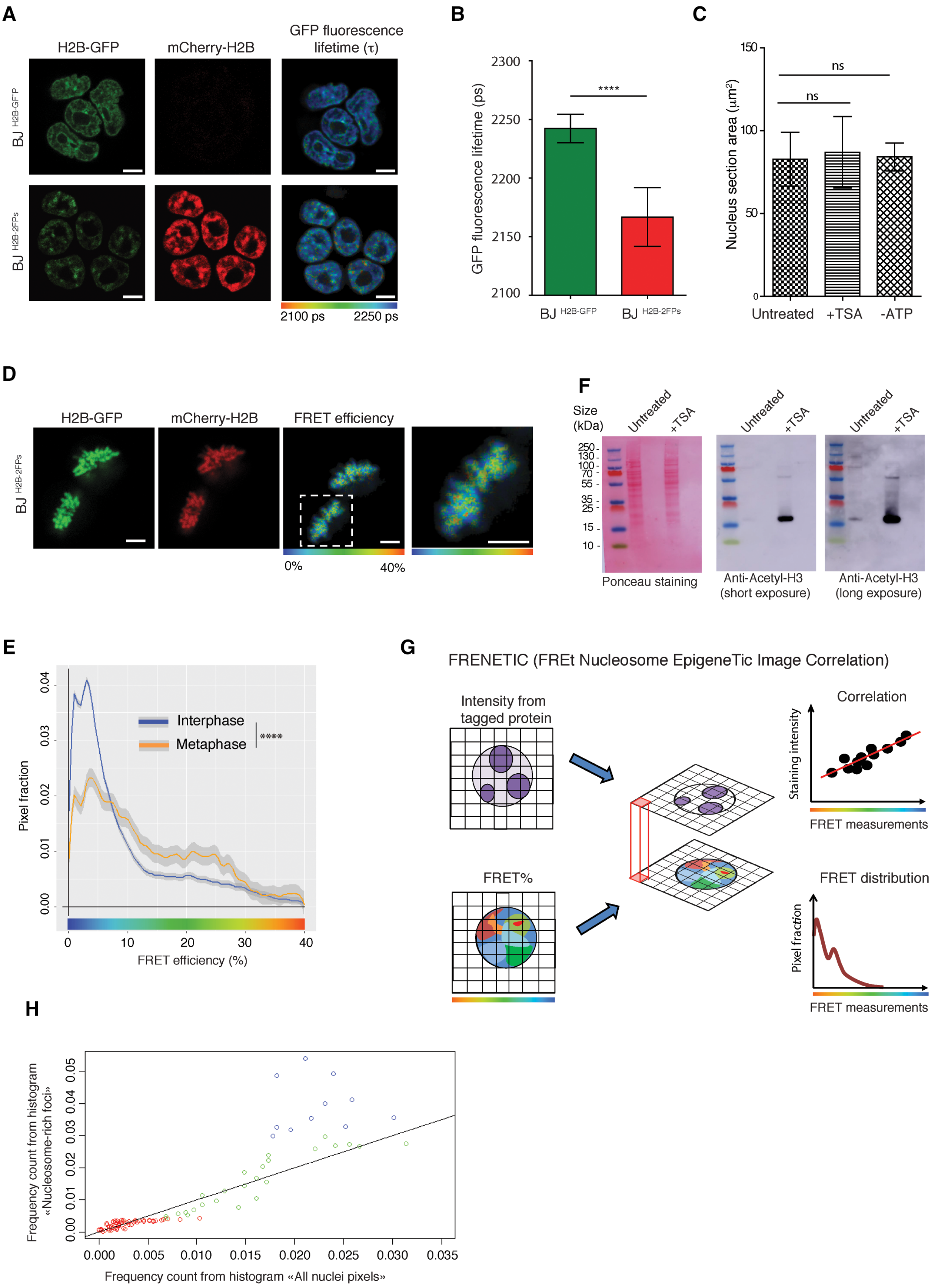

Figure EV2.

 © 2023 The Authors

Figure EV3. Cell fixation alters the nucleosomal organisation of chromatin.

A Mean distribution of the FRET efficiencies from living ESCs (green, $n = 384$ cells) and fixed cells (black, $n = 157$ cells). ****$P = 2.2e\text{-}16$; K–S test.

B Box-and-whisker plots of the mean FRET efficiency from living and fixed ESCs. The box plots indicate the median values (middle lines), first and third quartiles (box edges) and the whiskers cover the minimum to maximum value range. Data are means of $n = 2$ biological replicates; $n = 18$ cells for living ESCs and $n = 19$ cells for fixed ESCs. ****$P < 0.0001$, Mann–Whitney test.

C Quantification of the nuclear volume of living ESCs and 4% PFA-fixed ESCs. The Box-and-Whisker plots indicate median values (middle lines), first and third quartiles (box edges) and the whiskers cover the 10–90 percentiles value range. Data are means of $n = 2$ biological replicates; $n = 24$ cells for living ESCs and $n = 26$ cells for 4% PFA-fixed ESCs. Statistical significance was determined by unpaired two-tailed Student's t-test, ns, $P = 0.4786$.

D Left panel, Box-and-Whisker plots representing the mean section surfaces of foci in living ($n = 384$ cells) and fixed ($n = 157$ cells) ESCs. ***$P < 0.001$, Mann–Whitney test. Right panel, boxplot of the number of foci per nucleus in living ($n = 384$ cells) and fixed ($n = 157$ cells) ESCs. ns, $P = 0.40$, Mann–Whitney test. The box plots indicate median values (middle lines), first and third quartiles (box edges) and the whiskers cover the minimum to maximum value range.

E Box-and-Whisker plots representing the mean FRET efficiency from living ESCs ($n = 23$) and fixed ESCs using different fixative procedures (4%PFA/1xPBS ($n = 16$); 2% FA/1xHBSS ($n = 19$) and methanol-ethanol (1:1) ($n = 9$)). The box plots indicate the median values (middle lines), the mean values (middle crosses), first and third quartiles (box edges) and the whiskers cover the minimum to maximum value range. ****$P < 0.0001$, unpaired two-tailed Student's t-test.

F Quantification of the H2B-GFP fluorescence intensity for living ESCs ($n = 26$) and fixed ESCs using different fixative procedures (4%PFA/1xPBS ($n = 16$); 2%FA/1xHBSS ($n = 24$) and methanol-ethanol (1:1) ($n = 13$)). The Box-and-Whisker plots indicate median values (middle lines), mean values (middle crosses), first and third quartiles (box edges) and the whiskers cover the 10–90 percentiles value range. **$P < 0.01$; ****$P < 0.0001$, unpaired two-tailed Student's t-test.

G Quantification of the mCherry-H2B fluorescence intensity for living ESCs ($n = 26$) and fixed ESCs using different fixative procedures (4%PFA/1xPBS ($n = 16$); 2%FA/1xHBSS ($n = 24$) and methanol-ethanol (1:1) ($n = 13$)). The Box-and-Whisker plots indicate median values (middle lines), mean values (middle crosses), first and third quartiles (box edges) and the whiskers cover the 10–90 percentiles value range. **$P < 0.01$; ****$P < 0.0001$; ns, $P = 0.0624$, unpaired two-tailed Student's t-test.

© 2023 The Authors

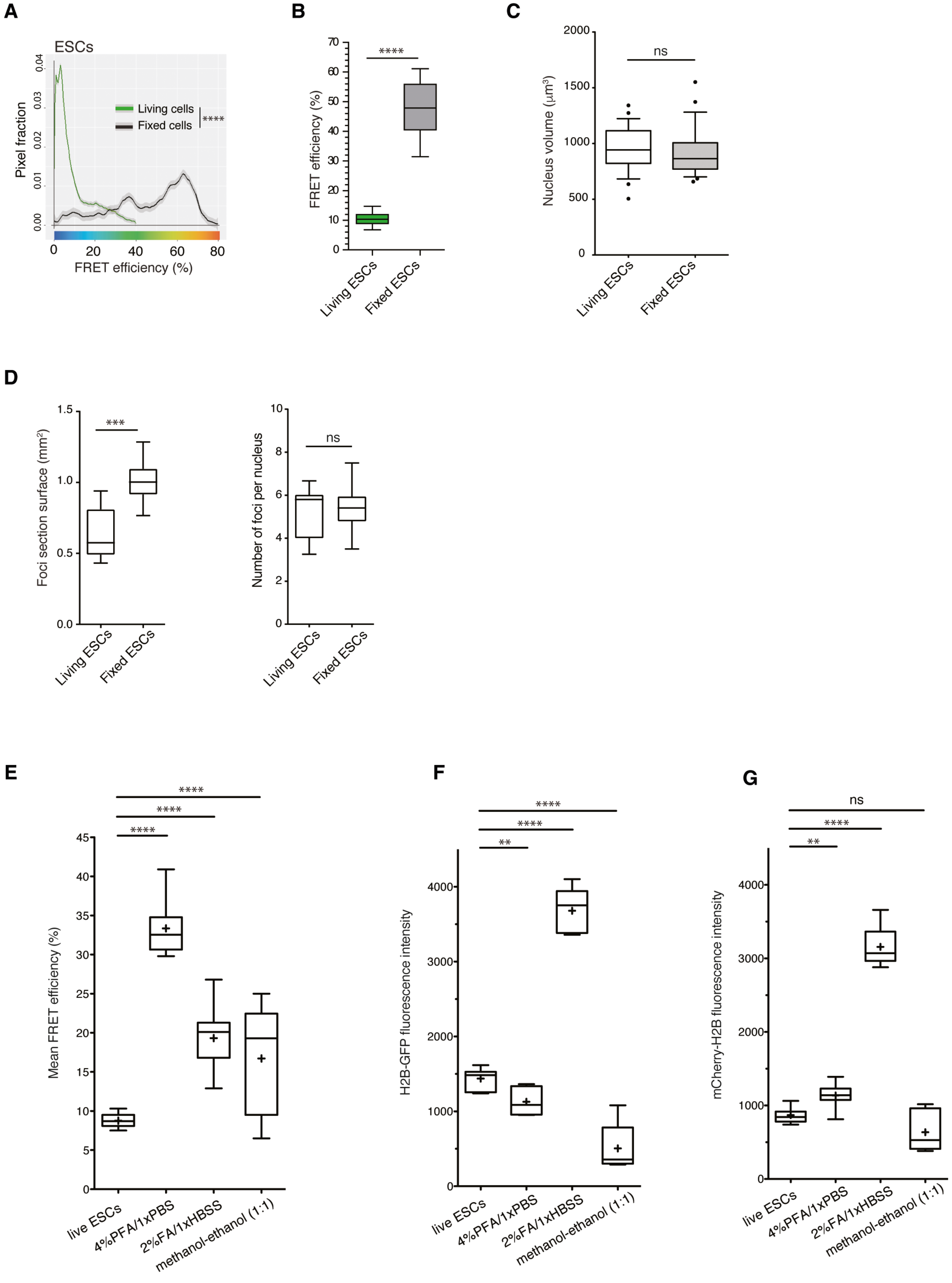

Figure EV3.

 © 2023 The Authors

Figure EV4. Characterisation of HP1α and HP1β siRNA depletions and A-196 treatment in BJ $^{H2B\text{-}2FPs}$ ESCs.

A Total cell extracts from BJ$^{H2B\text{-}2FPs}$ cells incubated during 24 h with untargeted siRNA (siRNA CTRL) and siRNA targeting HP1α, analysed by western blotting with an antiserum against HP1α (long exposure).

B Left panel, Box-and-Whisker plot indicating the number of nuclear foci in BJ$^{H2B\text{-}2FPs}$ treated with siRNA Control ($n = 180$ cells) and siRNA HP1α ($n = 170$ cells). The Box-and-Whisker plots indicate the median values (horizontal lines), first and third quartiles (box edges) and the whiskers cover the minimum to maximum value range. ns, $P = 0.96$, Mann–Whitney test. Right panel, boxplot of the mean area of individual focus in BJ$^{H2B\text{-}2FPs}$ treated with siRNA Control ($n = 180$ cells) and siRNA HP1α ($n = 170$ cells). ns, $P = 0.44$, Mann–Whitney test.

C Compaction of chromocenters was assessed by calculating the coefficient of variation (CV) of the H2B-GFP signals in ESCs treated with siRNA Control ($n = 33$ cells), siRNA HP1a ($n = 20$ cells; ns, $P = 0.2415$) and siRNA HP1a/b ($n = 20$ cells; ns, $P = 0.1105$). This method has previously been used to measure heterochromatin compaction. The distribution of values is represented by Box-and-Whisker plots indicating the median values (horizontal lines), the mean (middle crosses), first and third quartiles (box edges) and the whiskers cover the minimum to maximum value range. Statistical significance was determined by Mann–Whitney test.

D Comparison of the distributions from "siRNA Control" and "siRNA HP1α" data (see Fig 4B).

E Total cell extracts from BJ$^{H2B\text{-}2FPs}$ cells incubated during 24 h with untargeted siRNAs (CTRL) and siRNAs targeting HP1β, analysed by western blotting with an antiserum against HP1β (left panels). Loading control was assessed by western blotting with an antiserum against βactin and red ponceau staining.

F Total cell extracts from BJ$^{H2B\text{-}2FPs}$ cells incubated during 24 h with untargeted siRNAs (CTRL) and siRNAs targeting both HP1α and HP1β, analysed by western blotting with antisera against HP1β (left panels) and HP1α (right panels). Loading control was assessed by red ponceau staining.

G Box-and-Whisker plots of the H2B-GFP/mCherry-H2B intensity ratios in untreated ESCs (white box, $n = 13$ cells) and ESCs treated with A-196 during 2 days (grey box, $n = 11$ cells). The Box-and-Whisker plots indicate the median values (horizontal lines), first and third quartiles (box edges) and the whiskers cover the minimum to maximum value range. ns, $P = 0.06$, Mann–Whitney test.

H Total cell extracts from untreated cells or A-196 treated BJ$^{H2B\text{-}2FPs}$ cells during 48 h analysed by western blotting with antisera against H4K20me2 (middle panel), or H3K9me3 (right panel). Loading control was assessed by western blotting with an antiserum against βactin (left panel).

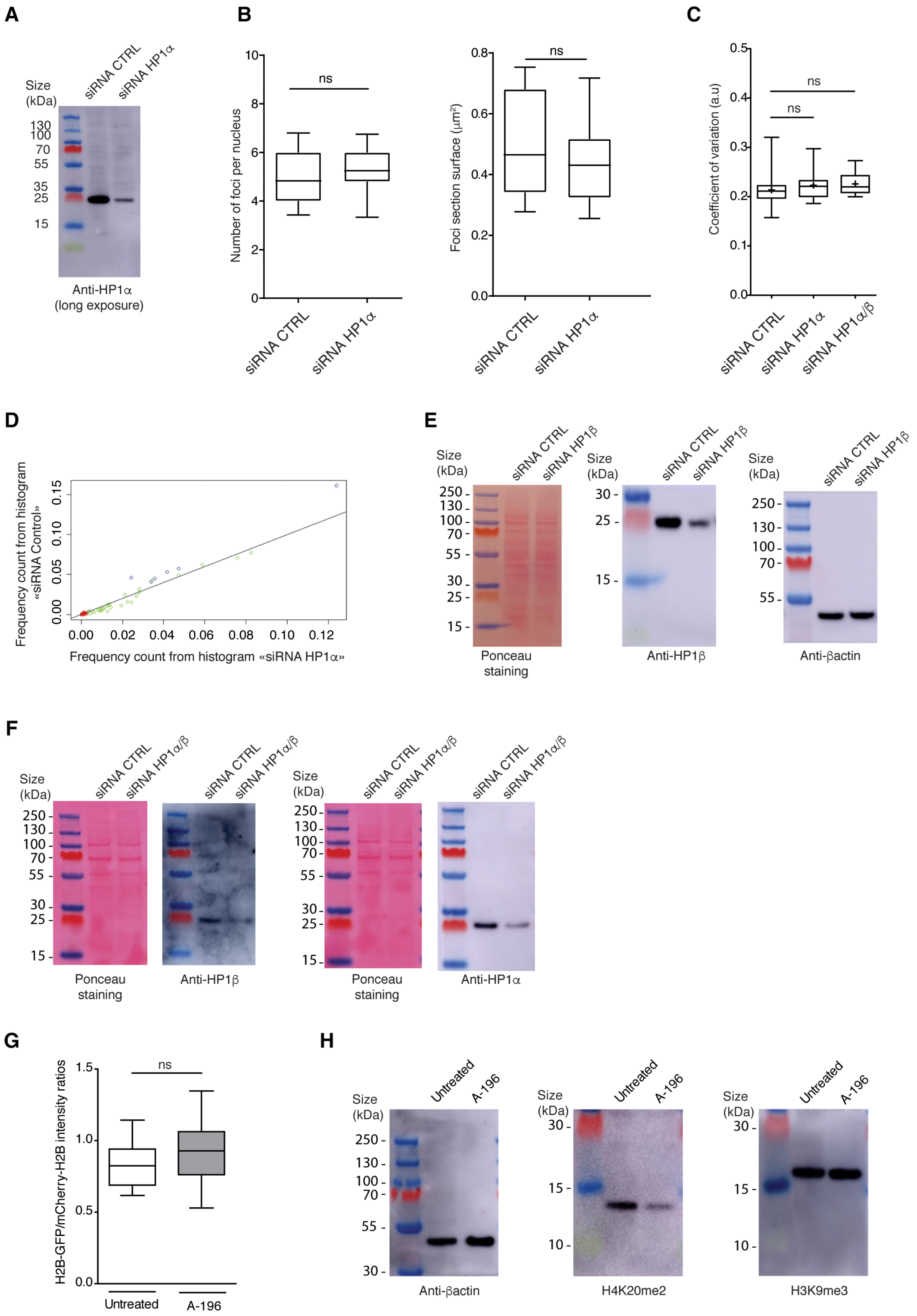

Figure EV4.

 © 2023 The Authors

Figure EV5. Characterisation of CRISPR/Cas9-mediated *Mki67*$^{-/-}$ ES clones.

A Location of the CRISPR-Cas9-mediated disruption of the *Mki67* gene in mouse ES cells (targeting exon 3 and resulting in a 5 nt deletion) and sequencing assessment of the deletion in the *Mki67*$^{-/-}$ clones generated.

B Expression levels of *Mki67* mRNA in parental control ESCs and in three *Mki67*$^{-/-}$ ESC clones.

C Western blotting analysis of the indicated proteins in wild-type ESCs (WT), control ESCs (CTRL), and *Mki67*$^{-/-}$ clone #1, clone #2 and clone #3.

D Top panels, representative images of Immunostaining for Ki-67 in WT ESCs. Bottom panels, control immunostaining with only the secondary antibody in WT ESCs. Scale bar, 10 μm.

E Box-and-Whisker plot representation of the H2B-GFP/mCherry-H2B intensity ratios in control ESCs (white box, $n = 13$ cells) and *Mki67*$^{-/-}$ clone #1 (grey box, $n = 15$ cells). The Box-and-Whisker plots indicate the median values (horizontal lines), the mean values (middle crosses), first and third quartiles (box edges) and the whiskers cover the minimum to maximum value range. ns, $P = 0.0792$, Mann–Whitney test.

F Left panel, Pearson correlation coefficient represented as a Box-and-Whisker plot between H3K9me3 and H2B-GFP in control ESCs (white box, $n = 5$ ESC colonies) and *Mki67*$^{-/-}$ clone #1 (grey box, $n = 4$ ESC colonies). The Box-and-Whisker plots indicate the median values (horizontal lines), first and third quartiles (box edges) and the whiskers cover the minimum to maximum value range. $P = 0.2204$, unpaired t-test. Right panel, Pearson correlation coefficient represented as a box plot between HP1α and H2B-GFP in control ESCs (white box, $n = 6$ ESC colonies) and *Mki67*$^{-/-}$ clone #1 (grey box, $n = 6$ ESC colonies). $P = 0.2547$, unpaired t-test.

© 2023 The Authors

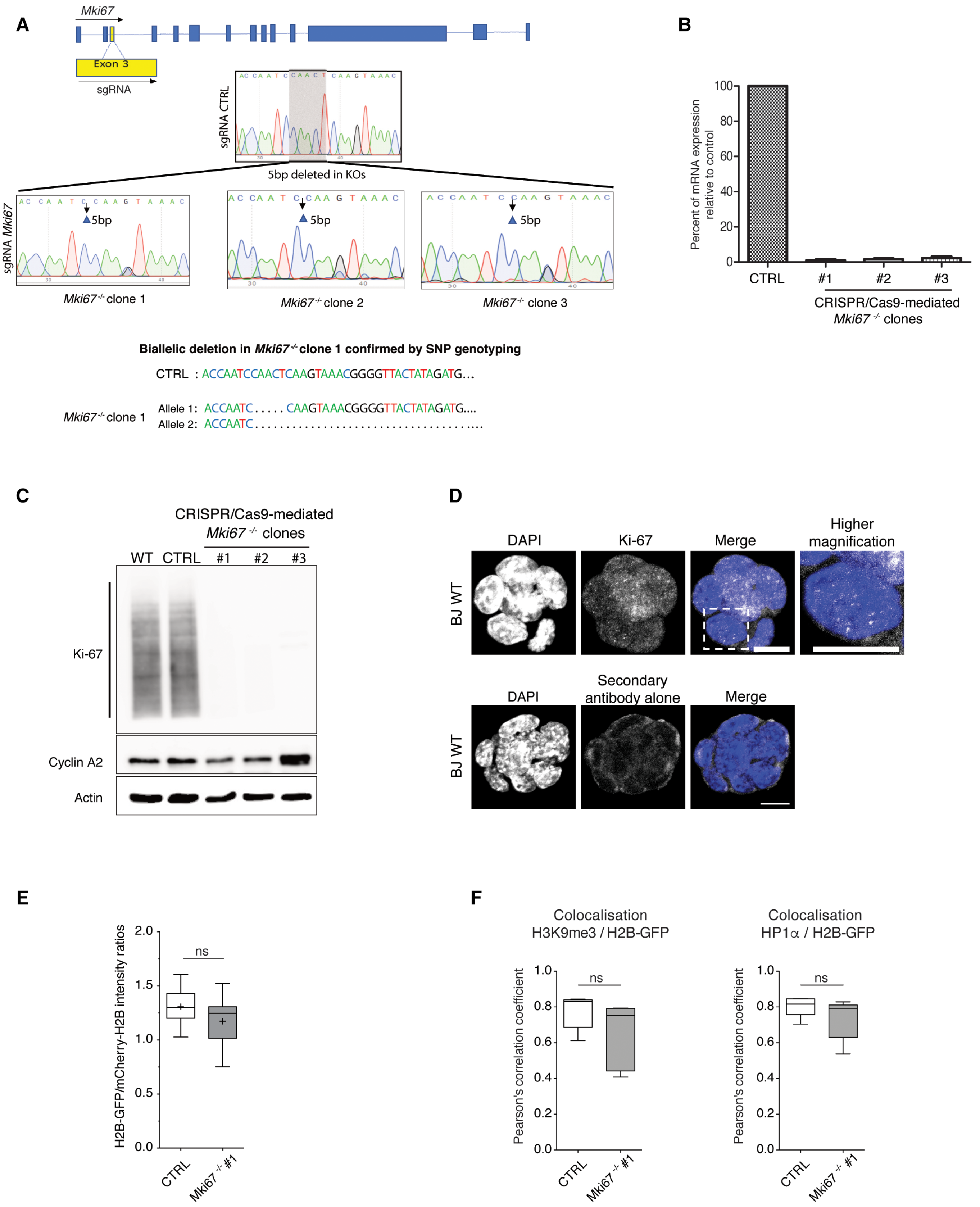

Figure EV5.

© 2023 The Authors

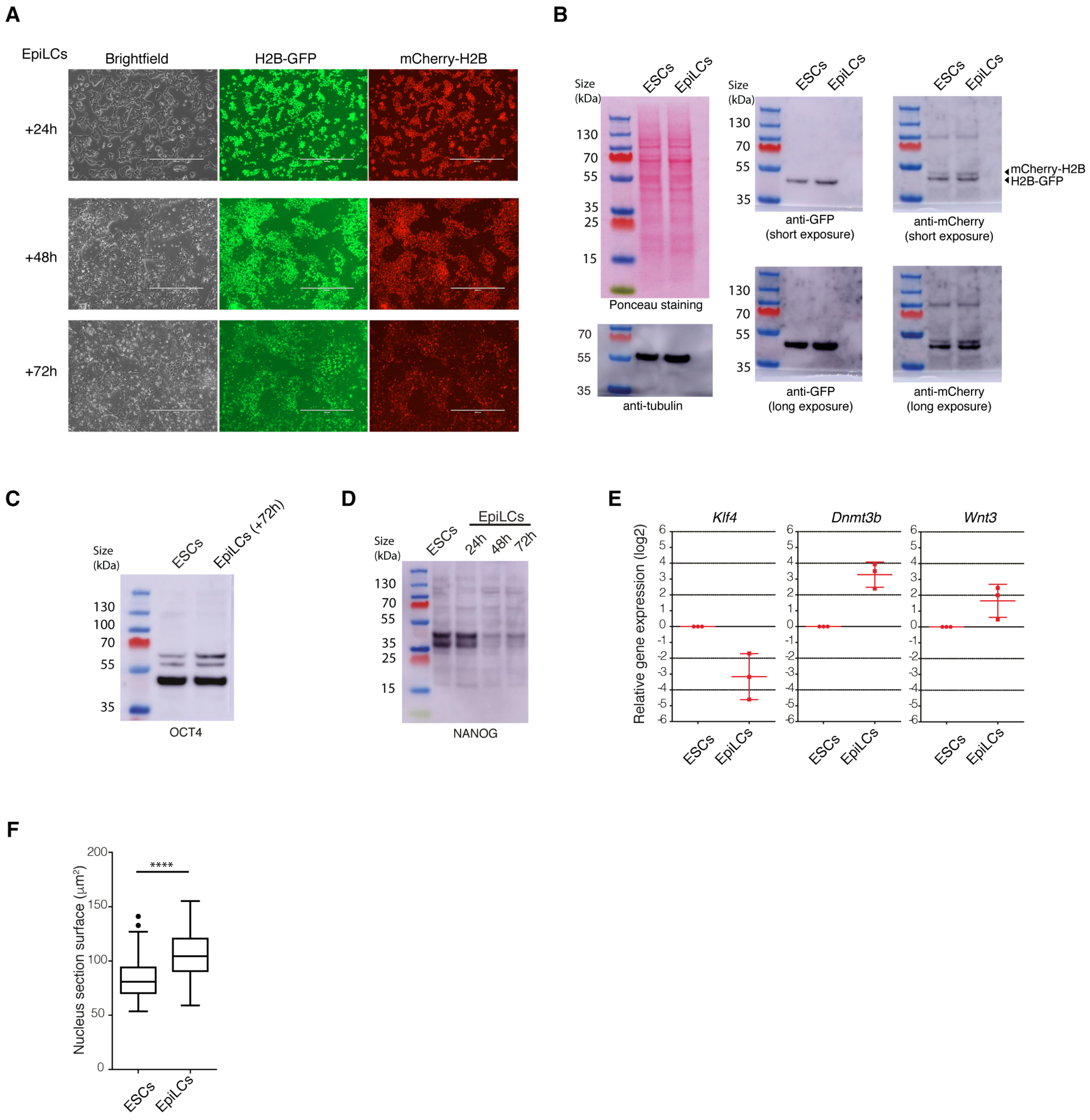

Figure EV6. Characterisation of the early differentiation of BJ$^{H2B\text{-}2FPs}$ ESCs into EpiLCs.

A EpiLCs induction from BJ$^{H2B\text{-}2FPs}$ ESCs. Bright-field and fluorescence images from the H2B-GFP and mCherry-H2B reporters are shown. Scale bars, 400 µm.

B Total cell extracts from naive and EpiLCs BJ$^{H2B\text{-}2FPs}$ (+72 h) analysed by western blotting with antisera against GFP and mCherry. Loading control was assessed by ponceau staining and by western blotting with an antiserum against tubulin.

C Total cell extracts from naive and EpiLCs BJ$^{H2B\text{-}2FPs}$ (+72 h) analysed by western blotting with an antiserum against POU5F1.

D Total cell extracts from naive and EpiLCs BJ$^{H2B\text{-}2FPs}$ at different time points after induction analysed by western blotting with an antiserum against NANOG.

E Gene expression profiles during EpiLCs induction measured by RT-qPCR. For each gene (*Klf4*, *Dnmt3b* and *Wnt3*), the ΔCT was calculated from two housekeeping genes *Arbp* and *Ppia*. The values are presented on the log2 scale, with ESC values were set up at 0. Data are means of $n = 3$ biological replicates and the error bars represent standard deviations.

F Box-and-Whisker plot representation of the nucleus section surface from ESCs ($n = 46$ cells) and EpiLCs ($n = 27$ cells). The Box-and-Whisker plots indicate median values (horizontal lines), first and third quartiles (box edges) and the whiskers cover the 10–90 percentiles value range. ****$P < 0.0001$, Mann–Whitney test.
