# Supplementary material for: Evidence for low nanocompaction of heterochromatin in living embryonic stem cells
Source: EMBO J. 2023 Apr 21;42(12):e110286. doi: 10.15252/embj.2021110286 (PMC10267699; doi:10.15252/embj.2021110286)
Supplement: Supplementary file 3 — Table EV1 [file EMBJ-42-e110286-s007.docx]

**Table EV1. Gene expression /sequencing PCR oligonucleotides.**

| Genes | Primer sequences (5’-3’) |
| --- | --- |
| *Ppia* | FW: TTA CCC ATC AAA CCA TTC CTT CTG  RV: AAC CCA AAG TTC AGT GAGA AGC |
| *Arbp* | FW: CAA AGC TGA AGC AAA GGA AGA  RV: AAT TAA GCA GGC TGA CTT GGT TG |
| *Wnt3* | FW: CAA GCA CAA CAA TGA AGC AGG C  RV: TCG GGA CTC ACG GTG TTT CTC |
| *Dnmt3b* | FW: CTC GCA AGG TGT GGG CTT TTG TAA C  RV: CTG GGC ATC TGT CAT CTT TGC ACC |
| *Klf4* | FW: TGG TGC TTG GTG AGT TGT GG  RV: GCT CCC CCG TTT GGT ACC TT |
| *Mki67* | FW: AATCCAACTCAAGTAAACGGGG  RV: TTGGCTTGCTTCCATCCTCA |
| *Mki67*_Gene_Seq | FW: GTC CTT AGT GTT TGC CTC  RV: AGA ACT CAT CCA GTT GGC |
