# Supplementary material for: Evidence for low nanocompaction of heterochromatin in living embryonic stem cells
Source: EMBO J. 2023 Apr 21;42(12):e110286. doi: 10.15252/embj.2021110286 (PMC10267699; doi:10.15252/embj.2021110286)
Supplement: Supplementary file 4 — Table EV2 [file EMBJ-42-e110286-s008.docx]

**Table EV2. ChIP-qPCR**

| Genes | Primer sequences (5’-3’) |
| --- | --- |
| *IAPs* | FW: GCA CCC TCA AAG CCT ATC TTA  RV: TCC CTT GGT CAG TCT GGA TTT |
| *Major Sat* | FW: TGG AAT ATG GCG AGA AAA CTG  RV: AGG TCC TTC AGT GGG CAT TT |
| *ETnERV2* | FW: ACA AAT TCA GTA TGG GCA TC  RV: GGG TAC TGT TAA GAC CCA CA |
| *Hoxa11* | FW: AGG AGA AGG GGT TCC TTC AA  RV: CTC CGC GGT TTG TCA ATA AT |
| *Actb* | FW: TTC GCT CTC TCG TGG CTA GT  RV: GAC CCT AGT GTG TCC CCA AG |
| *Pou5f1 (gene body)* | FW: TCT TTC CAC CAG GCC CCC GGC TC  RV: TGC GGG CGG ACA TGG GGA GAT CC |
| *Pou5f1 (promoter)* | FW: GGG TGG GTA AGC AAG AACT  RV: AAT GTT CGT GTG CCA ATT A |
| *Gapdh* | FW: GGA GCG AGA CCC CAC TAA CA  RV: ACA TAC TCA GCA CCG GCC TC |
