# Supplementary material for: Evidence for low nanocompaction of heterochromatin in living embryonic stem cells
Source: EMBO J. 2023 Apr 21;42(12):e110286. doi: 10.15252/embj.2021110286 (PMC10267699; doi:10.15252/embj.2021110286)
Supplement: Supplementary file 5 — Table EV3 [file EMBJ-42-e110286-s005.docx]

**Table EV3. Labelling percentage of nucleosomes**

Fraction of tagged-H2B / total H2B histones:

H2B-GFP: 0.025

mCherry-H2B: 0.075

non tagged-H2B: 0.90

| 1^st^ histone H2B | 2^nd^ histone H2B | Nucleosomes (%) |
| --- | --- | --- |
| Endogenous: 0.90 | Endogenous: 0.90 | 81 |
| Endogenous: 0.90 | GFP tagged: 0.025 | 2.25 |
| Endogenous: 0.90 | mCherry tagged: 0.075 | 6.75 |
| GFP tagged: 0.025 | Endogenous: 0.90 | 2.25 |
| GFP tagged: 0.025 | GFP tagged: 0.025 | 0.06 |
| GFP tagged: 0.025 | mCherry tagged: 0.075 | 0.19 |
| mCherry tagged: 0.075 | Endogenous: 0.90 | 6.75 |
| mCherry tagged: 0.075 | GFP tagged: 0.025 | 0.19 |
| mCherry tagged: 0.075 | mCherry tagged: 0.075 | 0.56 |

| Nucleosomes with no tagged-H2B | 81% |
| --- | --- |
| Nucleosomes with only H2B-GFP | 4.56% |
| Nucleosomes with only mCherry-H2B | 14.06% |
| Nucleosomes with both H2B-GFP and mCherry-H2B | 0.38% |
