# Supplementary figures and images for: Evidence for low nanocompaction of heterochromatin in living embryonic stem cells
Source: EMBO J. 2023 Apr 21;42(12):e110286. doi: 10.15252/embj.2021110286 (PMC10267699; doi:10.15252/embj.2021110286)

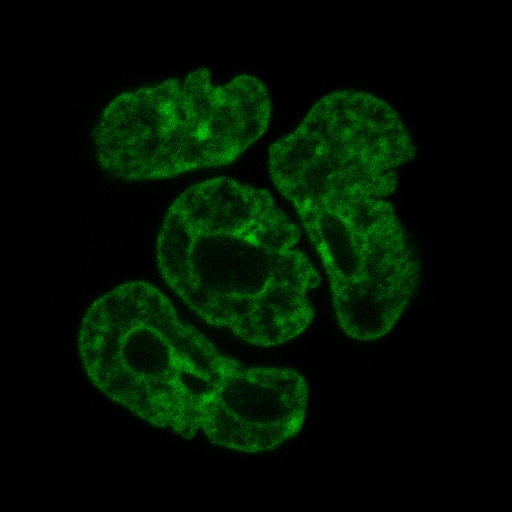

Supplement: Supplementary file 6 — Source Data for Expanded View [file EMBJ-42-e110286-s003.zip › Figure EV2/EV2A/0_BJ_H2B_GFP_9.tiff]

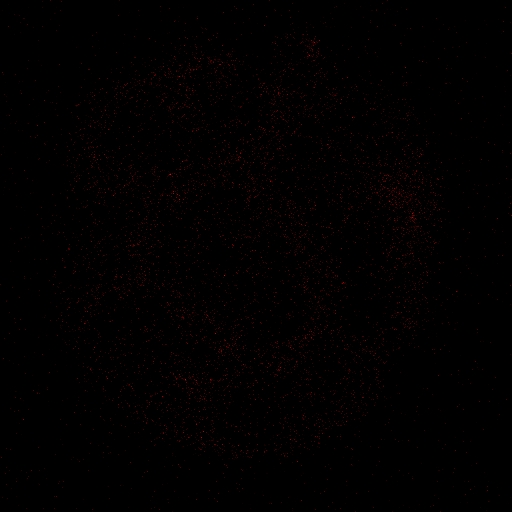

Supplement: Supplementary file 6 — Source Data for Expanded View [file EMBJ-42-e110286-s003.zip › Figure EV2/EV2A/1_BJ_H2B_GFP_9.tiff]

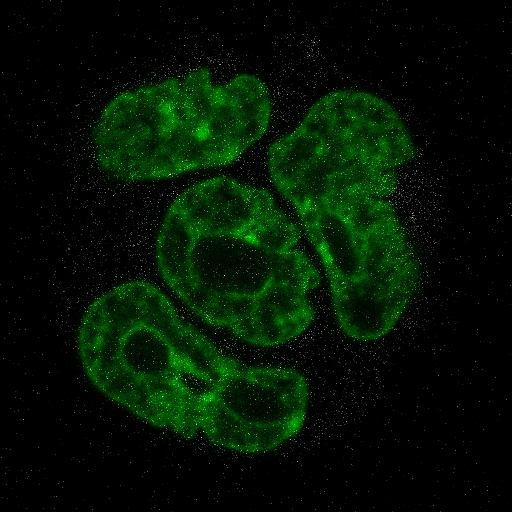

Supplement: Supplementary file 6 — Source Data for Expanded View [file EMBJ-42-e110286-s003.zip › Figure EV2/EV2A/2_BJ_H2B_GFP_9.tiff]

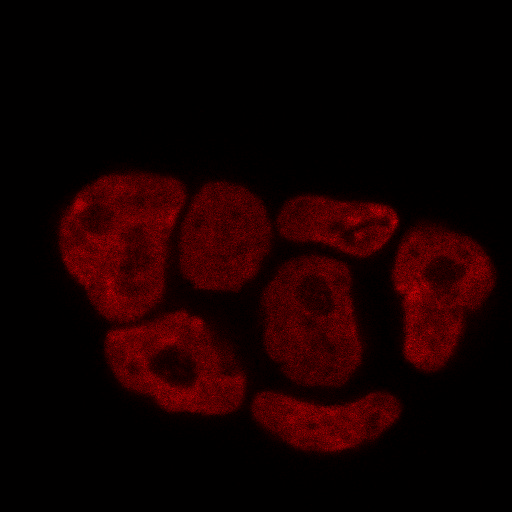

Supplement: Supplementary file 8 — Source Data for Figure 1 [file EMBJ-42-e110286-s002.zip › Figure 1/1H/Micros_image/BJ_H2B_2FPs_TSA_treatment_red channel.tif]

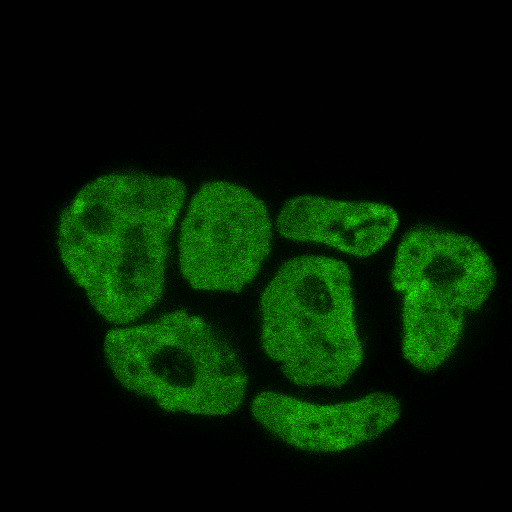

Supplement: Supplementary file 8 — Source Data for Figure 1 [file EMBJ-42-e110286-s002.zip › Figure 1/1H/Micros_image/BJ_H2B_2FPs_TSA_treatment_green channel.tif]

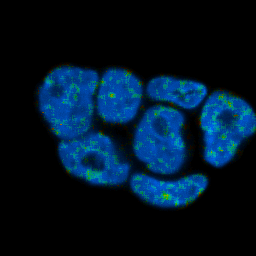

Supplement: Supplementary file 8 — Source Data for Figure 1 [file EMBJ-42-e110286-s002.zip › Figure 1/1H/Micros_image/BJ_H2B_2FPs_TSA_treatment_FRET efficiency.tif]

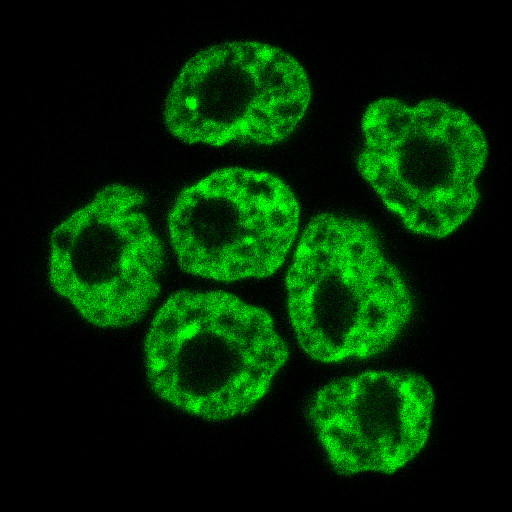

Supplement: Supplementary file 8 — Source Data for Figure 1 [file EMBJ-42-e110286-s002.zip › Figure 1/1F/ATP depletion/BJ_H2B_2FPs_atp depletion_bis green channel.tif]

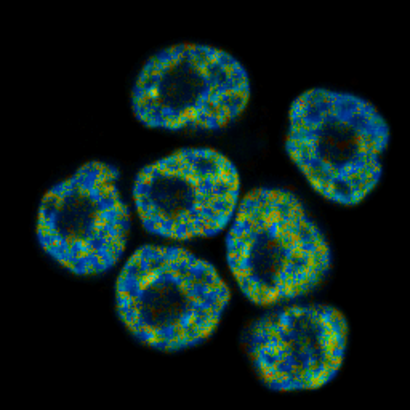

Supplement: Supplementary file 8 — Source Data for Figure 1 [file EMBJ-42-e110286-s002.zip › Figure 1/1F/ATP depletion/BJ_H2B_2FPs_atp depletion_FRET efficiency.tif]

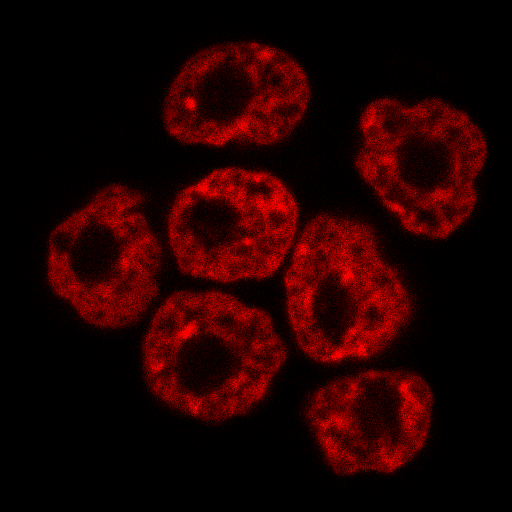

Supplement: Supplementary file 8 — Source Data for Figure 1 [file EMBJ-42-e110286-s002.zip › Figure 1/1F/ATP depletion/BJ_H2B_2FPs_atp depletion_red channel.tif]

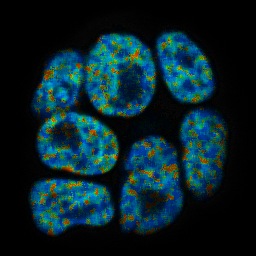

Supplement: Supplementary file 8 — Source Data for Figure 1 [file EMBJ-42-e110286-s002.zip › Figure 1/1F/Untreated_ESCs/BJ_H2B_2FPs_untreated_FRET efficiency.tif]

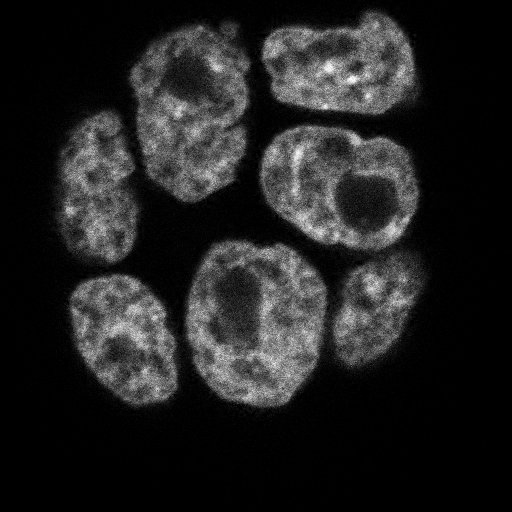

Supplement: Supplementary file 8 — Source Data for Figure 1 [file EMBJ-42-e110286-s002.zip › Figure 1/1F/Untreated_ESCs/BJ_H2B_2FPs_untreated_red channel.tif]

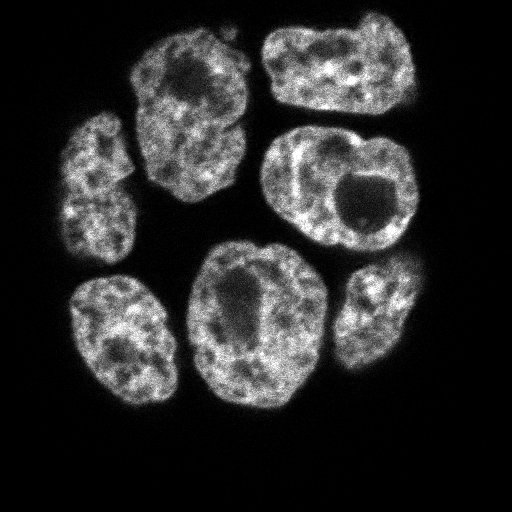

Supplement: Supplementary file 8 — Source Data for Figure 1 [file EMBJ-42-e110286-s002.zip › Figure 1/1F/Untreated_ESCs/BJ_H2B_2FPs_untreated_green_channel.tif]

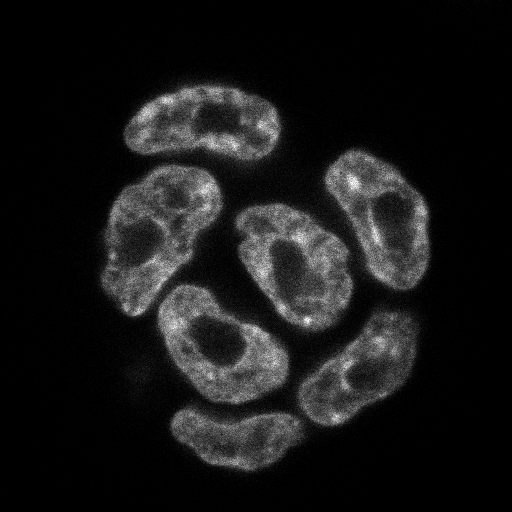

Supplement: Supplementary file 8 — Source Data for Figure 1 [file EMBJ-42-e110286-s002.zip › Figure 1/1B/BJ_H2B_2FPs_mCherry_channel.tif]

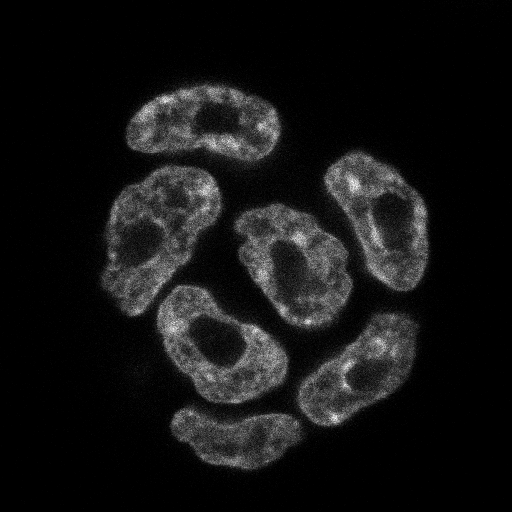

Supplement: Supplementary file 8 — Source Data for Figure 1 [file EMBJ-42-e110286-s002.zip › Figure 1/1B/BJ_H2B_2FPs_GFP_channel.tif]

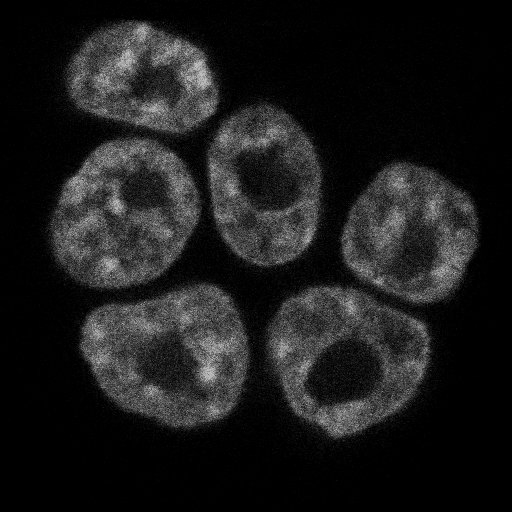

Supplement: Supplementary file 8 — Source Data for Figure 1 [file EMBJ-42-e110286-s002.zip › Figure 1/1E/BJ_H2B_2FPs_GFP channel.tif]

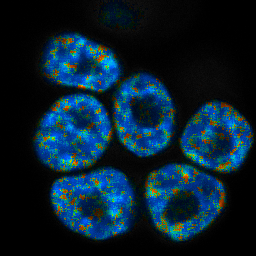

Supplement: Supplementary file 8 — Source Data for Figure 1 [file EMBJ-42-e110286-s002.zip › Figure 1/1E/BJ_H2B_2FPs_FRET efficiency.tif]

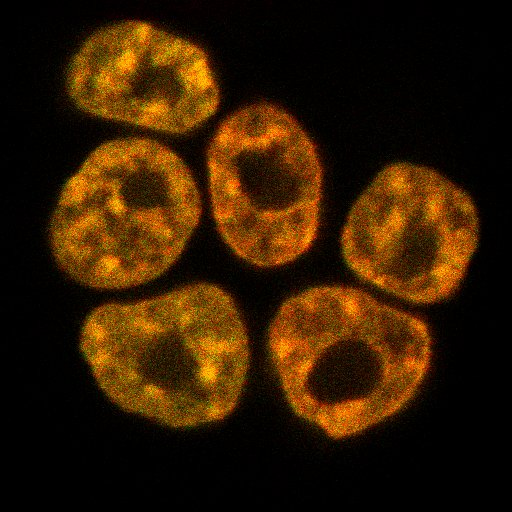

Supplement: Supplementary file 8 — Source Data for Figure 1 [file EMBJ-42-e110286-s002.zip › Figure 1/1E/BJ_H2B_2FPs_merge.tif]

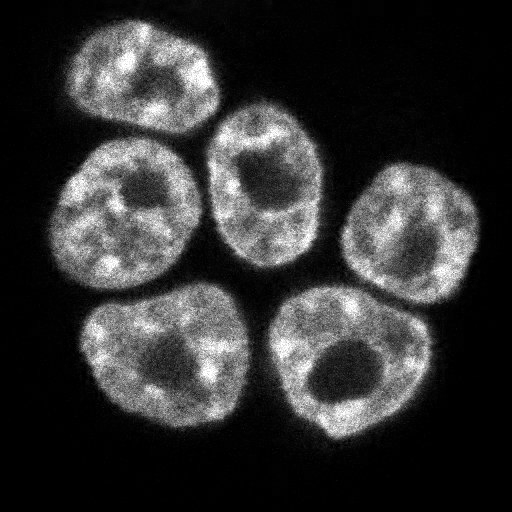

Supplement: Supplementary file 8 — Source Data for Figure 1 [file EMBJ-42-e110286-s002.zip › Figure 1/1E/BJ_H2B_2FPs_mcherry channel.tif]

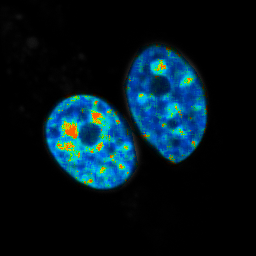

Supplement: Supplementary file 9 — Source Data for Figure 2 [file EMBJ-42-e110286-s004.zip › Figure 2/2G/3T3_FRET efficiency.tif]

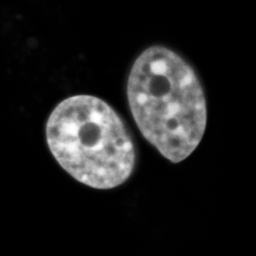

Supplement: Supplementary file 9 — Source Data for Figure 2 [file EMBJ-42-e110286-s004.zip › Figure 2/2G/3t3_GFP intensity.tif]

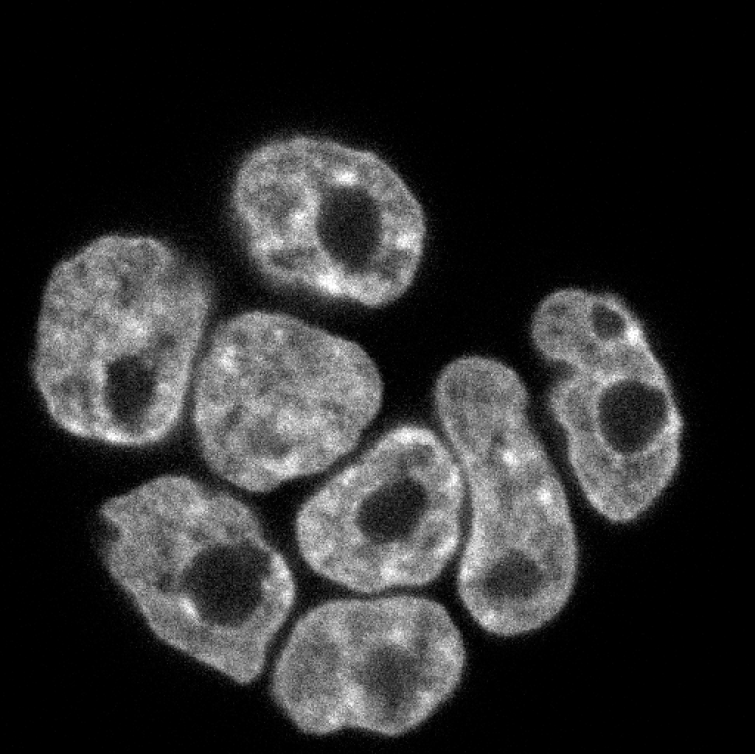

Supplement: Supplementary file 9 — Source Data for Figure 2 [file EMBJ-42-e110286-s004.zip › Figure 2/2A/ESCs_GFP intensity.tif]

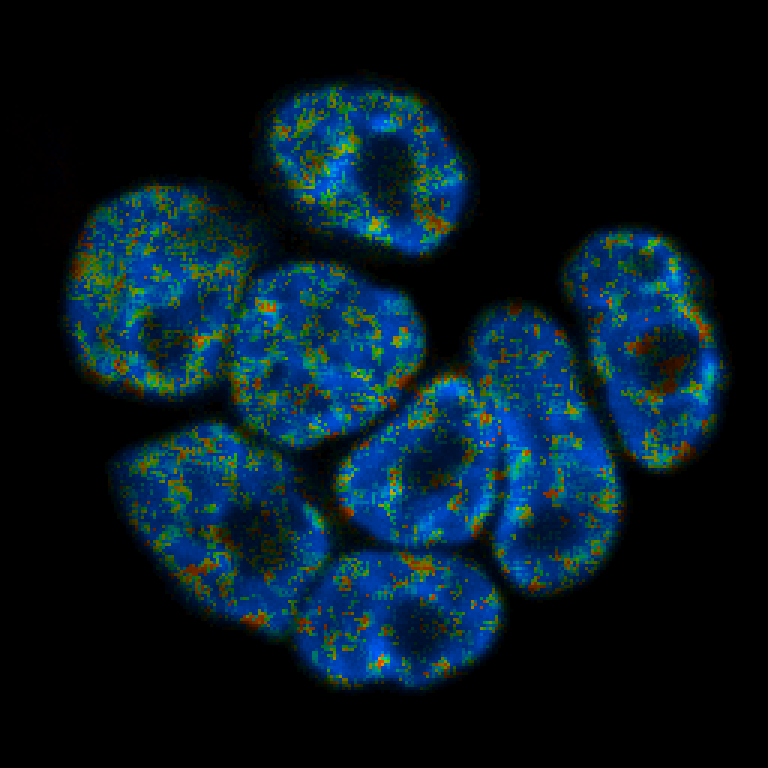

Supplement: Supplementary file 9 — Source Data for Figure 2 [file EMBJ-42-e110286-s004.zip › Figure 2/2A/ESCs_FRET efficiency.tif]

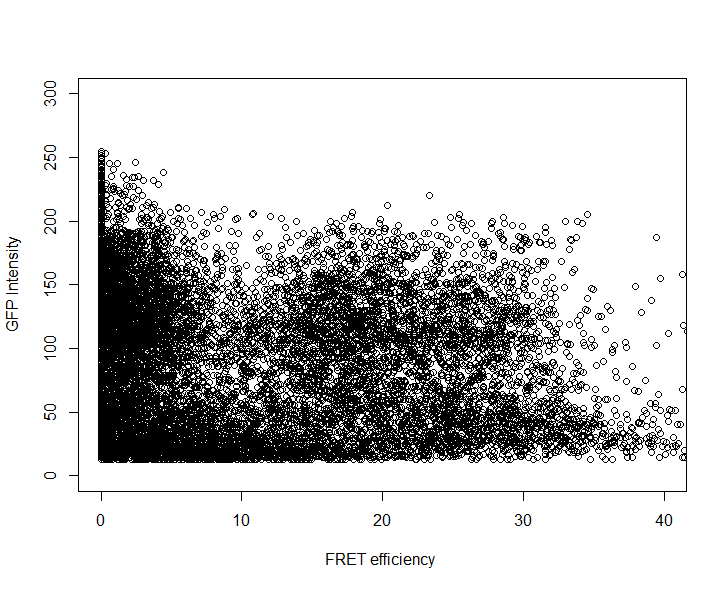

Supplement: Supplementary file 9 — Source Data for Figure 2 [file EMBJ-42-e110286-s004.zip › Figure 2/2A/Pearson correlation_graph.tiff]

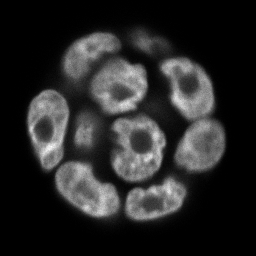

Supplement: Supplementary file 9 — Source Data for Figure 2 [file EMBJ-42-e110286-s004.zip › Figure 2/2B/ESCs_GFP intensity.tif]

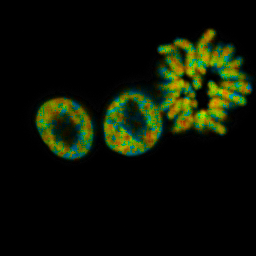

Supplement: Supplementary file 10 — Source Data for Figure 3 [file EMBJ-42-e110286-s009.zip › Figure 3/3D/BJ_H2B_2FPs_FRET efficiency_mitosis_PFA fixation.tif]

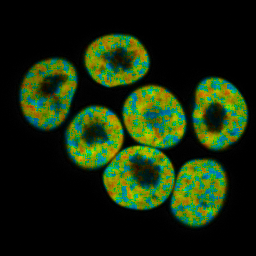

Supplement: Supplementary file 10 — Source Data for Figure 3 [file EMBJ-42-e110286-s009.zip › Figure 3/3D/BJ_H2B_2FPs_FRET efficiency_interphase_PFA fixation.tif]

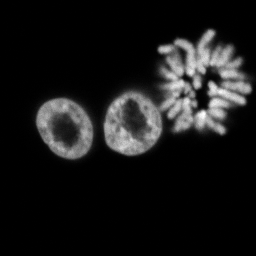

Supplement: Supplementary file 10 — Source Data for Figure 3 [file EMBJ-42-e110286-s009.zip › Figure 3/3D/BJ_H2B_2FPs_GFP intensity_mitosis_PFA fixation.tif]

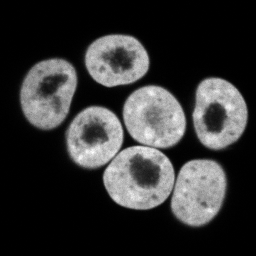

Supplement: Supplementary file 10 — Source Data for Figure 3 [file EMBJ-42-e110286-s009.zip › Figure 3/3D/BJ_H2B_2FPs_GFP intensity_interphase_PFA fixation.tif]

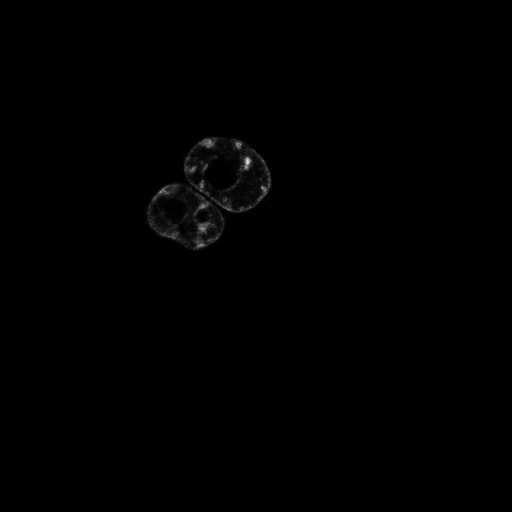

Supplement: Supplementary file 10 — Source Data for Figure 3 [file EMBJ-42-e110286-s009.zip › Figure 3/3A/BJ_2FPs_mtagBFP-HP1a_green channel.tif]

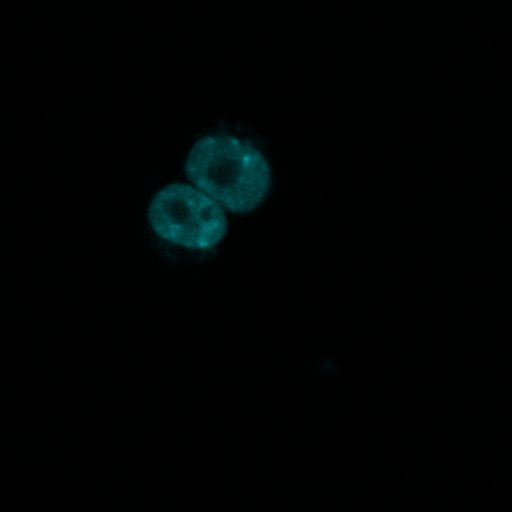

Supplement: Supplementary file 10 — Source Data for Figure 3 [file EMBJ-42-e110286-s009.zip › Figure 3/3A/BJ_2FPs_mTagBFP-HP1a_blue channel.tif]

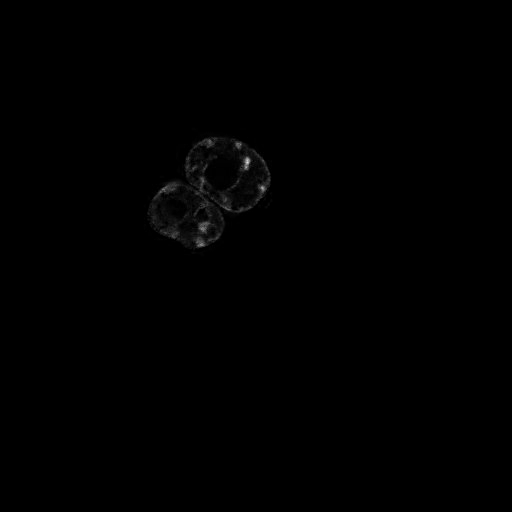

Supplement: Supplementary file 10 — Source Data for Figure 3 [file EMBJ-42-e110286-s009.zip › Figure 3/3A/BJ_2FPs_mtagBFP-HP1a_red channel.tif]

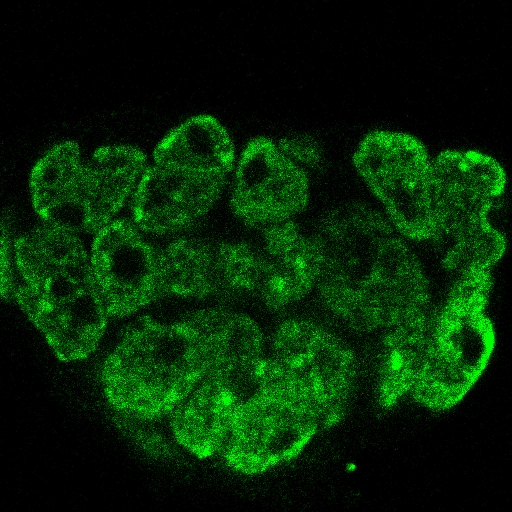

Supplement: Supplementary file 11 — Source Data for Figure 4 [file EMBJ-42-e110286-s011.zip › Figure 4/4E/BJ1 WT3_control_antiH4K20me3_Alexa647.tiff]

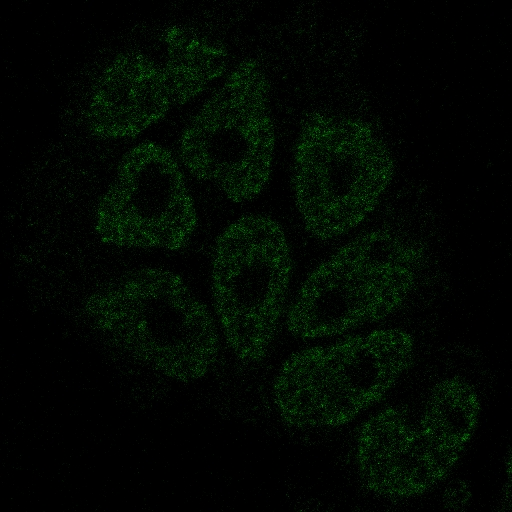

Supplement: Supplementary file 11 — Source Data for Figure 4 [file EMBJ-42-e110286-s011.zip › Figure 4/4E/BJ1 WT3_A196_antiH4K20me3_Alexa647.tiff]

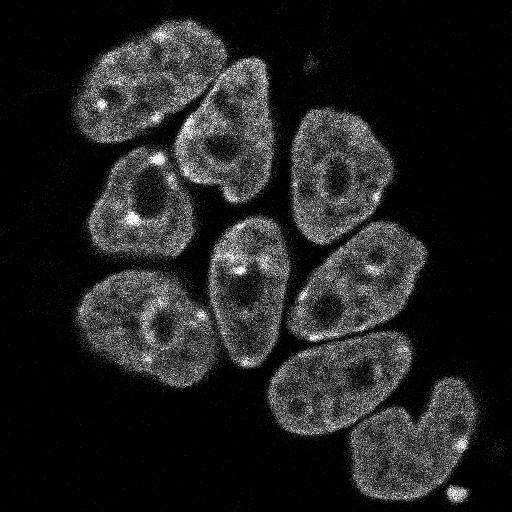

Supplement: Supplementary file 11 — Source Data for Figure 4 [file EMBJ-42-e110286-s011.zip › Figure 4/4E/BJ1 WT3_A196_antiH4K20me3_DAPI.tiff]

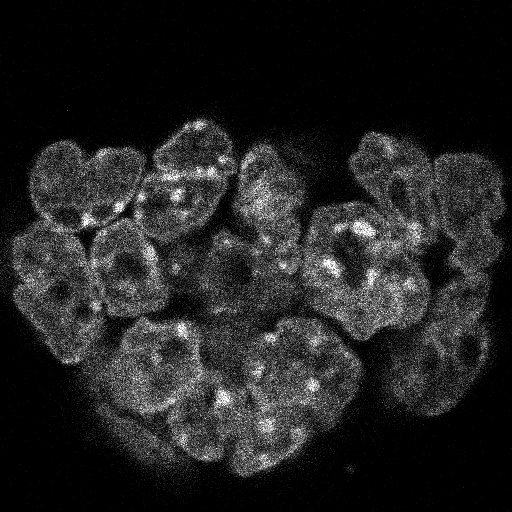

Supplement: Supplementary file 11 — Source Data for Figure 4 [file EMBJ-42-e110286-s011.zip › Figure 4/4E/BJ1 WT3_control_antiH4K20me3_DAPI.tiff]

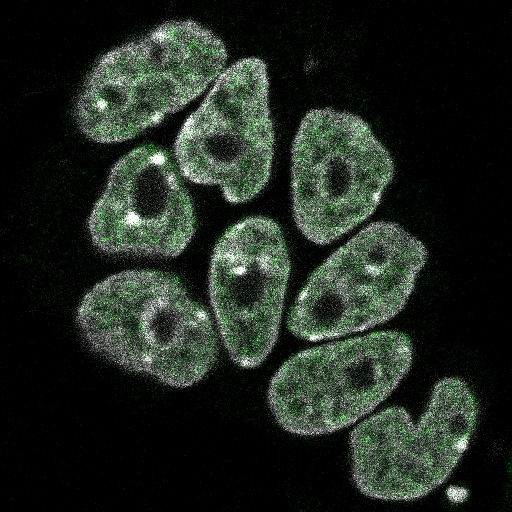

Supplement: Supplementary file 11 — Source Data for Figure 4 [file EMBJ-42-e110286-s011.zip › Figure 4/4E/BJ1 WT3_A196_antiH4K20me3_merge.tiff]

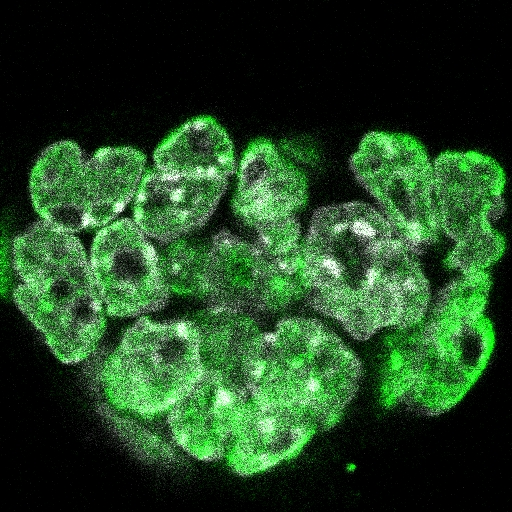

Supplement: Supplementary file 11 — Source Data for Figure 4 [file EMBJ-42-e110286-s011.zip › Figure 4/4E/BJ1 WT3_control_antiH4K20me3_merge.tiff]

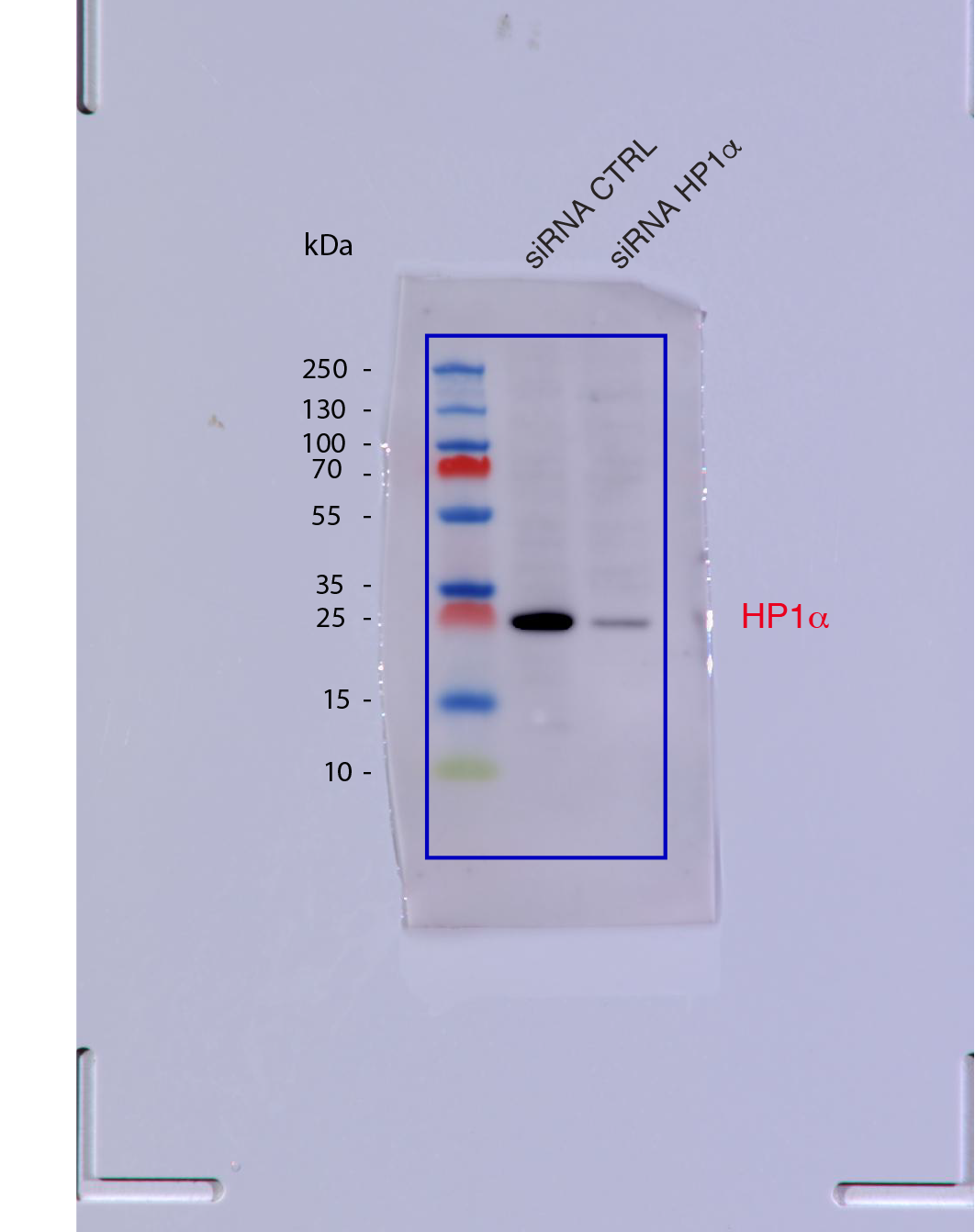

Supplement: Supplementary file 11 — Source Data for Figure 4 [file EMBJ-42-e110286-s011.zip › Figure 4/4A/Western_HP1a_siRNA CTRL_siRNA HP1a.tif]

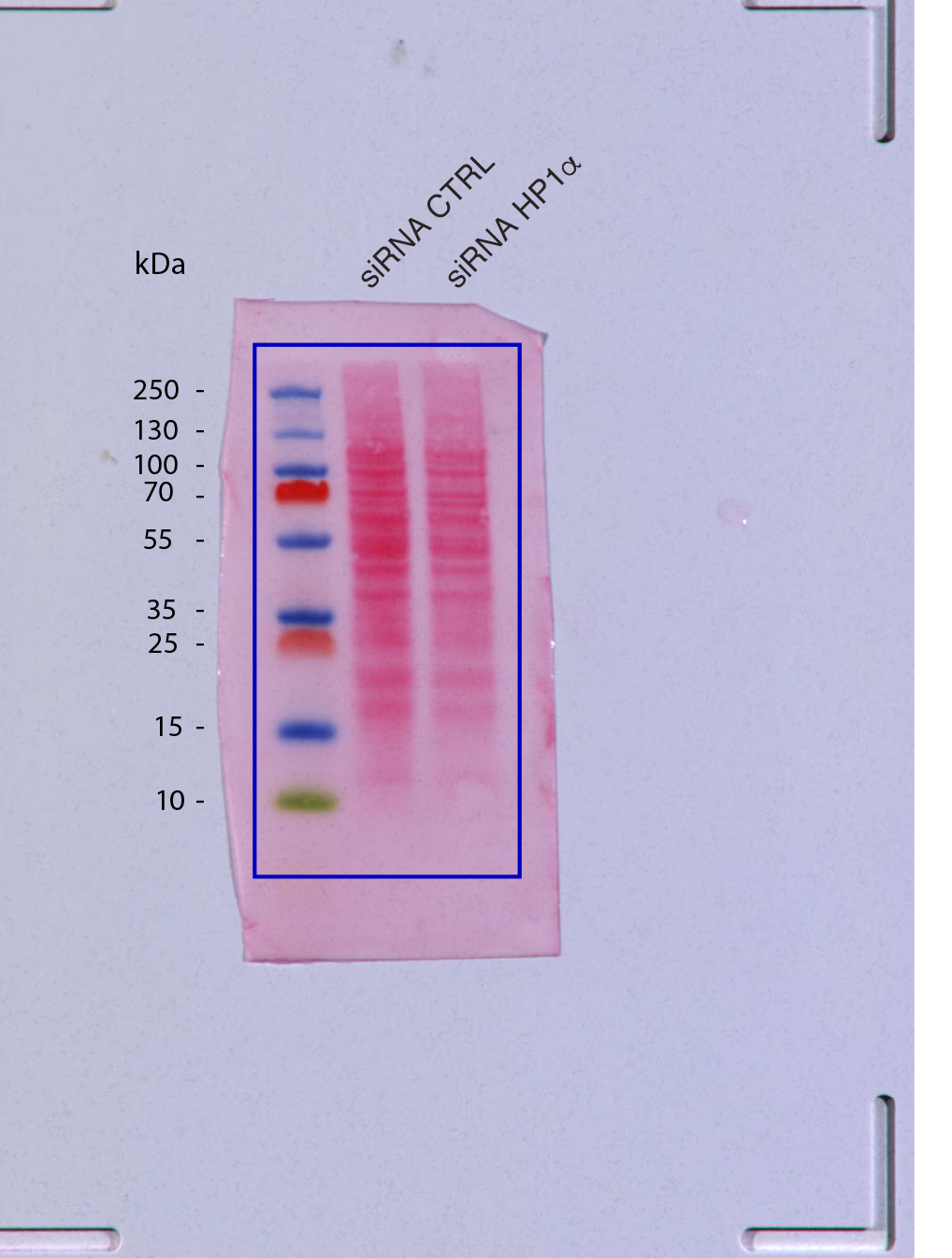

Supplement: Supplementary file 11 — Source Data for Figure 4 [file EMBJ-42-e110286-s011.zip › Figure 4/4A/Ponceau_siRNA CTRL_siRNA HP1a.tif]

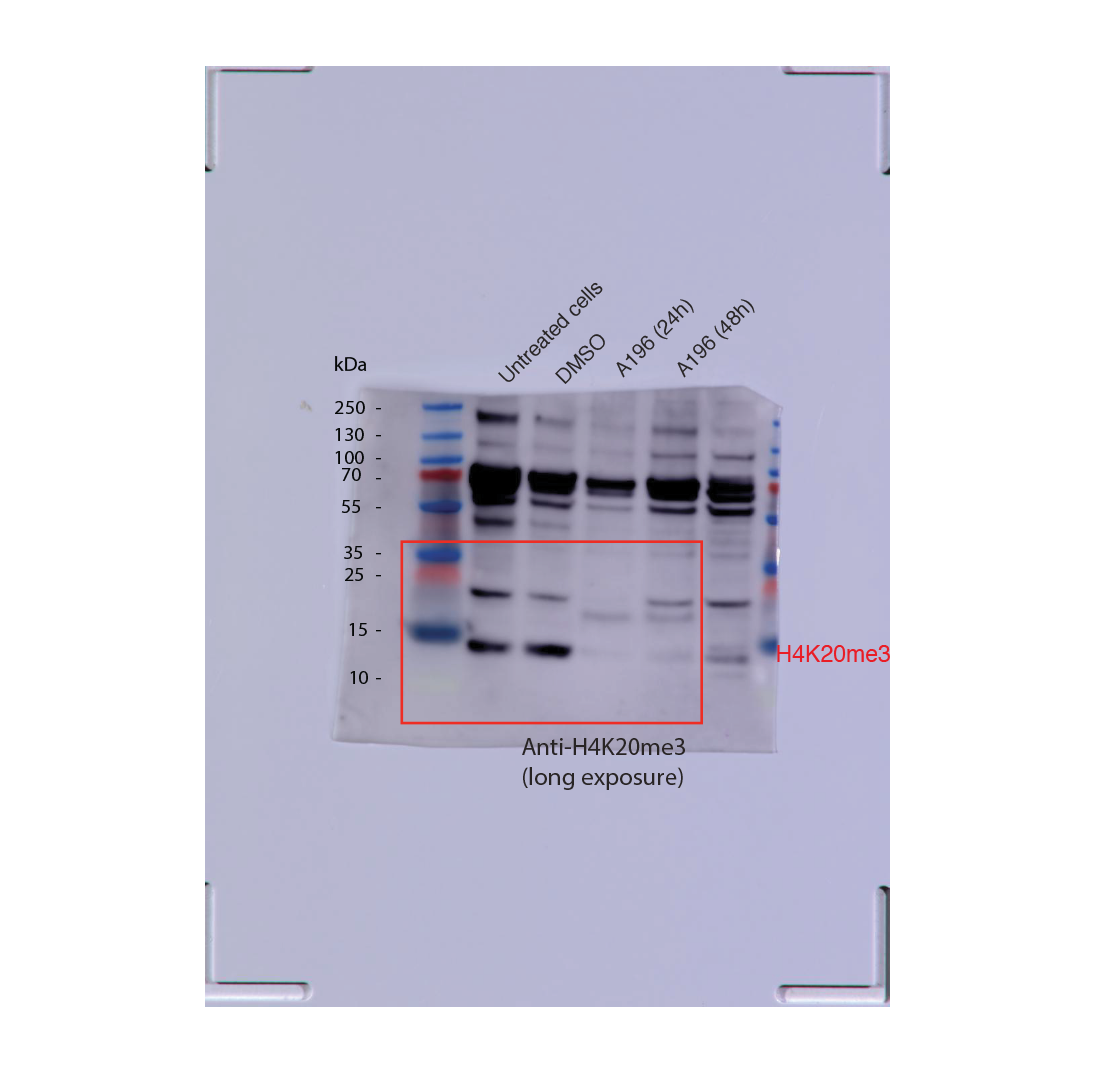

Supplement: Supplementary file 11 — Source Data for Figure 4 [file EMBJ-42-e110286-s011.zip › Figure 4/4F/Western_ H4K20me3_control vs A196.tif]

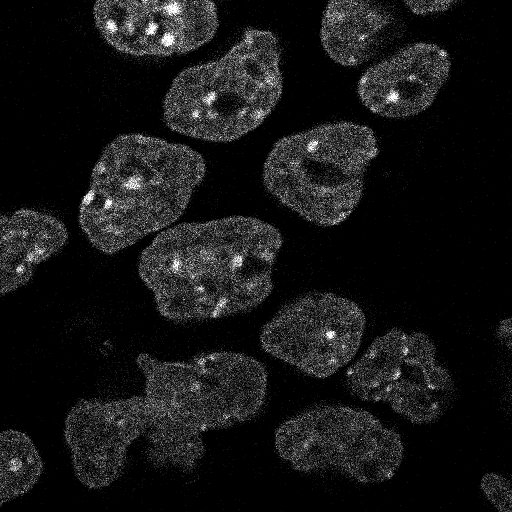

Supplement: Supplementary file 11 — Source Data for Figure 4 [file EMBJ-42-e110286-s011.zip › Figure 4/4G/BJ1 WT3_control_antiH4K20me2_DAPI.tiff]

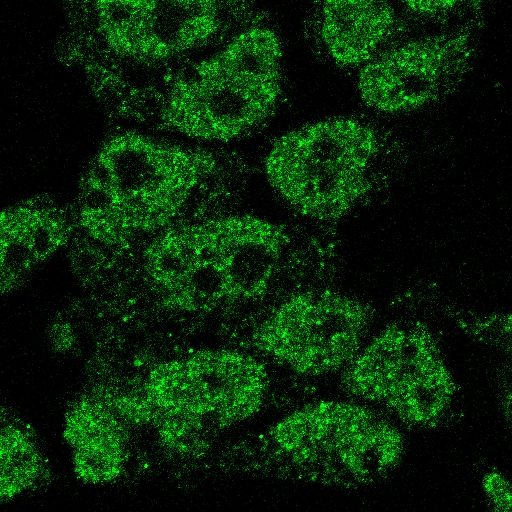

Supplement: Supplementary file 11 — Source Data for Figure 4 [file EMBJ-42-e110286-s011.zip › Figure 4/4G/BJ1 WT3_control_antiH4K20me2_Alexa633.tiff]

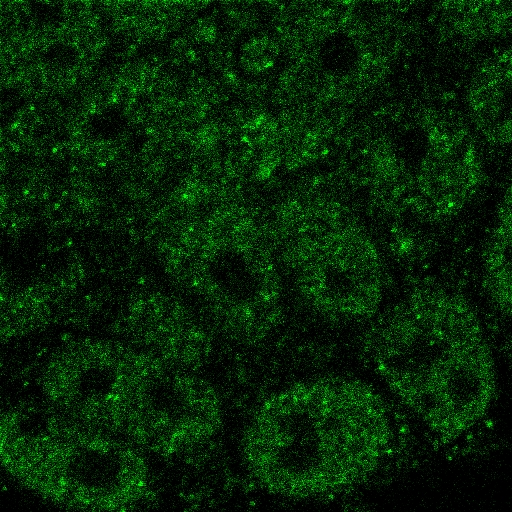

Supplement: Supplementary file 11 — Source Data for Figure 4 [file EMBJ-42-e110286-s011.zip › Figure 4/4G/BJ1 WT3_A196_antiH4K20me2_Alexa633.tiff]

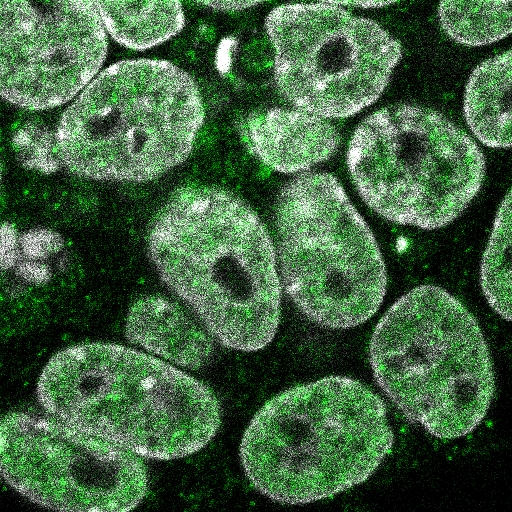

Supplement: Supplementary file 11 — Source Data for Figure 4 [file EMBJ-42-e110286-s011.zip › Figure 4/4G/BJ1 WT3_A196_antiH4K20me2_merge.tiff]

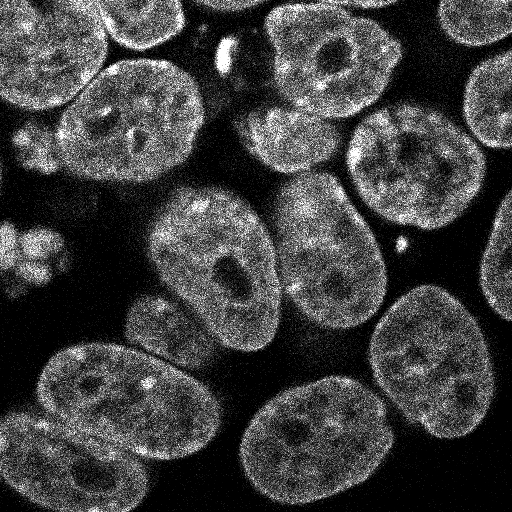

Supplement: Supplementary file 11 — Source Data for Figure 4 [file EMBJ-42-e110286-s011.zip › Figure 4/4G/BJ1 WT3_A196_antiH4K20me2_DAPI.tiff]

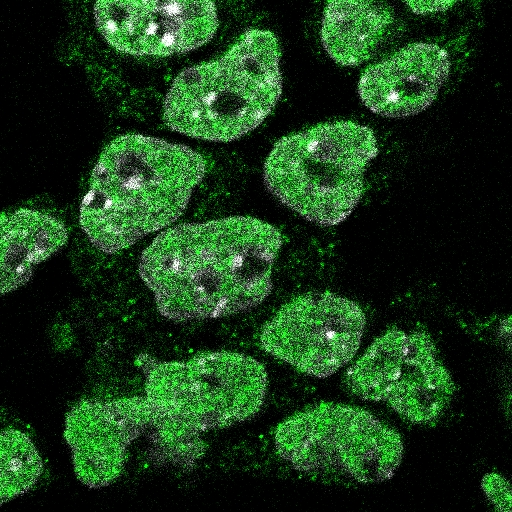

Supplement: Supplementary file 11 — Source Data for Figure 4 [file EMBJ-42-e110286-s011.zip › Figure 4/4G/BJ1 WT3_control_antiH4K20me2_merge.tiff]

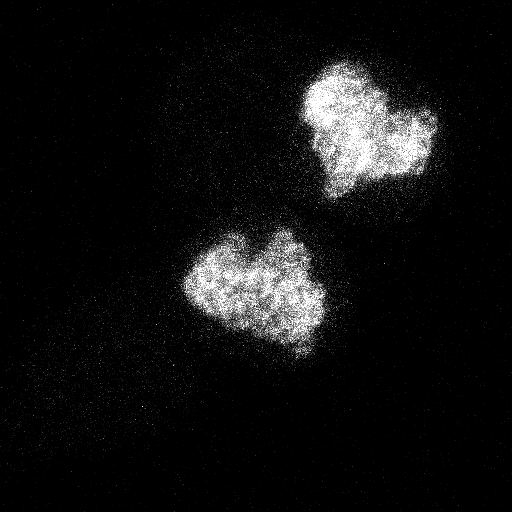

Supplement: Supplementary file 12 — Source Data for Figure 5 [file EMBJ-42-e110286-s006.zip › Figure 5/5A/BJ_H2B_2FPs_Control_anti Ki67_Alexa647.tiff]

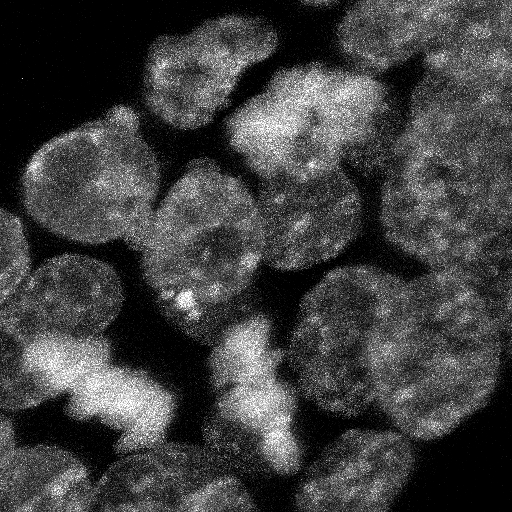

Supplement: Supplementary file 12 — Source Data for Figure 5 [file EMBJ-42-e110286-s006.zip › Figure 5/5A/BJ_H2B_2FPs_KO Ki67 #1_anti Ki67_DAPI.tiff]

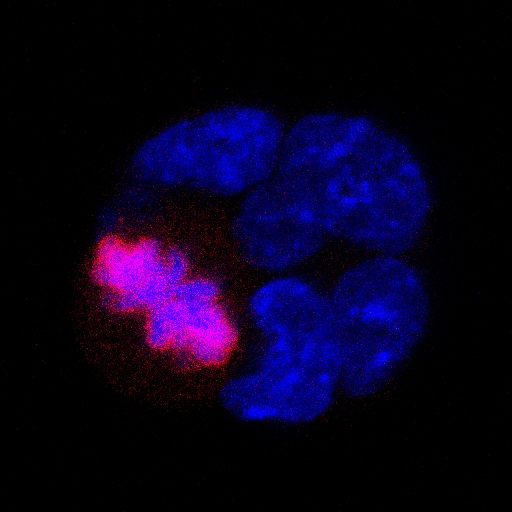

Supplement: Supplementary file 12 — Source Data for Figure 5 [file EMBJ-42-e110286-s006.zip › Figure 5/5A/BJ_WT_anti Ki67_merge.tiff]

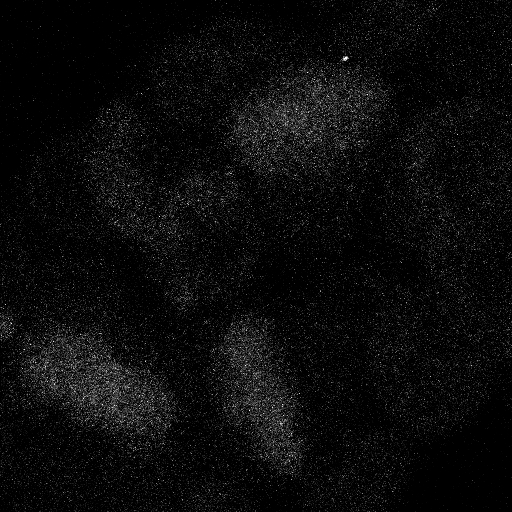

Supplement: Supplementary file 12 — Source Data for Figure 5 [file EMBJ-42-e110286-s006.zip › Figure 5/5A/BJ_H2B_2FPs_KO Ki67 #1_anti Ki67_Alexa647.tiff]

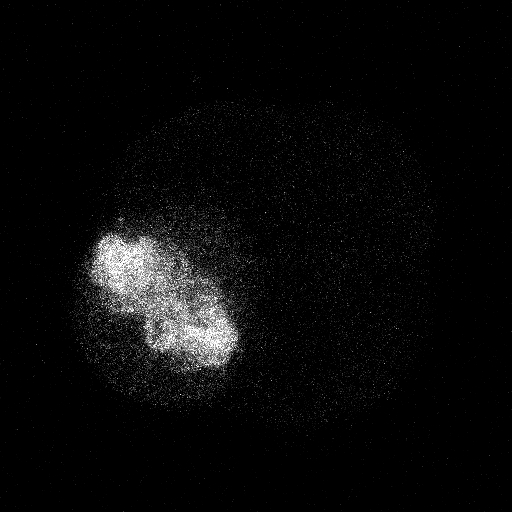

Supplement: Supplementary file 12 — Source Data for Figure 5 [file EMBJ-42-e110286-s006.zip › Figure 5/5A/BJ_WT_anti Ki67_Alexa647.tiff]

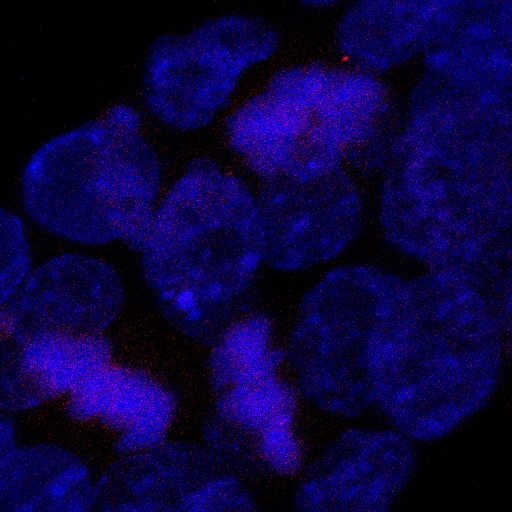

Supplement: Supplementary file 12 — Source Data for Figure 5 [file EMBJ-42-e110286-s006.zip › Figure 5/5A/BJ_H2B_2FPs_KO Ki67 #1_antiKi67_merge.tiff]

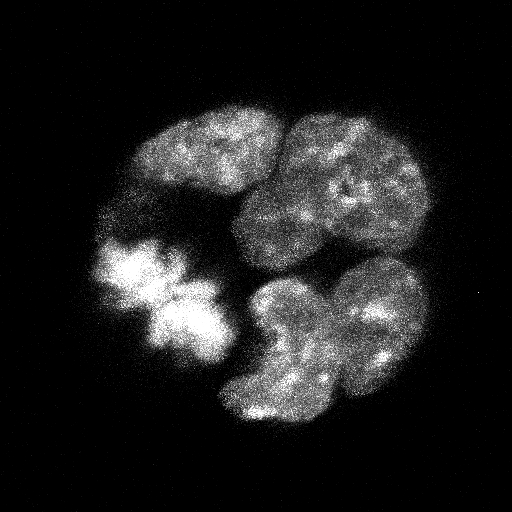

Supplement: Supplementary file 12 — Source Data for Figure 5 [file EMBJ-42-e110286-s006.zip › Figure 5/5A/BJ_WT_anti Ki67_DAPI.tiff]

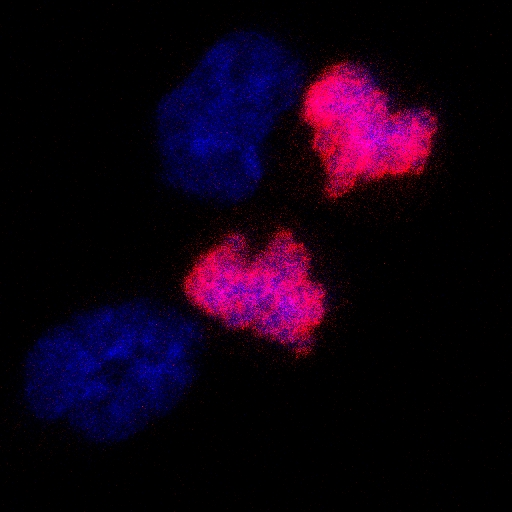

Supplement: Supplementary file 12 — Source Data for Figure 5 [file EMBJ-42-e110286-s006.zip › Figure 5/5A/BJ_H2B_2FPs_Control_anti Ki67_merge.tiff]

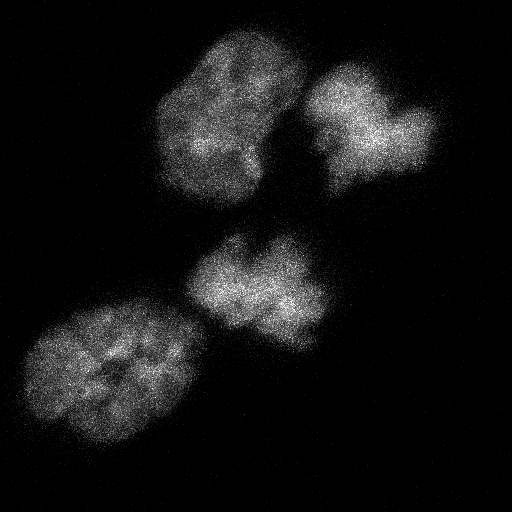

Supplement: Supplementary file 12 — Source Data for Figure 5 [file EMBJ-42-e110286-s006.zip › Figure 5/5A/BJ_H2B_2FPs_Control_anti Ki67_DAPI.tiff]

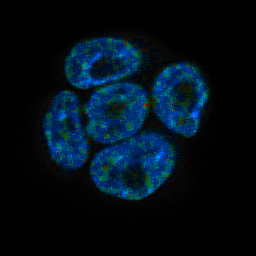

Supplement: Supplementary file 12 — Source Data for Figure 5 [file EMBJ-42-e110286-s006.zip › Figure 5/5C/BJ H2B_2FPs_Mki67 KO #1 clone_FRET efficiency.tif]

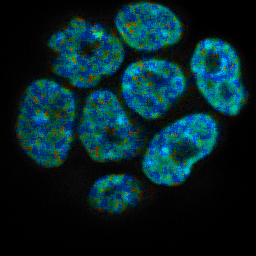

Supplement: Supplementary file 12 — Source Data for Figure 5 [file EMBJ-42-e110286-s006.zip › Figure 5/5C/BJ H2B_2FPs_Control_FRET efficiency.tif]

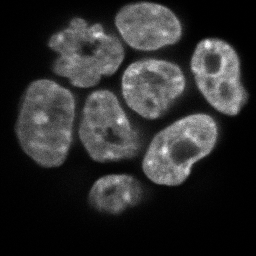

Supplement: Supplementary file 12 — Source Data for Figure 5 [file EMBJ-42-e110286-s006.zip › Figure 5/5C/BJ H2B_2FPs_Control_H2B_GFP intensity.tif]

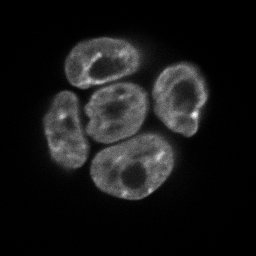

Supplement: Supplementary file 12 — Source Data for Figure 5 [file EMBJ-42-e110286-s006.zip › Figure 5/5C/BJ H2B_2FPs_Mki67 KO #1 clone_H2B_GFP intensity .tif]

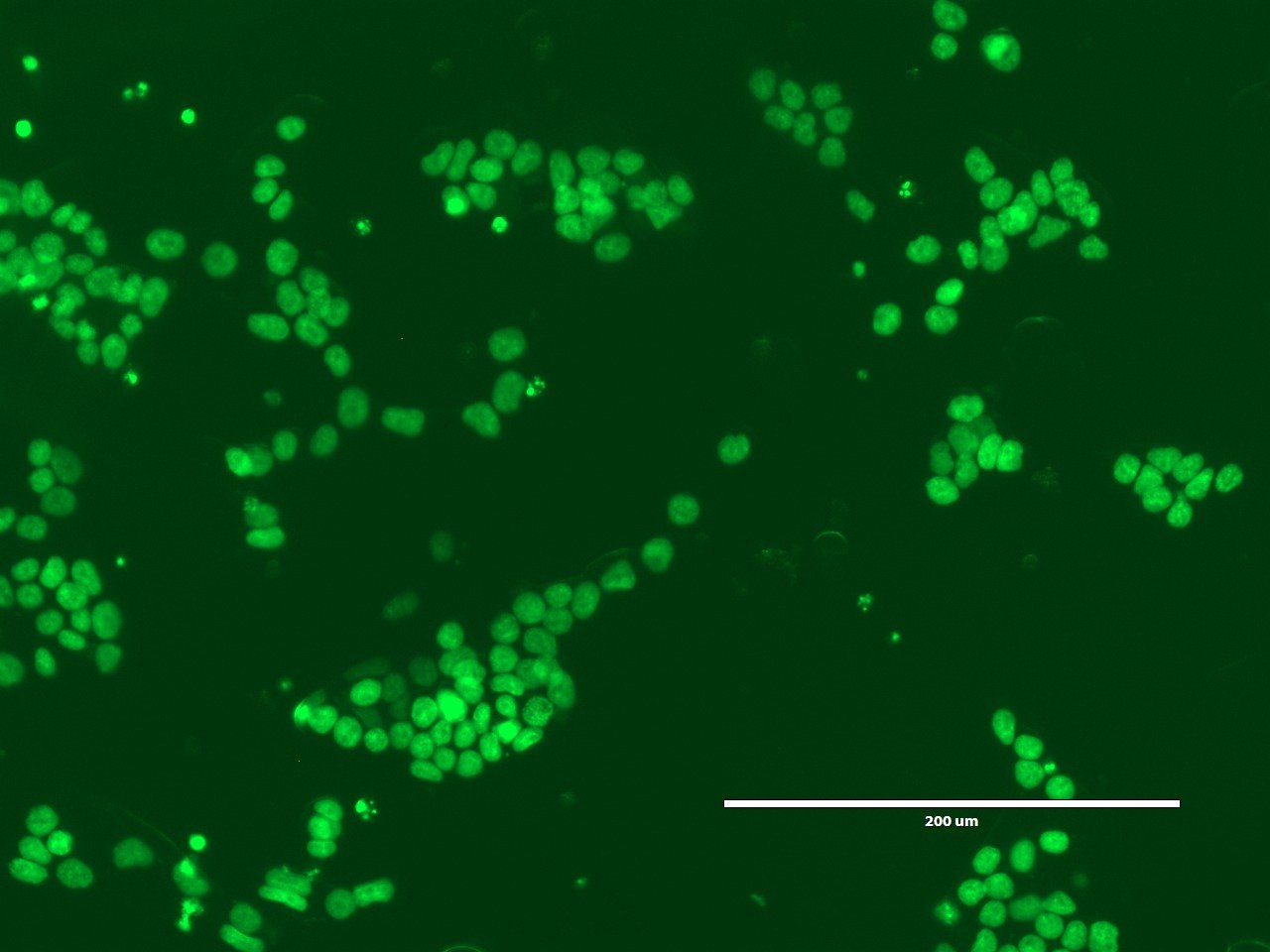

Supplement: Supplementary file 13 — Source Data for Figure 6 [file EMBJ-42-e110286-s014.zip › Figure 6/6A/EpiLCs_green channel.tif]

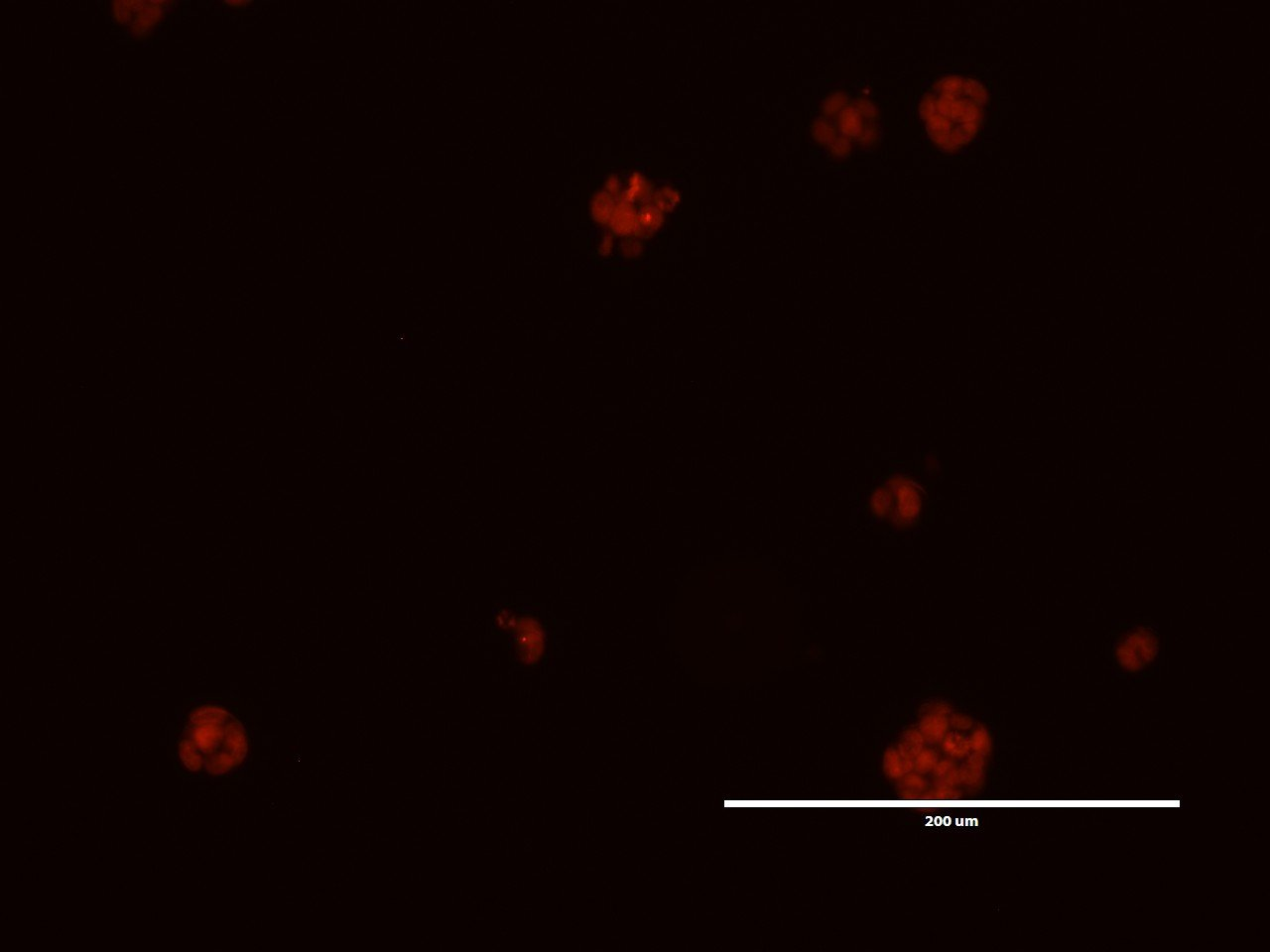

Supplement: Supplementary file 13 — Source Data for Figure 6 [file EMBJ-42-e110286-s014.zip › Figure 6/6A/ESCs_naive _mcherry channel.tif]

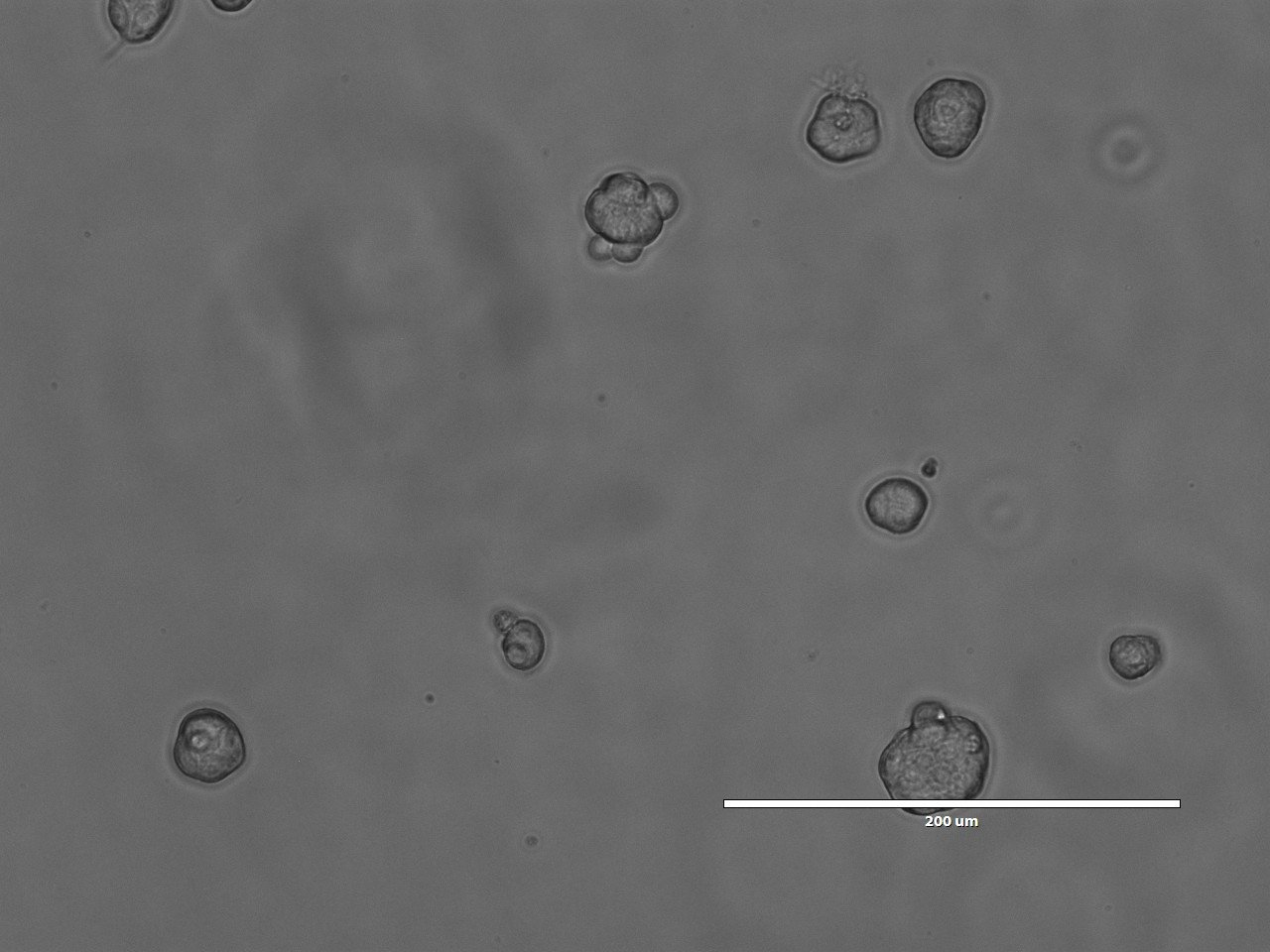

Supplement: Supplementary file 13 — Source Data for Figure 6 [file EMBJ-42-e110286-s014.zip › Figure 6/6A/ESCs_naive _phase contrast channel.tif]

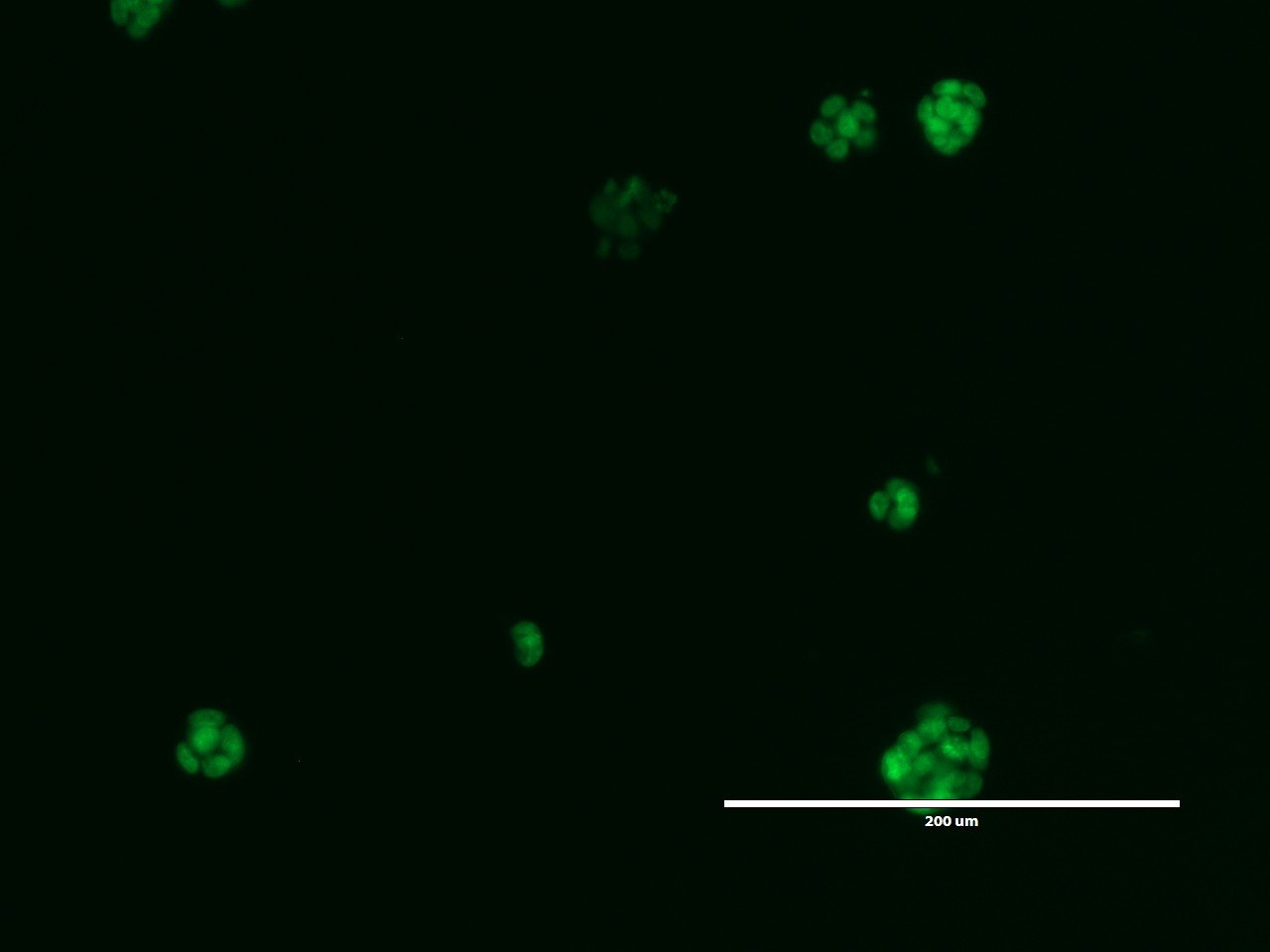

Supplement: Supplementary file 13 — Source Data for Figure 6 [file EMBJ-42-e110286-s014.zip › Figure 6/6A/ESCs_naive _green channel.tif]

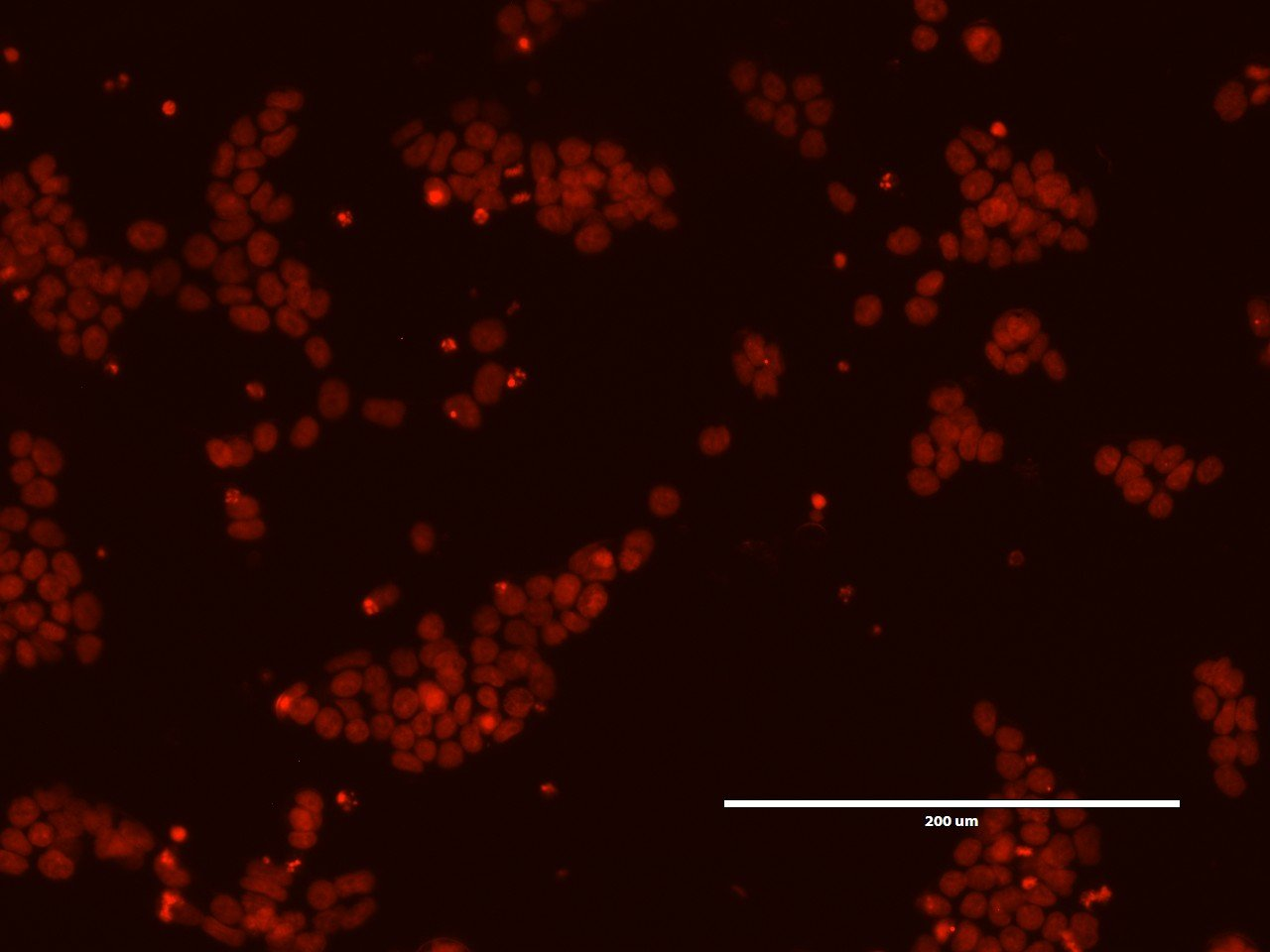

Supplement: Supplementary file 13 — Source Data for Figure 6 [file EMBJ-42-e110286-s014.zip › Figure 6/6A/EpiLCs_mcherry channel.tif]

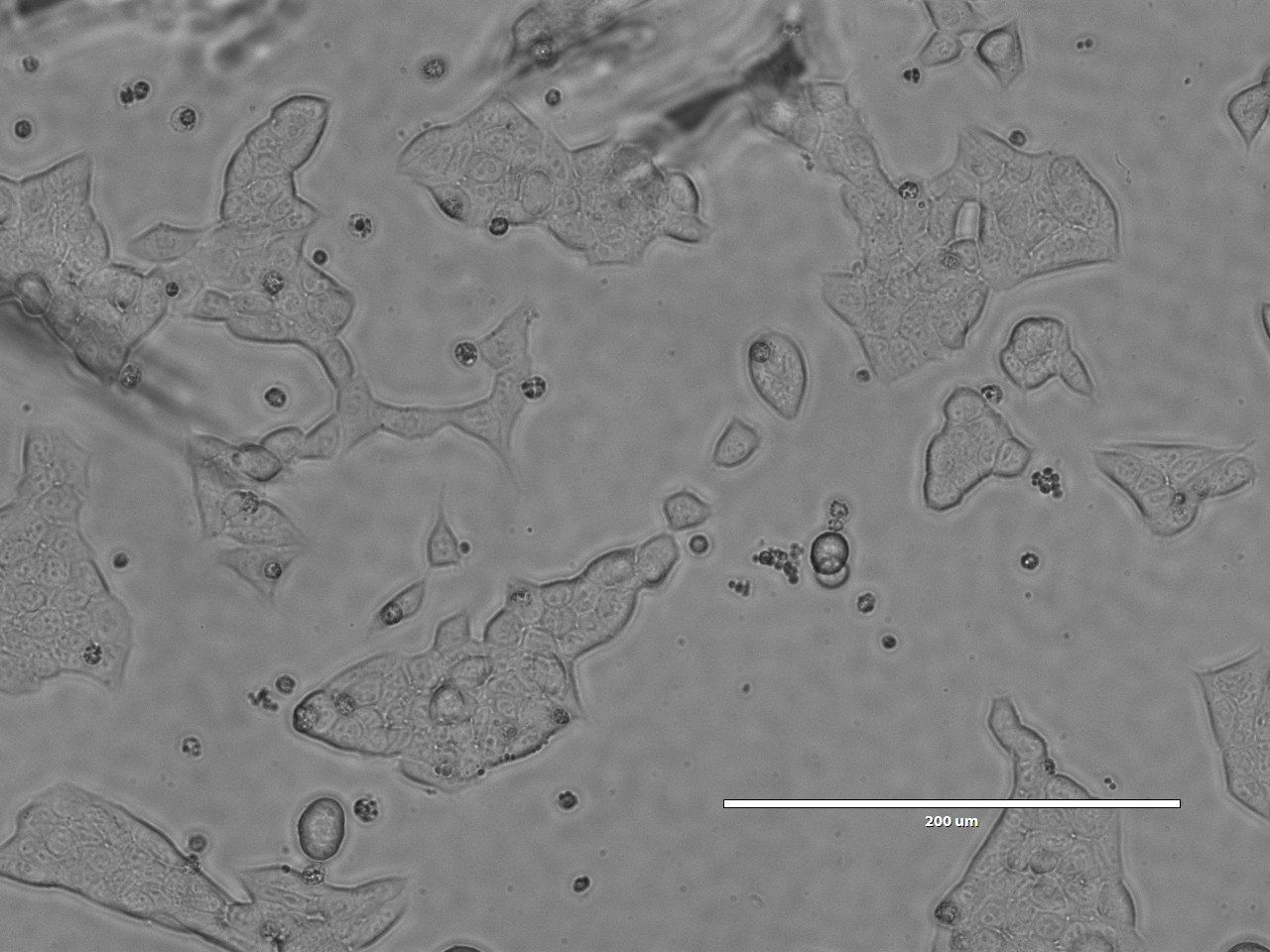

Supplement: Supplementary file 13 — Source Data for Figure 6 [file EMBJ-42-e110286-s014.zip › Figure 6/6A/EpiLCs_phase contrast channel.tif]

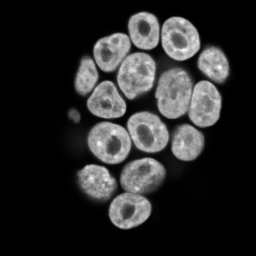

Supplement: Supplementary file 13 — Source Data for Figure 6 [file EMBJ-42-e110286-s014.zip › Figure 6/6B/ESCs_GFP intensity.tif]

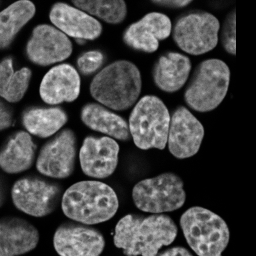

Supplement: Supplementary file 13 — Source Data for Figure 6 [file EMBJ-42-e110286-s014.zip › Figure 6/6B/EPILCs_GFP intensity.tif]

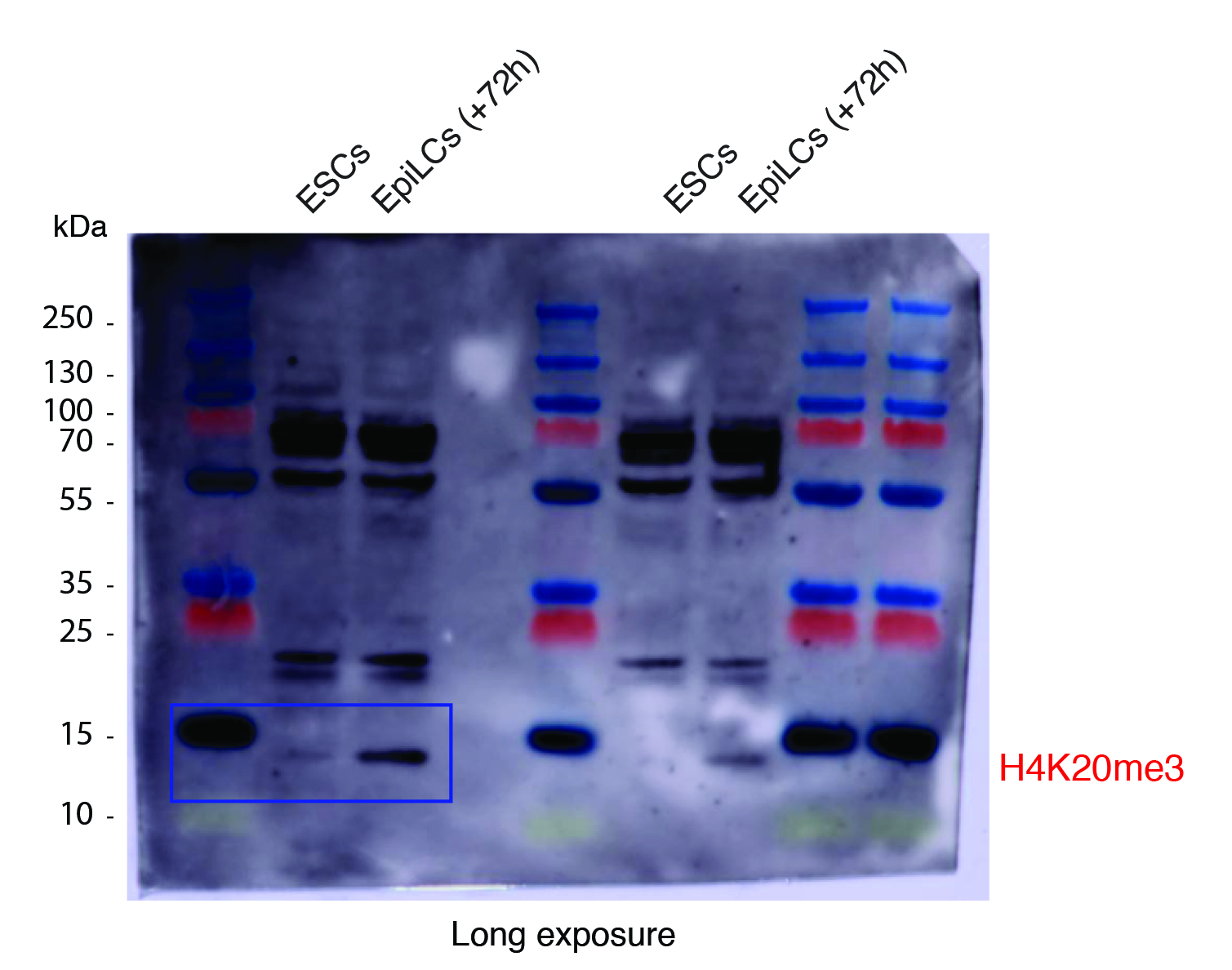

Supplement: Supplementary file 13 — Source Data for Figure 6 [file EMBJ-42-e110286-s014.zip › Figure 6/6E/Western H4K20me3_ESCs vs EpiLCs_long exposure.tif]

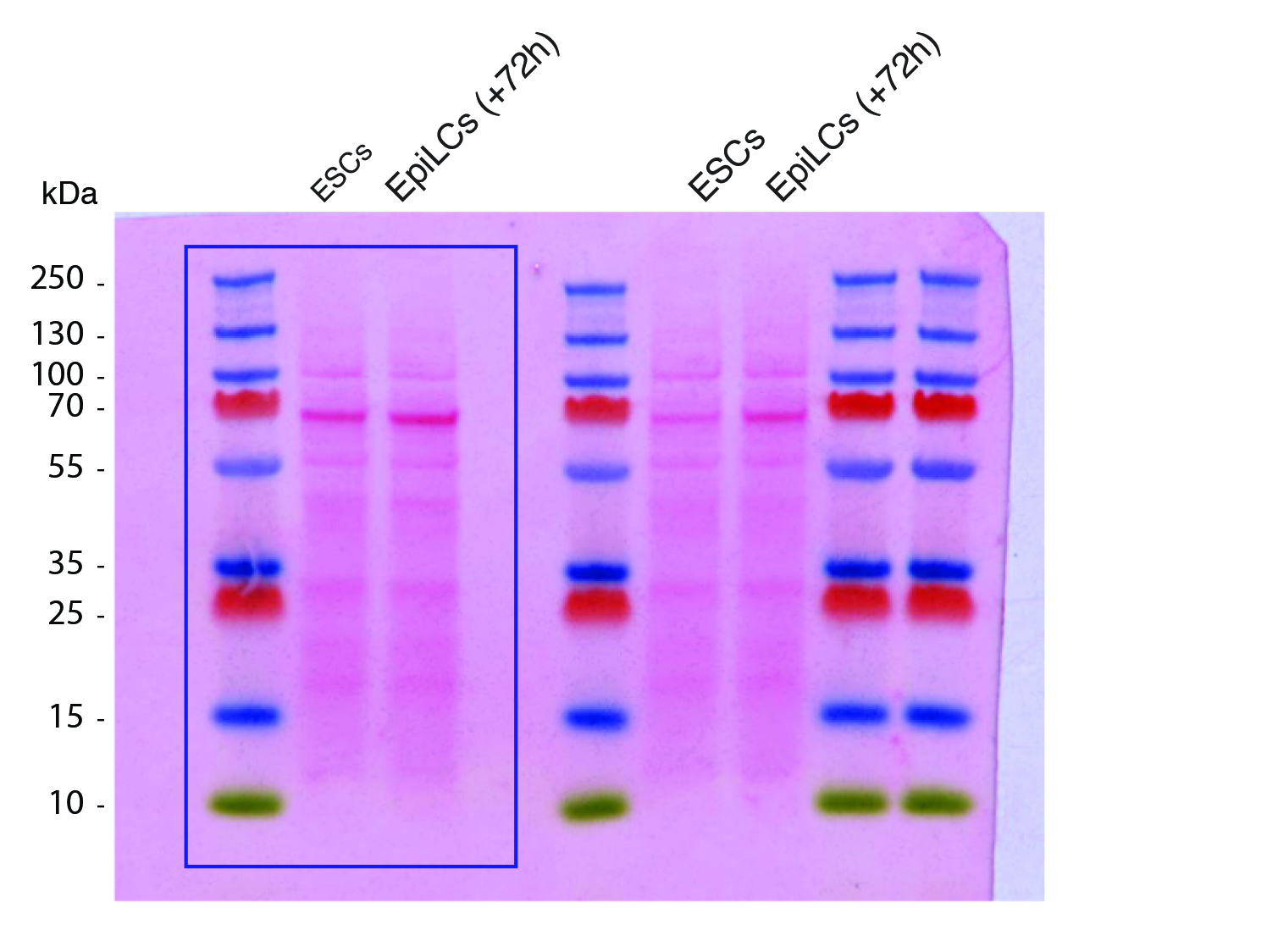

Supplement: Supplementary file 13 — Source Data for Figure 6 [file EMBJ-42-e110286-s014.zip › Figure 6/6E/Ponceau_staining_ESCs vs EpiLCs.tif]

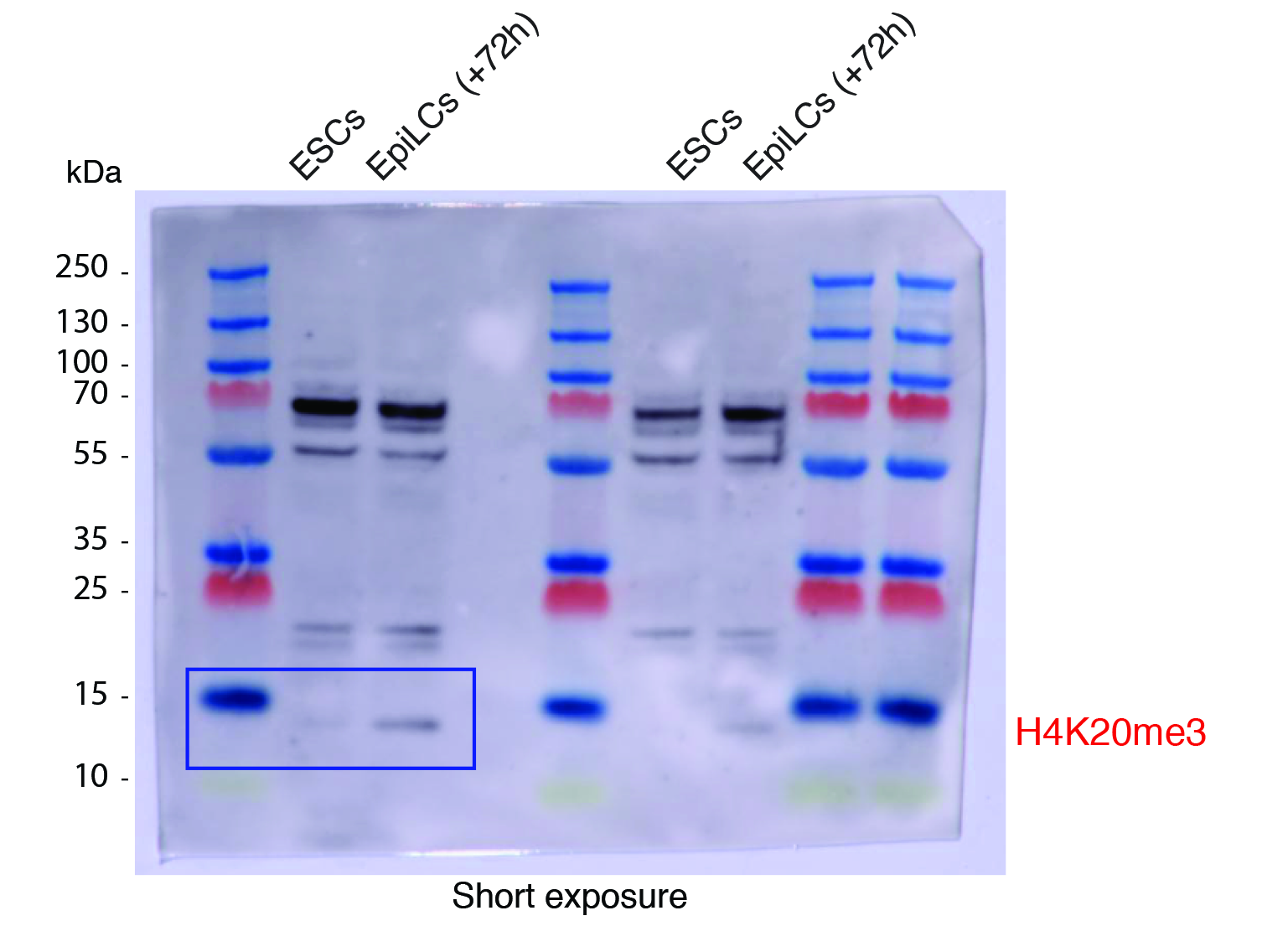

Supplement: Supplementary file 13 — Source Data for Figure 6 [file EMBJ-42-e110286-s014.zip › Figure 6/6E/Western H4K20me3_ESCs vs EpiLCs_short exposure.tif]
