# Supplementary material for: Evidence for low nanocompaction of heterochromatin in living embryonic stem cells
Source: EMBO J. 2023 Apr 21;42(12):e110286. doi: 10.15252/embj.2021110286 (PMC10267699; doi:10.15252/embj.2021110286)
Supplement: Supplementary file 14 [file EMBJ-42-e110286-s012.pdf]

Evidence for Low Nanocompaction of Heterochromatin in Living Embryonic Stem Cells

Claire DUPONT, Dhanvantri CHAHAR, Antonio TRULLO, Thierry Gostan, Caroline SURCIS, Charlotte GRIMAUD, Daniel Fisher, Robert Feil, and David Llères
DOI: 10.15252/embj.2021110286

Corresponding author(s): David Llères (david.lleres@igmm.cnrs.fr) , Robert Feil (robert.feil@igmm.cnrs.fr)

Review Timeline:

Submission Date:	30th Nov 21
Editorial Decision:	7th Jan 22
Appeal Received:	21st Dec 22
Editorial Decision:	31st Jan 23
Revision Received:	23rd Feb 23
Editorial Decision:	15th Mar 23
Revision Received:	22nd Mar 23
Accepted:	29th Mar 23

Editor: Hartmut Vodermaier / Stefanie Boehm

Transaction Report:

(Note: With the exception of the correction of typographical or spelling errors that could be a source of ambiguity, letters and reports are not edited. Depending on transfer agreements, referee reports obtained elsewhere may or may not be included in this compilation. Referee reports are anonymous unless the Referee chooses to sign their reports.)

1st Editorial Decision 7th Jan 2022

Dr. David Llères
Institute of Molecular Genetics of Montpellier (IGMM), CNRS, University of Montpellier
IGMM
Montpellier 34293
France

7th Jan 2022

Re: EMBOJ-2021-110286
Heterochromatin has low levels of nanocompaction in living embryonic stem cells

Dear Dr. Llères,

Thank you for submitting your manuscript proposing reporting a FLIM-FRET approach to analyze chromatin organization in living cells for consideration by The EMBO Journal. We have now received three referee reports on your study, which are included below for your information. Given the reviewer's comments, I am sorry to say that we have decided that we unfortunately cannot offer to invite a revision for EMBO Journal at this time.

As you will see, all reviewers acknowledge an interest in the reported findings and their potential implications for the understanding of heterochromatin. However, at this stage, the referees are not convinced that the experimental proof is sufficient to support the conclusions. Overall, they find that validation of the results acquired using the FLIM-FRET approach through the application of technically independent methodology would be required. Here the referees raise numerous specific issues that could cofound the interpretation of the current data and which would all have to be addressed and resolved in our view. Thus, while we recognize the overall potential interest, in our opinion, extensive additional work would be required to substantiate the conclusions in a manner that would make the study suited for publication in The EMBO Journal. Therefore, we unfortunately cannot invite a revision at this time. However, should you be able to address the crucial points in the future, we would be willing to reconsider a revised version of the manuscript. Please however note that in such cases, we again look at novelty and advance at the time of resubmission, and may also involve alternative or additional experts if needed.

I am sorry that I cannot be more positive on this occasion, but hope that you will nonetheless find the comments of our reviewers helpful. Thank you again for giving us the opportunity to consider your manuscript.

Kind regards,

Stefanie Boehm

Stefanie Boehm
Editor
The EMBO Journal

****

Referee #1:

The authors analyze the compaction of constitutive heterochromatin in ESCs and EpiLCs cells at nanometer resolution using the system FILM-FRET, in which two fluorescent proteins are expressed as histone fusion proteins. The authors have also defined the term 'nanocompaction', which stands for the spatial organization of nanometer-range proximity between nucleosomes. Interestingly, they showed that there is no correlation between chromatin-dense regions and the regions where FRET is observed. They also showed that the low FRET state in chromatin-dense regions is maintained in an ATP-dependent manner. In addition, the effects of knockdown of the HP1 subtype and inhibition of H4K20me3 on nanocompaction are analyzed. In conclusion, the authors suggested that heterochromatin is not highly condensed as shown by FRET in living cells.
This is very provocative and different from the traditional heterochromatin state, which has been discussed mainly in fixed cells.

The results of this study are as surprising as they are interesting. The considerable amount of material and variety of experimental tools and methods used to analyze deacetylation, methylation, the effect of HP1 protein, and nanocompaction in naive and primary stem cells level are highly notable. However, there are several major difficulties with this manuscript which make it unsuitable for publication in EMBO J.

Major comments:

1. The major concern of this manuscript is the fact that all findings depend on FLIM-FRET results. There is a risk that the interpretation of the results obtained may be influenced by the accuracy of FLIM-FRET. The authors should therefore take into consideration of other factors that could affect FRET efficiency, other than the 'nanocompaction' level. For example, the FRET efficiency may be a combined result of multiple factors such as chromatin rigidity, phase separation level, and chromatin compaction. In heterochromatin regions, the rigidity of chromatin itself and/or the phase separation level may cause the decrease of FRET efficiency even if two labeled nucleosomes are close to each other spatially. It is also quite concerning to discuss about phenomenon occurring at 10 nm based on the 890 nm intensity of FRET, which has a long wavelength and low resolution. Other experimental methods should be applied to confirm the findings in this paper.
In addition, it might be expected that there is a mechanism in heterochromatin that prevents the fluorescent proteins used in this study from condensing with each other. To rule out this possibility, the authors should conduct experiments using fluorophores other than GFP and mCherry or change the type of histone to which the fluorescent proteins are bound.

2. In this study, the authors found that heterochromatin shows low level of nanocompaction using FLIM-FRET method, even when enriched in H2B-GFP. This observation is very different from the heterochromatin state observed in fixed cells from previous reports. The authors should discuss why they obtain such surprising results, that are different from the results observed in the fixed cells. In addition, the authors should show whether the efficiency of H2B-GFP and mCherry-H2B incorporation into chromatin is the same in all chromatin regions.

3. The correlation between the nanocompacted chromatin state, as defined by the authors, and the function of nanocompaction is unclear. Low FRET signal is observed in chromatin-dense regions while high FRET signal is observed in non-chromatin-dense regions. The authors should identify the chromatin regions where high FRET is observed and clarify the correlation between the chromatin structure and function of that region and nanocompaction.

4. In the region where nanocompaction is happening, FRET occurs, although H2B-GFP fluorescence is not visible. However, since the DNA content is not shown in living cells, the specific area where nanocompaction occurs remains unclear. Therefore, the authors should stain DNA. Since H2B-GFP fluorescence co-localizes with DAPI, Hoechst and DRAQ5 can be used instead to stain living cells.

5. Page 5, Line 90-93: The authors cited "Norton et al. 2008" as a previous heterochromatin study, but this paper focuses mainly on perinucleolar compartments. Proper citation is thus required.

6. The authors evaluated the effect of ATP depletion on nanocompaction and observed a loss of activity of ATP-dependent chromatin remodeling factors. First, such an interpretation is difficult, although high FRET signals were observed in chromatin-dense regions, because ATP depletion deeply impacts the cells, such as the shutdown of all phosphatases and enzymes involved in ubiquitination. Therefore, the authors should further investigate the cause of the observed nanocompaction change by performing knockdown analyses of ATP-dependent chromatin remodeling factors.
Secondly, the authors cited "Iurlaro et al., 2021, Schick et al., 2021" as references when they mentioned that "ATP depletion leads to loss of activity of ATP-dependent chromatin remodeling complexes" (Page 8, Line 170-171). These studies are not related to ATP-depletion but are focused on the intracellular function of the ATPase subunits of a remodeler, by using reagents that specifically inhibit the ATPase activity, or the method targeted degradation, respectively. Therefore, the authors should cite more appropriate references.

7. The authors concluded that HP1α and HP1β have different effects on nanocompaction level. However, the authors only observed HP1α-depleted cells and HP1α/HP1β-depleted cells, but not HP1β-depleted cells. Since it has been reported that knockdown of HP1 subtype changes the expression of other subtypes, it is necessary to conduct knockdown experiments of HP1β alone to analyze the effects of HP1α and HP1β on nanocompaction.

Minor comments:

1.Page 2, Line 32: nanocompaction should be one word.
2. Page 14, Line 320: "Expanded View Fig 4D" should be "Expanded View Fig 4E".
3. In the Figures, Figure1 f and g are in lower case.
4. In the Results section, BJH2B-2FP cells is written in one word throughout the text, however in the Figures and the Figure legends, it is written as BJ H2B-2FP cells. Please unify.
5. Page 14. Line 317, KMT: please indicate the meaning of the abbreviation, such as lysine methyltransferase.
6. Page 15, Line 350: (Fig. 5d), d is in lower case.
7. Page 21, Line 494: "These including" should read "These include".
8. The term "naïve" is spelled differently throughout the text. Please unify.

Referee #2:

This paper reports on reduced FRET in living cell heterochromatin that has incorporated histone H2B-EGFP and H2B-mCherry. The authors show that fixed cells have increased FRET and argue that fixation is responsible for the observed dense organization of heterochromatin. The data most certainly is intriguing and may be consistent with the freedom of movement of chromatin within heterochromatin domains. However, there is significant evidence that nucleosomes within dense chromatin undergo less diffusion than nucleosomes in euchromatic regions of the genome. While not direct evidence, this suggests increased crosslinking of chromatin fibers within heterochromatin domains. Moreover, the observed fluorescence intensity of both the mCherry and the EGFP tagged histone H2B demonstrate increased chromatin densities wthin the heterochromatic regions of the genome. The authors misinterpret the data on accessibility, which clearly shows reduced access of freely diffusing nuclear probes to the interior of heterochromatin is restricted at higher molecular weights while accessibility is increased in the presence of histone deacetylase inhibitors (PMID: 16317046). Moreover, they ignore FLIM experiments that show changes in fluorescence lifetime that correlates with condensation state (PMID: 26765322). Most notable is the application of the same method of H2B FRET in NIH-3T3 cells (PMID: 29535103 Figure 2C). In this timelapse experiment, we can see a decrease in heterochromatin FLIM over time. One explanation for this would be the accumulation of DNA damage during imaging, driving decondensation. Finally, their own previous and cited C. elegans study (PMID: 28199849) obtains the opposite result-HP1 proteins promote condensation. These studies contradict the conclusions of this paper and, hence, require that we scrutinize the interpretation of the results of the current study.
Major concerns:
1) Given the concerns about DNA damage, there is a requirement to assess whether or not these cells are experiencing DNA damage. In particular, PARP activation might explain the decreased chromatin density in heterochromatin. Treatment with PARP inhibitors prior to imaging and staining of the cells for PAR accumulation after imaging (fixation and staining post live cell imaging) would address these concerns. Note that PAR has a short lifetime and may be difficult to detect if fixation is delayed significantly following imaging.
2) What the authors directly demonstrate is that FRET increases upon fixation. The relative FRET observed in a previous similar study did not reveal decreased density in heterochromatin. They claim that the nuclear volume is maintained under these conditions. In our hands, there is condensation but it is accompanied by a decrease in nuclear volume. The authors could validate this effect by placing cells in hypertonic media, which will generate the same phenomenon. An alternative explanation is that the heterochromatin undergoes more nucleosome-nucleosome interactions (reduced nucleosome spacing) in the absence of fixation than the surrounding chromatin but the incorporation of the histones is reduced such that the FRET efficiency is lower. Fixation will still generate increased FRET signal but neither can be directly compared to regions outside of the heterochromatin because of differences in the density of FP-H2B incorporation.
A more direct test of the relative incorporation of the fluorescent proteins into chromatin is required. I would suggest that the EGFP be used for ChIP experiments to demonstrate that representative active, facultative heterochromatin (repressed tissue specific genes repressed by polycomb), and satellite sequences associated with constitutive heterochromatin. If the histone is evenly distributed, this experiments should reveal no significant enrichment or depletion of any of these classes of sequences. Alternatively, ChIP-seq experiments could be done. Again, the expectation is a very homogeneous distribution.
Ultimately, the claim of fixation-dependent changes in chromatin organization is difficult to evaluate in the absence of demonstrating the same phenomena in a more commonly studied cell line where the chromatin state is better characterized. Most suitable for this study would be the use of NIH 3T3 cells, where there is existing live cell FLIM data using this H2B system (PMID: 29535103).
The results on K20 methylation are interesting in their own right. However, the distinction between di and trimethylation of K20 is important. Dimethylation is very abundant and found throughout the genome whereas the trimethylation is much more restricted to heterochromatin. These experiments and the related HP1 experiments would be nicely complemented by the addition of suv39h1/h2 knockout to eliminate the K9 trimethylation, HP1, and downstream K20 trimethylation specifically in constitutive heterochromatin.

Referee #3:

Constitutive heterochromatin, which is mainly formed at the pericentromeric regions, is thought to be condensed and transcriptionally silenced. The formation relies on the recruitment of HP1, which can crosslink nucleosomes containing histone H3K9me3 and promote chromatin compaction. However, the physical state of heterochromatin in living cells is not fully understood. Using the FLIM-FRET between nucleosomes, where H2B-EGFP and mCherry-H2B were incorporated, Dupont et al. measured chromatin compaction in living mESCs. The authors found that the FRET efficiency at the chromocenter heterochromatin in living cells is lower than in other regions. In contrast, in formaldehyde-fixed cells, the FRET efficiency of heterochromatin increased. Based on this data, they claimed that the heterochromatin at the chromocenter is less condensed in living cells. The authors showed that HP1a knockdown increased FRET efficiency in living ESCs, but this effect was overridden by loss of HP1b. Furthermore, depletion of H4K20me3 decreased the FRET efficiency. Finally, knockout of Ki-67 strongly reduced the FRET efficiency in interphase and mitotic heterochromatin in ESCs. The obtained findings are at odds with the prevailing models and intriguing and thought-provoking in the chromatin-related fields. However, there are several points to be addressed for publication in The EMBO journal. My specific comments and suggestions are the following:

Major comments:
1) Line 204 (Fig. 2A). "we observed no correlation between fluorescence intensity levels (i.e., H2B-GFP density) and FRET

efficiency (Pearson coefficient r =-0.07, n=106 cells)". Although this is a really exciting point, it is possible that FRET efficiency is not necessarily related to chromatin compaction. H2B-GFP intensity in confocal microscopy imaging of living cells should be related to the nucleosome density (chromatin compaction) if there is no bias for H2B-GFP-incorporation in the genome. There are a number of such data in the cited references. I wonder whether FRET efficiency could be affected by not only distances between H2B-GFP and mCherry-H2B, but also other factors in the chromatin environment, such as relative angles of the fluorescent probes, associated protein (e.g., HP1a), and so forth. If the chromatin-associated HP1a somehow disturbs the FRET efficiency in living cells, the authors' whole story may collapse. Note that the binding mode of HP1a also can be changed in the formaldehyde-fixed cells. The authors should address this issue.

2) "Constitutive heterochromatin shows low nanocompaction in living ESCs." I wonder whether the low FRET efficiency in the constitutive heterochromatin is unique in living ESCs or not. It is essential to know whether it is true for somatic mouse cells, which have chromocenters. For instance, the immortalized and differentiated cell line such as NIH3T3?

3) Line 307-309. "upon siRNA-mediated HP1α/b co-depletion in BJH2B-2FPs ESCs..."
The authors may want to know whether HP1β-single depletion reduces FRET efficiency to the same extent as HP1α/β co-depletion.

4) Line 456-458. "Our data on living cells suggest that ..."
Could authors explain how the more plastic and dynamic nature of heterochromatin contributes to some cellular functions such as transcriptional silencing?

5) Line 185-186. "TSA treatment strongly increased global histone tail acetylation ..." The difference is not so prominent. The senior author previously performed a similar experiment with TSA-treated HeLa cells (Llères et l., JCB 2009, Figure 5B), and found a clear difference between untreated and TSA-treated cells. This point should be clarified.

Minor comments:
1) Why did the authors use H2B-GFP and mCherry-H2B for FILM-FRET, but not H2B-mCherry?

2) Line 384- 386. "In addition, we noticed that two different populations of FRET efficiencies ..."
I am not sure if two different populations of FRET efficiencies were observed only in EpiLCs. Even in mESCs (CTRL) of Fig. 5D, the FRET efficiency distribution seems to have two different populations. Why?

3) There are typos in the manuscript as described below.
Figure 1. "Figure 1f" and "Figure 1g" -> "Figure 1F" and "Figure 1G".
Line 320. "Expanded View Fig 4D" -> "Expanded View Fig 4E".
Line 350. "Fig. 5d" -> "Fig. 5D".

4) Line 481. "Because of" -> "Because"?
Line 511. "the interplay of these components in and..." -> "the interplay of these components and..."?

Author Rebuttal to the initial comments

We thank the three reviewers for their constructive comments and helpful suggestions on how to improve our study. The revised manuscript addresses the points raised by the reviewers, with addition of new experiments, additional data analyses and additional explanatory texts. Please note, that new and modified sentences are marked in blue in the revised manuscript.

In summary, these are the main changes to the manuscript:

1. We now provide additional structural data, using a second, technically independent method that assesses changes in chromatin density as a readout of chromatin compaction (new **Fig. S1E and S4B**, and **Fig EV4C**). These studies reinforce our conclusion that the FLIM-FRET measurements provide a *bona fide* readout of nanoscale chromatin compaction.

2. We include new data to carefully assess the possibility that DNA damage during the FLIM-FRET measurements might have influenced the data (new **Fig. S2A-C**). We find no evidence that there is any induction of DNA damage caused by the FLIM-FRET methodology, and such a suggested effect can therefore not explain the low nanocompaction of heterochromatin that we observed in ESCs. We also provide new results to show that dense heterochromatin environments do not prevent the two fluorophore-tagged proteins to produce FRET (new **Fig. 2G**), which was another technical query of one of the reviewers.

3. To provide further information on the genomic incorporation of the tagged H2B histones, we now include ChIP studies that assess incorporation at genomic sequence elements, including at the repeat sequences that constitute heterochromatin (new **Fig. 1C,D**). This additional work confirms that fluorescent-tagged H2B histones have a similar level of incorporation at different genomic loci along the genome.

4. We present a more exhaustive description of the term nanocompaction, by describing the FRET phenomenon and its resolution, but also through estimation of the fraction of labelled nucleosomes involved in the FRET. This is now included in the Material and Method, and in a new table (**Table EV3)**.

5. To assess insights into chromosome dynamics, we now provide FRAP measurements of the H2B histone dynamics in ESCs, in the absence of ATP, in the presence of the

SUV420H inhibitor A-196 (new **Appendix. Fig. S1B** and **S4A**, respectively), and after siRNA down-regulation of HP1α, HP1β or HP1α +HP1β (new **Appendix. Fig. S3A-C**). These new data clearly show that the FRET changes that we observed between these different experimental conditions were not due to alterations in the incorporation dynamics of H2B histones.

6. Following the advice of the Reviewers, importantly, we supply new results on other biophysical parameters that could have affected chromatin organisation, such as LLPS, or chromatin mobility (new **Appendix. Fig. S1C, D** and **Fig. EV3E-G**) and show that the changes in nanocompaction observed by FLIM-FRET may in part be related to LLPS and chromatin motion as well. This is now also mentioned in the Discussion.

7. We provide new live-cell FLIM-FRET measurements in differentiated cells (3T3 embryonic fibroblasts) and detect high levels of chromatin nanocompaction in these differentiated cells (new **Fig. 2G**). This finding strongly suggests that low nanocompaction at heterochromatin foci is a feature of undifferentiated cells.

8. To further assess the role(s) of HP1 proteins, we supply new live-cell FLIM-FRET measurements of chromatin nanocompaction upon siRNA-mediated HP1β depletion in ESCs (new **Fig. 4C and Fig. EV4E**). Our combined findings show that HP1α and HP1β play different roles in the nanoscale organisation of heterochromatin.

9. Importantly, we rephrased several passages in the Results and the Discussion to convey a more balanced view of the interpretation of the experimental FRET data, mentioning besides histone proximity, the existence and likely partial contribution of other biophysical processes as well (see also point 6 above).

Although the main conclusions of our study remain unchanged, we feel that our paper has greatly improved thanks to the Reviewers' insightful suggestions.

Point-by-point responses to the reviewers' comments

Reviewer #1:

The authors analyze the compaction of constitutive heterochromatin in ESCs and EpiLCs cells at nanometer resolution using the system FILM-FRET, in which two fluorescent proteins are expressed as histone fusion proteins. The authors have also defined the term 'nanocompaction', which stands for the spatial organization of nanometer-range proximity

between nucleosomes. Interestingly, they showed that there is no correlation between chromatin-dense regions and the regions where FRET is observed. They also showed that the low FRET state in chromatin-dense regions is maintained in an ATP-dependent manner. In addition, the effects of knockdown of the HP1 subtype and inhibition of H4K20me3 on nanocompaction are analyzed. In conclusion, the authors suggested that heterochromatin is not highly condensed as shown by FRET in living cells. This is very provocative and different from the traditional heterochromatin state, which has been discussed mainly in fixed cells.
The results of this study are as surprising as they are interesting. The considerable amount of material and variety of experimental tools and methods used to analyze deacetylation, methylation, the effect of HP1 protein, and nanocompaction in naive and primary stem cells level are highly notable. However, there are several major difficulties with this manuscript which make it unsuitable for publication in EMBOJ.

We thank the reviewer for agreeing that our study is interesting with a notable amount of data, and for providing useful and constructive comments.

Major comments:
1a. The major concern of this manuscript is the fact that all findings depend on FLIM-FRET results. There is a risk that the interpretation of the results obtained may be influenced by the accuracy of FLIM-FRET.

This is a very valid concern. We now verified our FRET measurements by using a technically independent method based on the measurement of the changes in chromatin density as a readout of compaction. For that, we measured the coefficient of variation (CV) of the H2B-GFP signal. This approach has been extensively used to quantify changes in the density of chromatin and indirectly of heterochromatin compaction (Casas-Delucchi *et al*, 2012; Neguembor *et al*, 2021; Grézy *et al*, 2016; Martin *et al*, 2021; Erdel *et al*, 2020). The CV is defined as (σ/m) where σ represents the standard deviation of the H2B-GFP intensity values and μ represents the mean value of H2B-GFP intensity of individual nuclei. In the revised version, we performed a CV analysis upon ATP depletion, and inversely, in TSA-treated ESCs. where we confirmed, respectively, an increase and decrease of chromatin density in the coefficient of variation measurements. This is shown in the **new Appendix FigS1E**. We also validated our FRET measurements in ESCs treated with siRNAs directed against HP1α and HP1 α/β by the CV method (**new FigEV4C**). Finally, we validated our FRET results on ESCs treated with the A-196 inhibitor of SUV40H1/2 enzymes by using the CV method (**new Appendix FigS4B**). Altogether, by using a second independent method based on changes in

the fluorescence intensity levels (CV), we verified that our FRET measurements provide a *bona fide* readout of chromatin compaction levels.

The authors should therefore take into consideration of other factors that could affect FRET efficiency, other than the 'nanocompaction' level. For example, the FRET efficiency may be a combined result of multiple factors such as chromatin rigidity, phase separation level, and chromatin compaction. In heterochromatin regions, the rigidity of chromatin itself and/or the phase separation level may cause the decrease of FRET efficiency even if two labeled nucleosomes are close to each other spatially.

We agree with the reviewer that changes in the FRET efficiencies measured could result from others biophysical parameters (chromatin motion, rigidity, phase separation, protein binding, etc) that influence the physical proximities between labelled nucleosomes. We addressed this concern of this reviewer by providing the following additional experiments:

1) We performed FRAP experiments on the tagged-histones H2B-GFP, upon ATP depletion, upon siRNA directed against HP1α, HP1β and both HP1 α/β, and after A-196 inhibitor treatment. These data are presented in the **new Appendix FigS1B**, **Appendix FigS3**, and **Appendix FigS4A**. respectively. Together, these analyses show that the H2B histone dynamics remain unchanged in all conditions, and cannot explain the nanocompaction changes measured by FRET.
2) As mentioned by the reviewer, phase separation process has been suggested to be one of the drivers of the global and local dynamics of the genome organization. We now explored this hypothesis by treating ESCs with 1,6-hexanediol (1,6-HD), a widely used tool to disrupt phase-separated condensates such as heterochromatin foci. In line with previous observations, we confirmed that nanoscale chromatin compaction was increased upon 5% to 10% of 1,6-HD treatment. This is now shown in the **new Appendix FigS1C and D**. The new data suggest that LLPS, in case it occurs, might contribute to a certain extent to the regulation of nanocompaction as well. In addition, high concentrations of 1,6-HD drastically can affect chromatin motion as well (Itoh *et al*, 2021), so we cannot exclude that the observed increased FRET efficiency was not in part due to other biophysical parameters such as chromatin motion, or phase separation. We revised the manuscript by adding text in the Results section (lines 201-216) and in the Discussion (lines 504-510).

1b. It is also quite concerning to discuss about phenomenon occurring at 10 nm based on the 890 nm intensity of FRET, which has a long wavelength and low resolution. Other experimental methods should be applied to confirm the findings in this paper.

We thank the reviewer for this feedback. We realize that our description of the nanometer scale measurements of the chromatin compaction named “nanocompaction” measured by FRET was not sufficiently clear. FRET is a photophysical phenomenon by which energy transfers from a donor to an acceptor fluorophore when they are close to each-other, between 1-10 nm. We excited the GFP donor fluorophores with a biphoton laser at 890 nm because it is the optimal biphotonic excitation wavelength of GFP. In these conditions, the voxel size is large (x/y/z: 250/250/759nm), so each voxel contains multiple nucleosomes. Considering the volume of ESC nucleus and the average number of nucleosomes in a mammalian cell nucleus, we estimated there to be approximately 2000 nucleosomes per voxel. With about 20% of fluorophore-tagged nucleosomes, this represents around 400 fluorophore-tagged nucleosomes per voxel. Taking this in account, what we measured in a single voxel over the time of an acquisition is an average of FRET efficiencies, corresponding to an average of many direct interactions occurring at the nanometer scale between individual tagged-nucleosomes. We added a new paragraph to the Material and Method (FLIM-FRET measurements section, lines 856-864) to explain these points, and a new **Table EV3** that summarizes the labelling percentage of nucleosomes. We think that the new version now includes all the necessary details.

1c. In addition, it might be expected that there is a mechanism in heterochromatin that prevents the fluorescent proteins used in this study from condensing with each other. To rule out this possibility, the authors should conduct experiments using fluorophores other than GFP and mCherry or change the type of histone to which the fluorescent proteins are bound.

The reviewer raises an interesting point. We now provide additional data to exclude the possibility that heterochromatin could prevent the fluorescent proteins from condensing with each-other:

1) We performed FLIM-FRET measurements in living differentiated 3T3 cells where heterochromatic foci (‘chromocenters’) are clearly visible. We clearly detected high FRET efficiencies at chromocenters, reflecting a close physical proximity between the two fluorescent histone proteins in a dense heterochromatic environment. This is now shown in the **new Fig2G**.

2) Upon ATP depletion (Fig2F) we observed an increase in the number of heterochromatin foci, with acquisition of strong H2B-GFP and mCherry-H2B signals. Within these dense nucleosome-rich foci, we measured high FRET % values, ruling out possible mechanisms preventing the fluorescent proteins to condense with each-other in dense heterochromatin regions.

2a) In this study, the authors found that heterochromatin shows low level of nanocompaction using FLIM-FRET method, even when enriched in H2B-GFP. This observation is very different from the heterochromatin state observed in fixed cells from previous reports. The authors should discuss why they obtain such surprising results, that are different from the results observed in the fixed cells.

As the reviewer mentioned, we found that the level of nanocompaction at heterochromatin in living cells is very different from the heterochromatin compaction state in fixed cells. We strengthened this observation by testing two additional fixation procedures (2%FA/1XHBSS and methanol:ethanol 1:1) and their effect on chromatin nanocompaction. As shown in the **new FigEV3 E-G**, nanocompaction was markedly increased upon 2%FA/1XHBSS and methanol:ethanol 1:1 fixations. Based on these results, we argue that the effect of fixation we observed on chromatin compaction is not specific to fixation procedures derived from aldehyde, but occurs also when one uses organic solvents.
As reported by Itoh *et al*. (2021) in single molecule tracking microscopy, fixation procedures drastically impaired nucleosome dynamics and movements. Taking this into consideration, we propose that, in living ES cells, nucleosomes (and consequently the chromatin fiber) fluctuate constantly between different states, and these dynamic and perpetual changes affect the contact/interaction between nucleosomes themselves or with other surrounding molecules and might contribute to the relative plasticity and low nanocompaction levels of chromatin measured by FRET. In contrast, in fixed cells, the environment of nucleosomes is steady, formed by stable and permanent interactions attributed to adduct formation for cross-linking methods, or precipitation of proteins for the organic solvent methods, which might favor longer average lifetime of a state of specific interactions and FRET to occur. We have now added these considerations and discuss these points in our revised manuscript (Discussion, lines 557-566).

2b) In addition, the authors should show whether the efficiency of H2B-GFP and mCherry-H2B incorporation into chromatin is the same in all chromatin regions.

We thank the reviewers for this relevant suggestion. To further assess the distribution of fluorophore-tagged H2B histones in different genomic contexts, we performed ChIP-qPCR experiments against GFP on parental WT and H2B-GFP ESCs. We show in the **new Fig1C and D** that the GFP-tagged H2B histones present a similar distribution in repetitive sequences that are characteristic of heterochromatin, such as intracisternal A particle (IAP) sequences,

ETn (Early Transposon) elements (ETnERV2) or the pericentromeric major satellite DNA repetitive sequences, and this is comparable to GFP precipitation levels at facultative heterochromatin (*Hoxa11*) and at actively-transcribed genes *(Gapdh, Actb, Pouf5f1-gene body and Pouf5f1-promoter*). In addition, we now show that the fluorophore-tagged H2B histone incorporation does not alter the enrichment of the repressive heterochromatin modification H3K9me3 at the different sequence elements analysed (**new Fig1D).**

3) The correlation between the nanocompacted chromatin state, as defined by the authors, and the function of nanocompaction is unclear. Low FRET signal is observed in chromatin-dense regions while high FRET signal is observed in non-chromatin-dense regions. The authors should identify the chromatin regions where high FRET is observed and clarify the correlation between the chromatin structure and function of that region and nanocompaction.

We thank the reviewer for raising this interesting question. We agree that identifying the chromatin regions where nanocompaction is highest would be interesting, but this was not the not the aim of the manuscript. Nevertheless, we tested the effect of the transcription inhibitor 5,6-dichloro-1-β-d-ribofuranosylbenzimidazole (DRB) on the nanocompaction of chromatin in ESCs. *Reviewer Figure 1* below shows that in the presence of the DRB inhibitor (50ug/ml for 45 min), there is a significant decrease of the chromatin nanocompaction level, suggesting that transcriptional processes may be focally linked to high nanocompaction. However, since we think that the exploration of transcription-related structural effects are not within the main focus of the paper -which is on the nanocompaction state of heterochromatin in stem and differentiated cells- this preliminary insight will not be incorporated in the manuscript.

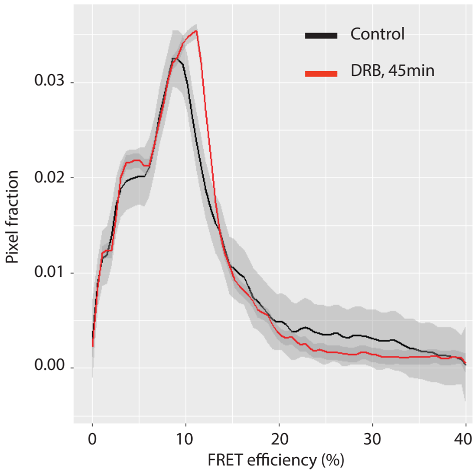

Reviewer Figure 1. A decrease of chromatin nanocompaction after inhibition of transcriptional elongation with DRB. Black and red curves correspond respectively to the FRET-efficiency percentile distributions in control ES cells and ES cells treated with DRB for 45 min.

4) In the region where nanocompaction is happening, FRET occurs, although H2B-GFP fluorescence is not visible. However, since the DNA content is not shown in living cells, the specific area where nanocompaction occurs remains unclear. Therefore, the authors should stain DNA. Since H2B-GFP fluorescence co-localizes with DAPI, Hoechst and DRAQ5 can be used instead to stain living cells.

We thank the reviewer for his comment, and realize that we may not have been sufficiently clear regarding the DNA-content labelling issue. In our study, we show that the colocalization coefficient is high between H2B-GFP and DAPI signal (r= 0.85; Figure EV1 C), which means that H2B-GFP signal reflects DAPI staining levels, and thus the DNA content. In addition, it is technically impossible to stain DNA with DAPI or Hoechst and then perform FLIM measurements between H2B-GFP and mCherry-H2B. DAPI has a very large/broad spectrum of absorption and upon 2-photon excitation of the GFP at 890 nm, DAPI will be excited as well, generating bleed-through into the GFP channel, complicating the FLIM measurements of the GFP donor.

5) Page 5, Line 90-93: The authors cited "Norton et al. 2008" as a previous heterochromatin study, but this paper focuses mainly on perinucleolar compartments. Proper citation is thus required.

We thank the reviewer for spotting this incorrect reference. We corrected it.

6a) The authors evaluated the effect of ATP depletion on nanocompaction and observed a loss of activity of ATP-dependent chromatin remodeling factors. First, such an interpretation is difficult, although high FRET signals were observed in chromatin-dense regions, because ATP depletion deeply impacts the cells, such as the shutdown of all phosphatases and enzymes involved in ubiquitination. Therefore, the authors should further investigate the cause of the observed nanocompaction change by performing knockdown analyses of ATP-dependent chromatin remodeling factors.

We thank the reviewer for this suggestion. We agree that ATP depletion might affect multiple cellular pathways. However, in our study we used ATP-depletion to demonstrate that chromatin nanocompaction in ESCs is dynamically regulated and that these changes can be

monitored in living cells using our FLIM-FRET technique. We think that investigating the specific roles of ATP-dependent chromatin remodelling factors might be out of scope for our study, and would represent a new research project on its own.

6b) Secondly, the authors cited "Iurlaro et al., 2021, Schick et al., 2021" as references when they mentioned that "ATP depletion leads to loss of activity of ATP-dependent chromatin remodeling complexes" (Page 8, Line 170-171). These studies are not related to ATP-depletion but are focused on the intracellular function of the ATPase subunits of a remodeler, by using reagents that specifically inhibit the ATPase activity, or the method targeted degradation, respectively. Therefore, the authors should cite more appropriate references.

We thank the reviewer for spotting these incorrect references. We removed these two citations.

7) The authors concluded that HP1α and HP1β have different effects on nanocompaction level. However, the authors only observed HP1α-depleted cells and HP1α/HP1β-depleted cells, but not HP1β-depleted cells. Since it has been reported that knockdown of HP1 subtype changes the expression of other subtypes, it is necessary to conduct knockdown experiments of HP1β alone to analyze the effects of HP1α and HP1β on nanocompaction.

We thank the reviewer for raising this point. We now include siRNA knockdown against HP1β alone, presented in the new **Fig. 4C and Fig. EV4E**. Interestingly, this analysis shows that the HP1β isoform favours heterochromatin compaction, whereas HP1α restraints it. Combined our data on the HP1 isoforms support the notion that HP1α and HP1β have different roles in the nanoscale organisation of heterochromatin.

Reviewer #2:

This paper reports on reduced FRET in living cell heterochromatin that has incorporated histone H2B-EGFP and H2B-mCherry. The authors show that fixed cells have increased FRET and argue that fixation is responsible for the observed dense organization of heterochromatin. The data most certainly is intriguing and may be consistent with the freedom of movement of chromatin within heterochromatin domains. However, there is significant evidence that nucleosomes within dense chromatin undergo less diffusion than nucleosomes in euchromatic regions of the genome. While not direct evidence, this suggests increased crosslinking of chromatin fibers within heterochromatin domains. Moreover, the observed fluorescence intensity of both the mCherry and the EGFP tagged histone H2B demonstrate increased chromatin densities wthin the heterochromatic regions of the genome. The authors misinterpret the data on accessibility, which clearly shows reduced access of freely diffusing nuclear probes to the interior of heterochromatin is restricted at higher molecular weights while accessibility is increased in the presence of histone deacetylase inhibitors (PMID: 16317046).

A) Moreover, they ignore FLIM experiments that show changes in fluorescence lifetime that correlates with condensation state (PMID: 26765322).

Based on the reviewer's comment, we realize that our description of nucleosome density versus the proximity between nucleosomes measured by FLIM-FRET may not have been sufficiently clear. In the paper cited by this reviewer (PMID: 26765322), chromatin compaction was assessed by measuring the fluorescence lifetime of DNA-intercalating compounds. First, their approach is different from ours, in which we measure chromatin nanocompaction by using proteins (H2B histones) that are stably bound to chromatin and where both the GFP- and the mCherry fluorophore tags are protruding (see structural modelling in FigS1, Llères et al., JCB 2009). Consequently, both fluorophores are less affected by changes in local parameters of the DNA-helix, such as viscosity, polarity, quenching. Second, all along our study, we measured and used as a reference the fluorescence lifetime of the H2B-GFP in the same conditions as for the FRET measurements (drug treatments, siRNAs, ATP depletion, fixation, etc). By doing that, we took in consideration a possible variation of the fluorescence lifetime of the donor caused by changes in the surrounding environments and/or experimental conditions.

B) Most notable is the application of the same method of H2B FRET in NIH-3T3 cells (PMID: 29535103 Figure 2C). In this time-lapse experiment, we can see a decrease in heterochromatin FLIM over time. One explanation for this would be the accumulation of DNA damage during imaging, driving decondensation.

We thank the reviewer for this helpful comment. We provide new results on DNA damage upon FLIM-FRET measurements (new **Fig. S2A-C**). Particularly, we performed time-lapse FLIM-FRET acquisitions in live ESCs and 3T3 cells transfected with EGFP-53BP1, and found neither appearance nor accumulation of DSB foci during FRET imaging, indicating a lack of DNA damages. DNA damage accumulation does therefore not explain the low nanocompaction that we measured at heterochromatin foci in living cells.

C) Finally, their own previous and cited C. elegans study (PMID: 28199849) obtains the opposite result-HP1 proteins promote condensation. These studies contradict the conclusions of this paper and, hence, require that we scrutinize the interpretation of the results of the current study.

We respectfully disagree with the reviewer's comment. Indeed, it is difficult to compare our present work performed in mouse pluripotent ESCs, with the mentioned earlier study (PMID: 28199849) performed on germ cells in a different organism (*C. elegans*). In our previous *C. elegans*' paper, the FRET study and the HP1 results were obtained on pachytene cells, which are meiotic differentiated cells, whereas the present study of HP1 roles is performed in pluripotent embryonic stem cells.

Major concerns:

1) Given the concerns about DNA damage, there is a requirement to assess whether or not these cells are experiencing DNA damage. In particular, PARP activation might explain the decreased chromatin density in heterochromatin. Treatment with PARP inhibitors prior to imaging and staining of the cells for PAR accumulation after imaging (fixation and staining post live cell imaging) would address these concerns. Note that PAR has a short lifetime and may be difficult to detect if fixation is delayed significantly following imaging.

We thank the reviewer for this valid point. As mentioned above, we provide new data on a lack of DNA damage upon FLIM-FRET measurements (new **Fig. S2A-C**). For that, we performed time-lapse FLIM-FRET acquisitions in live ESCs and 3T3 cells transiently expressing an EGFP-53BP1 construct. 53BP1 is a protein involved in double-strand break (DSB) repair and is recruited rapidly to the DNA damaged sites. We found neither appearance nor accumulation of DSB foci during FRET imaging. This finding shows that cells do not experience DNA damage

during the FRET imaging, and that therefore this can not explain the observed low nanocompaction within the heterochromatin foci in living cells.

2) What the authors directly demonstrate is that FRET increases upon fixation. The relative FRET observed in a previous similar study did not reveal decreased density in heterochromatin. They claim that the nuclear volume is maintained under these conditions. In our hands, there is condensation but it is accompanied by a decrease in nuclear volume. The authors could validate this effect by placing cells in hypertonic media, which will generate the same phenomenon.

We thank the reviewer for this suggestion. ATP depletion is routinely used in the field to induce compaction of chromatin (see Platani *et al*., Nat Cell Biol, 2002; Nozaki *et al*., Molecular Cell, 2017; Spagnol *et al*., PLOS ONE, 2016). In the present study, we used a brief ATP-depletion that did not affect drastically the nuclear shape, to demonstrate that the nanocompaction of chromatin in ESCs is dynamically regulated and can be monitored in living cells using our FLIM-FRET approach. *Reviewer Figure 2* (see below) shows that in the presence of sucrose (40mM), to induce hypertonic stress within a few minutes, we massively affected the ESC cells' morphology, and their nuclear shape. For these reasons, we think that mimicking the ATP depletion effect on chromatin by placing cells in hypertonic media is unnecessary and will not bring additional insights to the study.

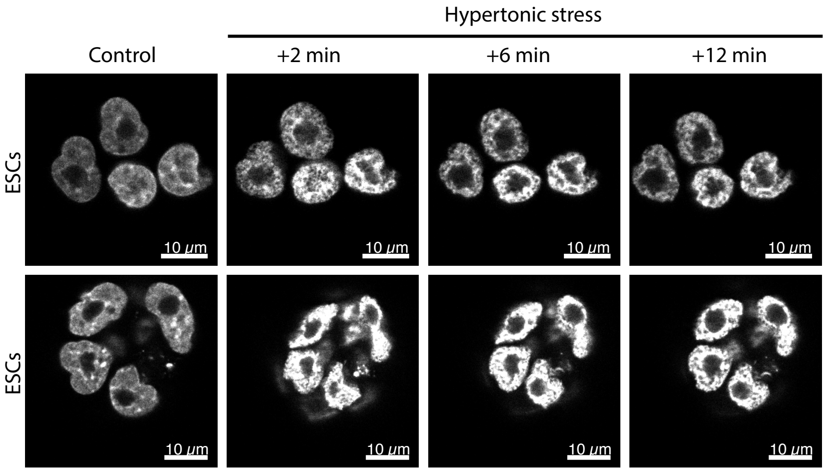

Reviewer Figure 2. ESCs upon hypertonic stress. Cells were placed in a hypertonic media containing 40mM sucrose. After a few minutes of incubation, the ES cells' nuclear morphology was drastically affected. The upper and lower panels represent two different ES cell colonies before and after hypertonic stress.

3) An alternative explanation is that the heterochromatin undergoes more nucleosome-nucleosome interactions (reduced nucleosome spacing) in the absence of fixation than the

surrounding chromatin but the incorporation of the histones is reduced such that the FRET efficiency is lower. Fixation will still generate increased FRET signal but neither can be directly compared to regions outside of the heterochromatin because of differences in the density of FP-H2B incorporation.

Possibly, we mis-interpret this comment of the reviewer, regarding how the incorporation of histones could be affected by the fixation procedure. However, we now provide additional results by testing two additional fixation procedures (2%FA/1XHBSS and methanol:ethanol 1:1) and measuring their effect on chromatin nanocompaction by FRET. As shown in the **new FigEV3E**, nanocompaction was largely increased upon 2%FA/1XHBSS and methanol:ethanol 1:1 fixations as well. Importantly, we quantified both H2B-GFP and mCherry-H2B fluorescence intensities after cell fixation and this shows that the chromatin incorporation of these fluorescent H2B histones was not perturbed by the fixation procedures (**new FigEV3E-G).**

4) A more direct test of the relative incorporation of the fluorescent proteins into chromatin is required. I would suggest that the EGFP be used for ChIP experiments to demonstrate that representative active, facultative heterochromatin (repressed tissue specific genes repressed by polycomb), and satellite sequences associated with constitutive heterochromatin. If the histone is evenly distributed, these experiments should reveal no significant enrichment or depletion of any of these classes of sequences. Alternatively, ChIP-seq experiments could be done. Again, the expectation is a very homogeneous distribution.

We thank the reviewer for raising this important point. We provide new ChIP directed against GFP on parental WT and H2B-GFP ESCs, to assess the distribution of fluorescent-tagged H2B histones in different genomic contexts. We present these data in the **new Fig1C and D,** and show that fluorophore-tagged H2B histones present a similar distribution at repetitive sequences characteristic of heterochromatin such as intracisternal A particle (IAP) sequences, ETn (Early Transposon) elements (ETnERV2) or pericentromeric major satelite DNA repetitive sequences, similarly as at facultative heterochromatin (*Hoxa11*) and actively transcribed genes *(Gapdh, Actb, Pouf5f1-gene boy or Pouf5f1-promoter*). In addition, we confirmed that fluorescent-tagged H2B histone incorporation did not alter the enrichment for the H3K9me3 repressive mark at heterochromatin (**new Fig1D).**

5) Ultimately, the claim of fixation-dependent changes in chromatin organization is difficult to evaluate in the absence of demonstrating the same phenomena in a more commonly studied cell line where the chromatin state is better characterized. Most suitable for this study would

be the use of NIH 3T3 cells, where there is existing live cell FLIM data using this H2B system (PMID: 29535103).

We thank the reviewer for this suggestion. We provide new FLIM-FRET measurements of chromatin nanocompaction in better characterized differentiated 3T3 fibroblast cells (new **Fig. 2G**), and show that low nanocompaction at heterochromatin foci observed in pluripotent cells highly contrast with the one measured in differentiated 3T3 fibroblast cells. In addition, we now test two additional fixation procedures (2%FA/1XHBSS and methanol:ethanol 1:1) on chromatin nanocompaction by FRET. We show in the **new FigEV3E**, that the nanocompaction was largely increased upon 2%FA/1XHBSS and methanol:ethanol 1:1 fixations as well.

6) The results on K20 methylation are interesting in their own right. However, the distinction between di and trimethylation of K20 is important. Dimethylation is very abundant and found throughout the genome whereas the trimethylation is much more restricted to heterochromatin.

We thank the reviewer for this excellent suggestion. We provide new immunostaining experiments (**new Fig4G**) and western blot analysis (**new FigEV4H**) to study the levels of H4K20me2 as well. We show by western blotting that A-196 treatment leads to a marked H4K20me3 reduction, and has a lesser effect on H4K20me2 as well. We now modified several passages in the Results section (e.g., line 407, line 415), and in the interpretation of the FRET data obtained upon A-196 treatment through the contribution of H4K20me2.

7) These experiments and the related HP1 experiments would be nicely complemented by the addition of suv39h1/h2 knockout to eliminate the K9 trimethylation, HP1, and downstream K20 trimethylation specifically in constitutive heterochromatin.

This is an interesting comment. *Reviewer Figure 3* below shows the levels of H4K20me3, and of HP1α, in *Mki67* KO ES cells, in which a significant decompaction of the chromatin was observed by FRET. H4K20me3 levels were significantly reduced in these *Mki67*$^{-/-}$ cells, as shown both western blotting analysis and immunostaining experiments. Interestingly, we find that also HP1α levels were increased in the ES cells KO for *Mki67*. Although these preliminary insights have not been incorporated in the manuscript, they support our hypothesis that HP1α reduces, and H4K20me3 increases, the nanocompaction levels of heterochromatin.

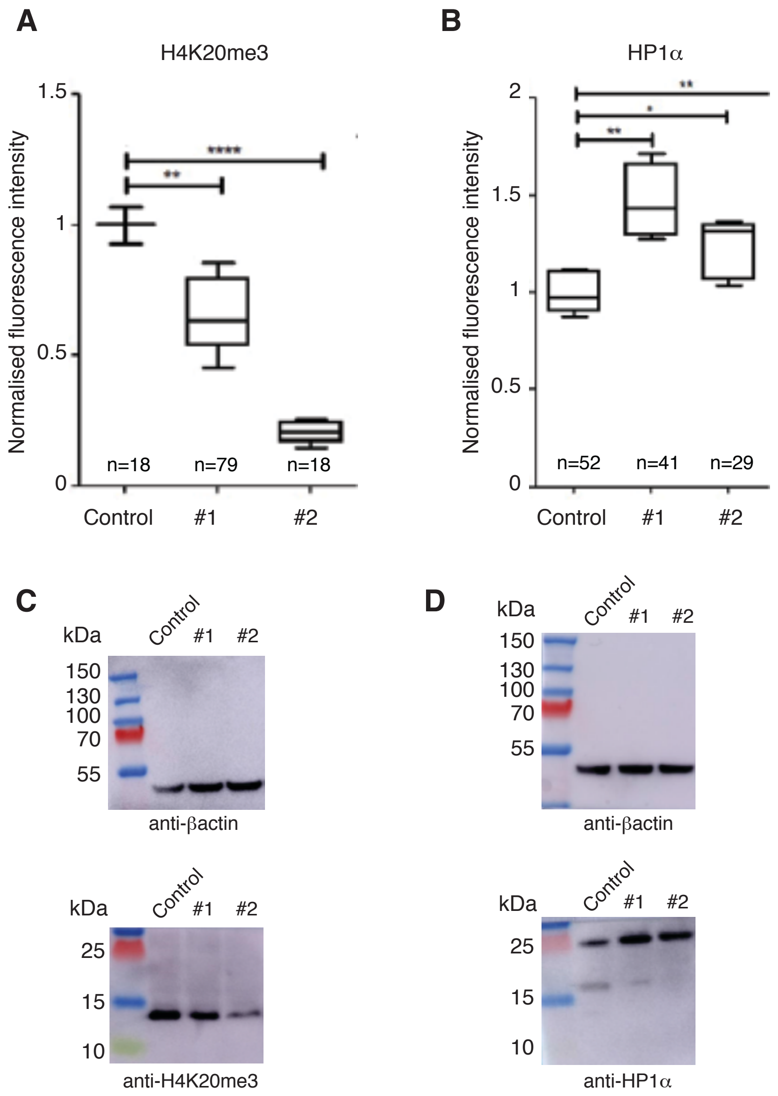

Reviewer Figure 3. **A**) Quantifications of immunostaining for H4K20me3 in control ESCs and *Mki67*$^{-/-}$ ESCs clones #1 and #2. **B**) Quantifications of immunostaining for HP1α in control ESCs and *Mki67*$^{-/-}$ ESCs clones #1 and #2. **C**) Total cell extracts from control ESCs and *Mki67*$^{-/-}$ ESCs clones #1 and #2, analysed by western blotting with antisera against H4K20me3 (bottom panel) and βactin (top panel, loading control). **D**) Total cell extracts from control ESCs and *Mki67*$^{-/-}$ ESCs clones #1 and #2, analysed by western blotting with antisera against HP1α (bottom panel) and βactin (top panel, loading control).

Reviewer #3:

Constitutive heterochromatin, which is mainly formed at the pericentromeric regions, is thought to be condensed and transcriptionally silenced. The formation relies on the recruitment of HP1, which can crosslink nucleosomes containing histone H3K9me3 and promote chromatin compaction. However, the physical state of heterochromatin in living cells is not fully understood. Using the FLIM-FRET between nucleosomes, where H2B-EGFP and mCherry-H2B were incorporated, Dupont et al. measured chromatin compaction in living mESCs. The authors found that the FRET efficiency at the chromocenter heterochromatin in living cells is lower than in other regions. In contrast, in formaldehyde-fixed cells, the FRET efficiency of heterochromatin increased. Based on this data, they claimed that the heterochromatin at the chromocenter is less condensed in living cells. The authors showed that HP1α knockdown increased FRET efficiency in living ESCs, but this effect was overridden by loss of HP1β. Furthermore, depletion of H4K20me3 decreased the FRET efficiency. Finally, knockout of Ki-67 strongly reduced the FRET efficiency in interphase and mitotic heterochromatin in ESCs. The obtained findings are at odds with the prevailing models and intriguing and thought-provoking in the chromatin-related fields. However, there are several points to be addressed for publication in The EMBO journal. My specific comments and suggestions are the following:

Major comments:

1a) Line 204 (Fig. 2A). "we observed no correlation between fluorescence intensity levels (i.e., H2B-GFP density) and FRET efficiency (Pearson coefficient r =-0.07, n=106 cells)". Although this is a really exciting point, it is possible that FRET efficiency is not necessarily related to chromatin compaction. H2B-GFP intensity in confocal microscopy imaging of living cells should be related to the nucleosome density (chromatin compaction) if there is no bias for H2B-GFP-incorporation in the genome. There are a number of such data in the cited references.

I wonder whether FRET efficiency could be affected by not only distances between H2B-GFP and mCherry-H2B, but also other factors in the chromatin environment, such as relative angles of the fluorescent probes, associated protein (e.g., HP1α), and so forth. If the chromatin-associated HP1α somehow disturbs the FRET efficiency in living cells, the authors' whole story may collapse. Note that the binding mode of HP1α also can be changed in the formaldehyde-fixed cells. The authors should address this issue.

Based on the reviewer's comments, we realize that our definition of the nanoscale compaction termed "nanocompaction" may not have been sufficiently clear and certainly needed to be better defined.

1) As reported by reviewer #3, we do not claim that H2B-GFP intensity in confocal microscopy is not related to nucleosome density. On the contrary, we show that the colocalization coefficient is high between H2B-GFP and DAPI signal (r= 0.85; Figure EV1 C), which means that H2B-GFP signal reflects DAPI staining levels, and thus nucleosome density. In addition, we now include ChIP studies that assess incorporation at genomic sequence elements, including at the repeat sequences that constitute heterochromatin (new **Fig. 1C,D**). This additional work confirms that fluorescent-tagged H2B histones have a similar level of incorporation at different genomic loci along the genome.
2) In our study, we correlate the H2B-GFP intensity (nucleosome density) which is in the pixel range (x/y:250/250nm) with FRET efficiency, which is occurring at a different scale of organization (nanometer range). So, what we measured in a single voxel over the time of an acquisition is an average of FRET efficiencies, corresponding to an average of many direct interactions occurring at the nanometer scale between individual tagged-nucleosomes. We added a new paragraph to the Material and Method (FLIM-FRET measurements section, lines 856-864) to explain these points.
3) We agree with the reviewer that changes in the FRET efficiencies measured could result from others factors and biophysical parameters (chromatin motion, rigidity, phase separation, protein binding, etc) that influence the physical proximities between labelled nucleosomes. We addressed this concern of this reviewer by providing the following additional experiments:
    a. We performed FRAP experiments on the tagged-histones H2B-GFP upon siRNA directed against HP1α, HP1β and both HP1 α/β. These data are presented in the **new Appendix FigS1B**, **Appendix FigS3**, and **Appendix FigS4A**. respectively. Together, these analyses show that the H2B histone dynamics remain unchanged in all conditions, and cannot explain the nanocompaction changes measured by FRET.

    b. Phase separation process has been suggested to be one of the drivers of the global and local dynamics of the genome organization and recent findings suggest that HP1α protein can undergoes liquid-liquid phase separation (LLPS) *in vitro* to condense DNA (Larson *et al*, 2017). We now explored this hypothesis by treating ESCs with 1,6-hexanediol (1,6-HD), a widely used tool to disrupt phase-separated condensates such as heterochromatin foci. In line with previous observations, we confirmed that nanoscale chromatin compaction was increased upon 5% to 10% of 1,6-HD treatment. This is now shown in the **new**

Appendix FigS1C and D. The new data suggest that LLPS, in case it occurs, might contribute to a certain extent to the regulation of nanocompaction as well.

c. As reported by Itoh et al, 2021, 1,6-HD drastically can affect chromatin motion and fixation procedures drastically impaired nucleosome dynamics and movements as well. We strengthened this observation by testing two additional fixation procedures (2%FA/1XHBSS and methanol:ethanol 1:1) and their effect on chromatin nanocompaction. As shown in the **new FigEV3 E-G**, nanocompaction was markedly increased upon 2%FA/1XHBSS and methanol:ethanol 1:1 fixations suggesting that some of the observed high nanocompaction may therefore come from the suppression of the chromatin motion effects.

Taken together b) et c) points, we cannot exclude that the observed increased FRET efficiency was not in part due to other biophysical parameters such as chromatin motion, or phase separation. We have now added these considerations (Results, line 201-216) and discuss these points in our revised manuscript (Discussion, lines 504-510).

4) As mentioned by the reviewer, recently HP1α was characterized as a chromatin crosslinker (Strom *et al*, 2021), but HP1α proteins bind chromatin transiently with an exchange time on the order of seconds (Phair *et al*, 2004; Cheutin *et al*, 2003). At the molecular level, it remains unclear how precisely HP1 regulates the nanoscale heterochromatin structure in living cells. Our work in primary cells suggests that HP1α may contribute to the plasticity of nucleosomes. Mechanistically, we can not rule out that perturbations of the chromatin stiffness due to the partial loss of HP1α binding (Strom *et al*, 2021) may alter the distance between nucleosomes or the frequency of nucleosome proximities monitored by FRET. We largely revised the manuscript by adding text in the Discussion (lines 574-585). Whether HP1 binding affects the orientation of the fluorescent probes: this issue was taken into account in the original design of our assay and of the two constructs that express the two respective fluorophore-tagged histones. Particularly, we included a 16 aa protein linker between the mCherry moiety and histone H2B and a 6aa linker between histone H2B and EGFP, to ensure that the fluorophore is positioned away from the nucleosome (Table S1, Lleres et al., JCB 2009). This external positioning of the Donor and Acceptor fluorophores likely reduces the steric hindrance caused by the binding of proteins (including HP1) or other chromatin-related proteins to DNA, and consequently, minimizes the structural perturbation to the FRET. Nevertheless, there is still a

possibility that HP1 binding could affect the “dipole-dipole” vector orientation of the GFP-mCherry FRET pair, without affecting the distance/proximity between nucleosomes. This may have influenced the interpretation of the part of the studies where we functionally explored the effects of HP1 isoforms. For these reasons, we decided to slightly tune-down our main conclusions in the main text, and by writing “Evidence for low levels of nanocompaction at heterochromatin’ in the title.

2) "Constitutive heterochromatin shows low nanocompaction in living ESCs." I wonder whether the low FRET efficiency in the constitutive heterochromatin is unique in living ESCs or not. It is essential to know whether it is true for somatic mouse cells, which have chromocenters. For instance, the immortalized and differentiated cell line such as NIH3T3?

We thank the reviewer for this suggestion. We provide new FLIM-FRET measurements of chromatin nanocompaction in better characterized differentiated 3T3 mouse fibroblasts (new **Fig. 2G**), and this now strongly suggests that low nanocompaction at heterochromatin is a feature of undifferentiated cells.

3) Line 307-309. "upon siRNA-mediated HP1α/β co-depletion in BJH2B-2FPs ESCs..." The authors may want to know whether HP1β-single depletion reduces FRET efficiency to the same extent as HP1α/β co-depletion.

We thank the reviewer for raising this point. We now provide siRNA knockdown analysis against HP1β alone in the new **Fig. 4C and Fig. EV4E**. This analysis shows that the HP1β isoform favours heterochromatin compaction whereas HP1α restraints it. These new data support the notion that HP1α and HP1β isoforms have different roles in the nanoscale organisation of the heterochromatin.

4) Line 456-458. "Our data on living cells suggest that ..." Could authors **explain** how the more plastic and dynamic nature of heterochromatin contributes to some cellular functions such as transcriptional silencing?

We agree with the reviewer that lines 456-458 (from the original version of the manuscript) required additional explanations. We now provide further explanations on the plastic and dynamic nature of heterochromatin in ESCs by discussing the different biophysical properties of heterochromatin at the nanoscale versus mesoscale organisation (Discussion, lines 557-

566). We are mentioning the importance of the expression of satellite RNAs from heterochromatin in the cellular functions of ESCs (Introduction, lines 72-75).

1) Line 185-186. “TSA treatment strongly increased global histone tail acetylation …” The difference is not so prominent. The senior author previously performed a similar experiment with TSA-treated HeLa cells (Llères et l., JCB 2009, Figure 5B), and found a clear difference between untreated and TSA-treated cells. This point should be clarified.

We thank the reviewer for raising this point. In our previous study (Llères et al., *JCB* 2009, Figure 5B), HeLa cells were treated with 200 ng/ml TSA for 24 h, whereas in the present study, TSA was added to naïve ES cells to a final concentration of 200 ng/ml, for 9h. Histone acetylation has been shown to be important for the pluripotent cell state and treatment with some HDAC inhibitors has been shown to improve reprogramming into iPSCs and to maintain the pluripotent stem cell state. Indeed, it is thought that pluripotent cells have a higher overall level of histone acetylation than fully differentiated cells (Meshorer E, et al. *Dev Cell.* 2006; Efroni S, et al. *Cell Stem Cell.* 2008; Krejci J, et al. *J Cell Physiol.* 2009). Taken together, these effects could explain the less pronounced effect of TSA treatment in the naïve ESCs used in the study, compared to the earlier study on HeLa cells.

Minor comments:

1) Why did the authors use H2B-GFP and mCherry-H2B for FILM-FRET, but not H2B-mCherry?

We apologize for the lack of clarity on this specific point. In the original design of our FRET assay, the EGFP tag was on the C-terminal side of H2B whereas the mCherry tag was on the N-terminal side, which in an earlier structural model showed that FRET can only occur away from the nucleosome (see Figure S1A, Lleres et al., *JCB* 2009). Although the probability of having two fluorophores tagged H2B histones within the same nucleosome is extremely low (0.38%, **new Table EV3**), by using these constructs we avoided the occurrence of any intra-nucleosome FRET. We provide now more details on this point in the revised version of the manuscript (lines 153-155).

2) Line 384- 386. “In addition, we noticed that two different populations of FRET efficiencies ...”

I am not sure if two different populations of FRET efficiencies were observed only in EpiLCs.

Even in mESCs (CTRL) of Figure 5D, the FRET efficiency distribution seems to have two different populations. Why?

We thank the reviewer for raising this point. These two different populations of FRET could represent two different states of nanocompaction within the nucleosome-rich foci of EpiLCs, or two different classes of heterochromatin within nuclei. We also can not exclude that these two different populations in EpiLCs could represent two different cell populations depending on their acquired differentiation state, although morphologically the cells were completely homogeneous as they were growing on fibronectin coated plates. We further comment on this in the Results (lines 477-478).

3) There are typos in the manuscript as described below.
Figure 1. "Figure 1f" and "Figure 1g" -> "Figure 1F" and "Figure 1G". .
Line 320. "Expanded View Fig 4D" -> "Expanded View Fig 4E".
Line 350. "Fig. 5d" -> "Fig. 5D".
We apologize for these typos and corrected them in the new version of the manuscript.

4) Line 481. "Because of" -> "Because"?
Line 511. "the interplay of these components in and..." -> "the interplay of these components and..."?
We thank the reviewer for pointing this out to us. We have corrected this in the new version of the manuscript.

Dr. David Llères
Institute of Molecular Genetics of Montpellier (IGMM), CNRS, University of Montpellier
IGMM
1919 Route de Mende
Montpellier 34293
France

31st Jan 2023

Re: EMBOJ-2021-110286R-Q
Heterochromatin has low levels of nanocompaction in living embryonic stem cells

Dear Dr. Llères,

Thank you for submitting a revised version of your manuscript EMBOJ-2022-110286, which we had previously rejected after review. As we had discussed, I contacted the initial three referees and we have now received their comments on the revised version and your response to their concerns (please see below). I discussed these comments with members of the editorial team, as well as again contacting the referees for cross-commenting on the reports. Taking everything into account, we have decided to proceed further with manuscript, and to invite you to resolve the remaining issues in a final revision.

As you will see, the reviewers overall appreciate that you have added experiments and revised the manuscript in response to their comments. However, they are not yet satisfied with the discussion of the method and its potential limitations. Thus, we would ask you to carefully consider the referee comments and to revise the manuscript as needed, in particular by expanding the discussion. Here, it will be important to clarify the findings in the context of condensation with and without nucleosome fluctuations, as referee #3 discusses. We will not strictly require further experimental validation (referee #1) to proceed with the manuscript, also noting that such methods may have their own specific technical limitations, as was brought up during referee cross-commenting. However, it is crucial that you revise the manuscript to ensure that all conclusions are fully supported by data or appropriately discussed. Please also provide a detailed response to each reviewer comment when submitting the revised manuscript. In addition, please remember that the revised manuscript should fulfill all EMBO Journal formatting requirements when it is next submitted (please see below and:
https://www.embopress.org/page/journal/14602075/authorguide#submissionofrevisions)

Please note that it is our policy to normally allow only a single round of major revision. Thus it is important to clarify any questions and concerns as early as and I invite you to contact us to discuss this final revision and to clarify any questions or potential uncertainties. In this regard, I would also like to let you know that I will be leaving EMBO Journal this February and I will hand over your manuscript to one of my current colleagues. This will not affect any decisions on your study and anything we have discussed and agreed upon will be noted and accessible to the new handling editor.

Thank you for the opportunity to consider your work for publication. Please contact me if you would like to discuss any points further or have any questions.

Kind regards,
Stefanie

Stefanie Boehm
Editor
The EMBO Journal

***IMPORTANT NOTE: we now perform an initial quality control of all revised manuscripts before re-review. Your manuscript will FAIL this control and the handling will be DELAYED if the following APPLIES:

1) A data availability section is missing. (The section must be named "Data Availability" and must have the required format, detailed below). Please provide either a referee token or public access to all data.
2) Your manuscript contains error bars based on n=2. Please use scatter blots showing the individual datapoints in these cases. The use of statistical tests needs to be justified.

When submitting your revised manuscript, please carefully review the instructions that follow below. Failure to include requested items will delay the evaluation of your revision.***

-

When submitting your revised manuscript, please carefully review the instructions below and include the following items:

1) A .docx formatted version of the manuscript text (including legends for main figures, EV figures and tables). Please make sure that the changes are highlighted to be clearly visible.

2) Individual production quality figure files as .eps, .tif, .jpg (one file per figure). *Please remove figures from the main manuscript*

3) A .docx formatted letter INCLUDING the reviewers' reports and your detailed point-by-point response to their comments. As part of the EMBO Press transparent editorial process, the point-by-point response is part of the Review Process File (RPF), which will be published alongside your paper.

4) A complete author checklist, which you can download from our author guidelines (https://wol-prod-cdn.literatumonline.com/pb-assets/embo-site/Author%20Checklist%20-%20EMBO%20J-1561436015657.xlsx). Please insert information in the checklist that is also reflected in the manuscript. The completed author checklist will also be part of the RPF.

5) All corresponding authors are required to supply an ORCID ID for their name upon submission of a revised manuscript.

6) At EMBO Press we ask authors to provide source data for the main manuscript figures. Our source data coordinator will contact you to discuss which figure panels we would need source data for and will also provide you with helpful tips on how to upload and organize the files.

7) Before submitting your revision, primary datasets produced in this study need to be deposited in an appropriate public database. The required format for this section is:

[data type]: [name of the resource] [accession number/identifier/doi] ([URL or identifiers.org/DATABASE:ACCESSION])
*** Note - All links should resolve to a page where the data can be accessed. ***

Please also see https://www.embopress.org/page/journal/14602075/authorguide#datadeposition Please note that the Data Availability Section is restricted to new primary data that are part of this study.

8) When assembling figures, please refer to our figure preparation guideline in order to ensure proper formatting and readability in print as well as on screen:
https://bit.ly/EMBOPressFigurePreparationGuideline
See also guidelines for figure legends: https://www.embopress.org/page/journal/14602075/authorguide#figureformat

Please remember: Digital image enhancement is acceptable practice, as long as it accurately represents the original data and conforms to community standards. If a figure has been subjected to significant electronic manipulation, this must be noted in the figure legend or in the 'Materials and Methods' section. The editors reserve the right to request original versions of figures and the original images that were used to assemble the figure.

9) We replaced Supplementary Information with Expanded View (EV) Figures and Tables that are collapsible/expandable online (see examples in http://msb.embopress.org/content/11/6/812). A maximum of 5 EV Figures can be typeset. EV Figures should be cited as 'Figure EV1, Figure EV2" etc.. in the text and their respective legends should be included in the main text after the legends of regular figures.

- For the figures that you do NOT wish to display as Expanded View figures, they should be bundled together with their legends in a single PDF file called *Appendix*, which should start with a short Table of Content. Appendix figures should be referred to in the main text as: "Appendix Figure S1, Appendix Figure S2" etc. See detailed instructions regarding expanded view here: < https://www.embopress.org/page/journal/14602075/authorguide#expandedview>.

- Additional Tables/Datasets should be labeled and referred to as Table EV1, Dataset EV1, etc. Legends have to be provided in a separate tab in case of .xls files. Alternatively, the legend can be supplied as a separate text file (README) and zipped

together with the Table/Dataset file.
** We suggest uploading table S1, S2, S3 as datasets. **

10) Our journal encourages inclusion of *data citations in the reference list* to directly cite datasets that were re-used and obtained from public databases. Data citations in the article text are distinct from normal bibliographical citations and should directly link to the database records from which the data can be accessed. In the main text, data citations are formatted as follows: "Data ref: Smith et al, 2001" or "Data ref: NCBI Sequence Read Archive PRJNA342805, 2017". In the Reference list, data citations must be labeled with "[DATASET]". A data reference must provide the database name, accession number/identifiers and a resolvable link to the landing page from which the data can be accessed at the end of the reference. Further instructions are available at < https://www.embopress.org/page/journal/14602075/authorguide#referencesformat >.

Further information is available in our Guide For Authors:
https://www.embopress.org/page/journal/14602075/authorguide

****
In the interest of ensuring the conceptual advance provided by the work, we recommend submitting a revision within 3 months (1st May 2023). Please discuss the revision progress ahead of this time with the editor if you require more time to complete the revisions. Use the link below to submit your revision:

Link Not Available

****

Referee #1:

In order to analyze the physical state of heterochromatin structure in living cells, the authors established a method for measuring nucleosome aggregation in which GFP and mCherry are expressed as histone fusion proteins and allow FRET measurements. Importantly, they define the compaction caused by nucleosome aggregation as "nanocompaction" and performed its measurement in living mESCs and EpiLCs. The authors found that heterochromatin exhibits low levels of chromatin nanocompaction measured by FRET, a claim that runs counter to the conventional notion of heterochromatin, which states that nucleosomes are aggregated in heterochromatin. That is a very interesting and intriguing statement that needs to be examined carefully.
In the previous version of the article, the authors discussed the fact that they found no correlation between chromatin-dense regions and regions where high FRET is observed, but rather a weak FRET state (Fig. 2C, 2D). However, it was questionable whether their FRET system was able to properly detect chromatin aggregation. Without showing that the same results can be obtained via other technically independent systems or without the addition of a positive control, I wondered if the interpretation of the results obtained could be influenced by the accuracy of their unique experimental system, thus casting doubt on the authors' statements.

In their revised version, the authors evaluated their FRET detection system in more detail, notably by performing a new experiment with 3T3 cells. They observed a much higher FRET efficiency in the regions where chromatin seems to be aggregated. However, there is an important concern about this control experiment. In ESCs, the expression of GFP-H2B and H2B-mCherry relative to the total amount of H2B was analyzed by electrophoresis, the result of which suggests that approximately 20% of all nucleosomes contain a single fluorescent H2B. Because the expression level of GFP-H2B and H2B-mCherry is certainly influencing a lot on the intensity of the FRET signal, its expression level should be similar to that of the experiment using ESCs to correlate the results for both cell lines. Unfortunately, the expression levels of GFP-H2B and H2B-mCherry in 3T3 cells, which were used as control cells, are not shown, which would be essential to support those new results.

The authors spent a lot of effort to address my concerns and have undertaken several additional experiments. The rigor of their work is commendable. On the other hand, despite the revised version of the manuscript, it still appeared difficult to discuss and conclude about nanocompaction based on their FRET detection system alone. They also tested another method, the CV measurement. However, while this method was found to be good for studying the degree of chromatin aggregation in the overall nucleus, it is unclear whether this method is appropriate for analyzing nanocompaction. The authors should demonstrate the phenomenon of nanocompaction in heterochromatin using various experimental systems. For example, they could try a proximity ligation assay, to confirm the observations on nanocompaction.

To sum up, this manuscript, while interesting, presents many preliminary results that need to be properly validated through other methods. The manuscript may not be suitable for publication in EMBO Journal.

Please find below some comments that could improve the quality of the manuscript.

Major comment:
Fig.S2: In their experiment, DNA double-strand breaks are induced by UV-C irradiation. However, the authors should not use UV-C since it induces DNA cross-linking products as major damage. The authors should repeat the experiment using radiation or other sources of damage known to cause double-strand breaks.

Minor comment:
P.20 L472: The authors use the term "over-nanocompaction". However, this term is not defined anywhere; an explanation should be added.

Referee #2:

I am satisfied with the experimental additions and revisions of this manuscript with respect to technical concerns. Overall, I find the data very interesting on the whole. However, I find the paper, overall, highly dissatisfying. While I agree with the authors that the strength of the method is a direct measure of distances between labelled nucleosomes in a living cell context, I think that the discussion needs to be clarified further so that the reader does not overinterpret them. Specifically, the implication is that these results reflect differences in the compaction of consecutive nucleosomes over small scales (e.g., nucleosome 1 interacts with nucleosome 6). The method has not such capacity to resolve this type of interaction from one where the interactions are with very distant sequences and reflect stacking in the a-dimension as opposed to linear (2D) interactions. The only thing that we know about the interactions that are changing is that they are in the low nm range where FRET can occur. If Figure 1A is included in the manuscript, I would like to see it replaced by an illustration that acknowledges that these interactions are not necessarily taking place through the local folding of the 10 nm fiber. Rather, as a massive 3D structure, these interactions could be occurring between distant regions of the polymer such as what is required to form condensates from nucleosomal arrays in vitro.

Referee #3:

The revised manuscript has been very much improved. New data, including the FLIM-FRET assays of differentiated 3T3 cells, single HP1β-depleted mESCs, and ChIP assay for the fluorescent protein-conjugated H2B, has greatly strengthened the paper.

The obtained findings advance our conceptual understanding of heterochromatin and chromatin plasticity in pluripotent cells. I would support this paper for publication in EMBO J. Still, there are a couple of points to be improved before publication:

Major point:
The "nanocompaction" that the authors detected by their FLIM-FRET system is not really clear to me. It seems to me that "nanocompaction" is like "constraining of nucleosomes." Modifying the manuscript a bit more in this line would contribute to readers' better and clearer understanding.

As the authors also mentioned, current single-nucleosome imaging studies have revealed local nucleosome motion (nucleosome fluctuation) in living cells (PMIDs: 35033775 and 35661389), presumably driven by thermal fluctuations (PMID: 35658044). Here, the authors' histone FRAP data only shows chromatin behaviors at larger spatial (~micrometer) and longer temporal (~minutes) scales. This nucleosome fluctuation can facilitate chromatin accessibility, even in condensed regions (PMID: 23246002). I consider that FRET can detect the constraining of nucleosomes. That is, chromatin regions with higher nucleosome fluctuations lower FRET efficiency, even in condensed regions like heterochromatin in ES cells, and lower FRET efficiency does not necessarily mean lower compaction. As the authors discussed, FRET may sense solid-like dense chromatin structures but not liquid-like ones. This is a really intriguing point!

The results from single-nucleosome imaging are well consistent with the authors' FLIM-FRET data. In addition to chemical fixation and 1,6-HD treatments already mentioned in the paper, additional ones are listed:

Mouse embryonic stem (ES) cells have higher nucleosome motion than somatic cells, suggesting greater dynamic properties of chromatin in pluripotent cells (PMIDs: 28712725 and 33440158). With differentiation, nucleosome motion becomes more and more constrained (PMIDs: 28712725 and 33440158).

ATP-depletion rapidly reduces local nucleosome motion in living cells, accompanied by an increase in free Mg^{2+} concentration (PMID: 35658044). Note that ATP-depletion effects do not necessarily mean ATP-dependent active processes (discussed in PMID: 35658044).

TSA treatment increase nucleosome motion (PMID: 28712725).

Transcription inhibitor treatments or rapid depletion of RNA Pol II also increases nucleosome motion (PMID: 30824489).

Minor points
In Figures 1C and 1D, the authors should label which genes or repetitive elements are coded on the euchromatic or heterochromatic regions. Additionally, captions for the light and dense gray bars are missing even in the figure legend.

In Figure S1E, the authors should clarify the measurement of the coefficient of variation (CV) of H2B-GFP in living mESCs, but not in the fixed cells.

Line 237. "the CV as (s/m)" -> "he CV as (s/μ)"

Point-by-point responses to the reviewers' comments

Reviewer #1:

The authors spent a lot of effort to address my concerns and have undertaken several additional experiments. The rigor of their work is commendable.
On the other hand, despite the revised version of the manuscript, it still appeared difficult to discuss and conclude about nanocompaction based on their FRET detection system alone.
We thank the reviewer for agreeing that we added a notable amount of data to address reviewer's concerns. All methods have their own specific technical limitations, in this context we understand the limitation of our FRET detection to measure chromatin compaction at the nanoscale. However, we now clearly define the term "nanocompaction" in the Introduction (page 5, lines 108-110). We also extensively revised the Discussion by adding some text (page 21, lines 512-520) in order that the reader understands the context of the FRET results and that these may in part have been influenced by features other than chromatin structure.

The authors evaluated their FRET detection system in more detail, notably by performing a new experiment with 3T3 cells…..Because the expression level of GFP-H2B and H2B-mCherry is certainly influencing a lot on the intensity of the FRET signal, its expression level should be similar to that of the experiment using ESCs to correlate the results for both cell lines.
We thank the reviewer for this comment. Histone gene expression is tightly controlled to ensure the fine balance between histone abundance and DNA replication for the proper packaging of newly replicated DNA. In this regard, previous work from Kimura and Cook (J Cell Biol. 2001) reported that stable cell lines without alterations in cell cycle regulation were obtained only when tagged histones represented less than 10% of the total histone, similarly as what we observed in our BJ $^{H2B\text{-}2FPs}$ ESCs. Because our 3T3 $^{H2B\text{-}2FPs}$ cell line is stable in culture, and shows similar growth and cell cycle properties as other 3T3 cells that do not express fluorophore-tagged proteins, we believe that the studied 3T3 cells expressed comparable levels of GFP-H2B and H2B-mCherry as ESCs.

-The authors should demonstrate the phenomenon of nanocompaction in heterochromatin using various experimental systems. For example, they could try a proximity ligation assay, to confirm the observations on nanocompaction

As an alternative to measure the nanocompaction of heterochromatin, reviewer #1 suggested the use of the Proximity Ligation Assay (PLA) technology. PLA is broadly used to detect endogenous protein-protein interactions with high sensitivity. However, one of the main limitations of this technique is the requirement to fix and permeabilise the cells. Our present study clearly shows that cell fixation procedures drastically perturb the nanocompaction of chromosomes and chromatin. This insight highlights the interest of using live-cell imaging approaches for exploring the structure of chromatin.

Major comment:

Fig.S2: In their experiment, DNA double-strand breaks are induced by UV-C irradiation. However, the authors should not use UV-C since it induces DNA cross-linking products as major damage. The authors should repeat the experiment using radiation or other sources of damage known to cause double-strand breaks.

It has been shown that double-strand breaks (DSBs) are formed in yeast and mammalian cells following irradiation with 254 nm UV-C, as part of the excision repair of pyrimidine dimers (Bradley and Taylor, 1981, 1983). In the revised version of our manuscript, we added now one of these references (page 12, lines 286-288). In addition, it has been described that γH2Ax spreads across DSBs sites, which makes γH2Ax a reliable marker of DSBs. In addition, γH2Ax retains some effectors, such as 53BP1. Our data (Appendix Figure S2A) clearly show the appearance of a large number of γH2Ax foci and the recruitment of 53BP1 proteins at these sites upon UV-C irradiation. Altogether, our data support the notion that UV-C is one of the sources of double-strand breaks.

Minor comment:

P.20 L472: The authors use the term "over-nanocompaction". However, this term is not defined anywhere; an explanation should be added.

We thank the reviewer for raising this point. The term ‘over-nanocompaction’ was now replaced by adding some explanatory text: “no increased nanocompaction towards higher FRET efficiencies (%) was observed at heterochromatin” (page 20 lines 480-481).

Reviewer #2:

I think that the discussion needs to be clarified further so that the reader does not overinterpret them. Specifically, the implication is that these results reflect differences in the compaction of consecutive nucleosomes over small scales (e.g., nucleosome 1 interacts with nucleosome 6). The method has not such capacity to resolve this type of interaction from one where the interactions are with very distant sequences and reflect stacking in the a-

dimension as opposed to linear (2D) interactions. The only thing that we know about the interactions that are changing is that they are in the low nm range where FRET can occur.
We thank the reviewer for raising this important point. We fully agree with the reviewer's comment and now clarify this point in the Discussion, by adding several lines of text (page 22, lines 512-520; page 25, lines 588-589) in order that the reader does not wrongly interpret our results.

If Figure 1A is included in the manuscript, I would like to see it replaced by an illustration that acknowledges that these interactions are not necessarily taking place through the local folding of the 10 nm fiber. Rather, as a massive 3D structure, these interactions could be occurring between distant regions of the polymer such as what is required to form condensates from nuclesomal arrays in vitro.
We thank the reviewer for this helpful suggestion. We now modified Fig1A to illustrate that our FRET imaging assay is a readout of a combination of intermediate scale interactions as well as distant sequence interactions that could occur within the 3D structural folding of the chromatin polymer.

Reviewer #3:

Major point:
The "nanocompaction" that the authors detected by their FLIM-FRET system is not really clear to me. It seems to me that "nanocompaction" is like "constraining of nucleosomes." Modifying the manuscript a bit more in this line would contribute to readers' better and clearer understanding.
We thank the reviewer for this comment. We now clearly define the term "nanocompaction" in the Introduction (page 5, lines 108-110) and we modify the Discussion by adding some texts (page 22, lines 521-526; page 24 lines 571-573) in order that the reader does not wrongly interpret our results.

As the authors also mentioned, current single-nucleosome imaging studies have revealed local nucleosome motion (nucleosome fluctuation) in living cells (PMIDs: 35033775 and 35661389), presumably driven by thermal fluctuations (PMID: 35658044)… This nucleosome fluctuation can facilitate chromatin accessibility, even in condensed regions (PMID: 23246002). I consider that FRET can detect the constraining of nucleosomes. That is, chromatin regions with higher nucleosome fluctuations lower FRET efficiency, even in

condensed regions like heterochromatin in ES cells, and lower FRET efficiency does not necessarily mean lower compaction.

We thank the reviewer for this interesting discussion point. We now revised our manuscript by adding an entire new paragraph (page 22-23 lines 521-546) in the Discussion, which explores the link between nucleosome fluctuations and nanocompaction changes measured by FRET. In addition, we now cite the five references suggested by the reviewer.

Minor points:

In Figures 1C and 1D, the authors should label which genes or repetitive elements are coded on the euchromatic or heterochromatic regions. Additionally, captions for the light and dense gray bars are missing even in the figure legend.

We apologize for the lack of clarity on these specific points. We now modified Fig1C and 1D following the reviewer's comments by colour-coding the names of the genes according to their chromatin classes. We add further explanatory text in the Figures 1C and 1D legends as well, and added the missing captions in Figures 1C and 1D.

In Figure S1E, the authors should clarify the measurement of the coefficient of variation (CV) of H2B-GFP in living mESCs, but not in the fixed cells.

We thank the reviewer for pointing this out to us. We have clarified this point in the new version of the Appendix Figure S1E by adding the labelling "Living mESCs" on the top of the panels.

Line 237. "the CV as (s/m)" -> "he CV as (s/µ)"

We apologize for this typo and corrected it in the new version of the manuscript (page 11, line 242).

Dr. David Llères
Institute of Molecular Genetics of Montpellier (IGMM), CNRS, University of Montpellier
IGMM
1919 Route de Mende
Montpellier 34293
France

15th Mar 2023

Re: EMBOJ-2021-110286R1
Evidence for Low Nanocompaction of Heterochromatin in Living Embryonic Stem Cells

Dear Dr. Llères,

Thank you for your patience during our assessment of your re-revised manuscript. The study has now been checked by myself as well as by our data editors, and I am happy to say is now ready to be accepted in principle. Before being able to send a formal letter of acceptance, there are only the following editorial points that still need to be addressed:

- The pre-acceptance checks by our data editors have raised several queries with the data descriptors in the figure legends, which you will find as comments in the attached edited/commented Word document with activated "Track changes" option. I would appreciate if you incorporated the requested final text modifications and answered the Figure legend queries directly in this version (and modified figures where necessary), uploading the edited main text document upon resubmission with changes/additions STILL HIGHLIGHTED via the "TRACK CHANGES" option, to facilitate our final checking.

- On the abstract page of the manuscript, please include 4-5 general keyword terms to enhance searchability.

- As we are switching from a free-text author contribution statement towards a more formal statement based on Contributor Role Taxonomy (CRediT) terms, please remove the present Author Contribution section and instead specify each author's contribution(s) directly in the Author Information page of our submission system during upload of the final manuscript. See https://casrai.org/credit/ for more information.

- Please adjust the header and the format of the Disclosure and competing interests statement (next to the Acknowledgment section) as specified in our Guide to Authors - for details, see https://www.embopress.org/competing-interests

- For the three tables included in the manuscript text: If they should remain part of the article proper, please rename them into "Table 1/2/3" both in their headers and when referencing them in the text. (If you would prefer to have them as Expanded View Tables, each of them should be uploaded as a separate file, but they would in this case not be typeset and not be directly linked in the HTML version.)

- Image data: We noticed that in Fig EV2A, the upper middle panel does not exhibit any background signal or noise whatsoever. I realize that in the absence of mCherry-H2B expression there should of course be no signal here, but please nevertheless please double-check that you have inserted a proper micrograph of the same cells/field, taken under the same settings as the one in the lower panel, rather than a blank "placeholder" panel here. Providing us with raw data files for this image would be helpful.

- Finally, please provide suggestions for a short 'blurb' text prefacing and summing up the conceptual aspect of the study in two sentences (max. 250 characters), followed by 3-5 one-sentence 'bullet points' with brief factual statements of key results of the paper; they will form the basis of an editor-written 'Synopsis' accompanying the online version of the article. Please also upload a synopsis image, which can be used as a "visual title" for the synopsis section of your paper. The image should be in PNG or JPG format, and please make sure that it remains in the modest dimensions of (exactly) 550 pixels wide and 300-600 pixels high.

I am therefore returning the manuscript to you once more for a final round of minor revision (as this is the simplest way by which you might upload not just modified text but also modified figures if necessary). Once we will have received them, we should be ready to swiftly proceed with formal acceptance and production of the manuscript!

Yours sincerely,

Hartmut Vodermaier

Hartmut Vodermaier, PhD
Senior Editor, The EMBO Journal
h.vodermaier@embojournal.org

*** PLEASE NOTE: All revised manuscript are subject to initial checks for completeness and adherence to our formatting guidelines. Revisions may be returned to the authors and delayed in their editorial re-evaluation if they fail to comply to the following requirements (see also our Guide to Authors for further information):

1) Every manuscript requires a Data Availability section (even if only stating that no deposited datasets are included). Primary datasets or computer code produced in the current study have to be deposited in appropriate public repositories prior to resubmission, and reviewer access details provided in case that public access is not yet allowed. Further information: embopress.org/page/journal/14602075/authorguide#dataavailability

2) Each figure legend must specify
- size of the scale bars that are mandatory for all micrograph panels
- the statistical test used to generate error bars and P-values
- the type error bars (e.g., S.E.M., S.D.)
- the number (n) and nature (biological or technical replicate) of independent experiments underlying each data point
- Figures may not include error bars for experiments with n<3; scatter plots showing individual data points should be used instead.

3) Revised manuscript text (including main tables, and figure legends for main and EV figures) has to be submitted as editable text file (e.g., .docx format). We encourage highlighting of changes (e.g., via text color) for the referees' reference.

4) Each main and each Expanded View (EV) figure should be uploaded as individual production-quality files (preferably in .eps, .tif, .jpg formats). For suggestions on figure preparation/layout, please refer to our Figure Preparation Guidelines: http://bit.ly/EMBOPressFigurePreparationGuideline

5) Point-by-point response letters should include the original referee comments in full together with your detailed responses to them (and to specific editor requests if applicable), and also be uploaded as editable (e.g., .docx) text files.

6) Please complete our Author Checklist, and make sure that information entered into the checklist is also reflected in the manuscript; the checklist will be available to readers as part of the Review Process File. A download link is found at the top of our Guide to Authors: embopress.org/page/journal/14602075/authorguide

7) All authors listed as (co-)corresponding need to deposit, in their respective author profiles in our submission system, a unique ORCiD identifier linked to their name. Please see our Guide to Authors for detailed instructions.

8) Please note that supplementary information at EMBO Press has been superseded by the 'Expanded View' for inclusion of additional figures, tables, movies or datasets; with up to five EV Figures being typeset and directly accessible in the HTML version of the article. For details and guidance, please refer to: embopress.org/page/journal/14602075/authorguide#expandedview

9) Digital image enhancement is acceptable practice, as long as it accurately represents the original data and conforms to community standards. If a figure has been subjected to significant electronic manipulation, this must be clearly noted in the figure legend and/or the 'Materials and Methods' section. The editors reserve the right to request original versions of figures and the original images that were used to assemble the figure. Finally, we generally encourage uploading of numerical as well as gel/blot image source data; for details see: embopress.org/page/journal/14602075/authorguide#sourcedata

At EMBO Press, we ask authors to provide source data for the main manuscript figures. Our source data coordinator will contact you to discuss which figure panels we would need source data for and will also provide you with helpful tips on how to upload and organize the files.

Further information is available in our Guide For Authors: https://www.embopress.org/page/journal/14602075/authorguide

In the interest of ensuring the conceptual advance provided by the work, we recommend submitting a revision within 3 months (13th Jun 2023). Please discuss the revision progress ahead of this time with the editor if you require more time to complete the revisions. Use the link below to submit your revision:

Link Not Available

Dr. David Llères
Institute of Molecular Genetics of Montpellier (IGMM), CNRS, University of Montpellier
IGMM
1919 Route de Mende
Montpellier 34293
France

29th Mar 2023

Re: EMBOJ-2021-110286R2
Evidence for Low Nanocompaction of Heterochromatin in Living Embryonic Stem Cells

Dear Dr. Llères,

Thank you for submitting your final revised manuscript for our consideration. I am pleased to inform you that we have now accepted it for publication in The EMBO Journal.

Your article will be processed for publication in The EMBO Journal by EMBO Press and Wiley, who will contact you with further information regarding production/publication procedures and license requirements. You will also be provided with page proofs after copy-editing and typesetting of main manuscript and expanded view figure files.

Should you be planning a Press Release on your article, please get in contact with embojournal@wiley.com as early as possible, in order to coordinate publication and release dates.

Thank you again for this contribution to The EMBO Journal and congratulations on a successful publication! Please consider us again in the future for your most exciting work.

Yours sincerely,

Hartmut Vodermaier

Hartmut Vodermaier, PhD
Senior Editor, The EMBO Journal
h.vodermaier@embojournal.org

Please note that it is EMBO Journal policy for the transcript of the editorial process (containing referee reports and your response letter) to be published as an online supplement to each paper. If you do NOT want this, you will need to inform the Editorial Office via email immediately. More information is available here:
https://www.embopress.org/page/journal/14602075/authorguide#transparentprocess

EMBO Press Author Checklist

Corresponding Author Name: LLERES David , FEIL Robert
Journal Submitted to: The EMBO Journal
Manuscript Number: EMBOJ-2021-110286R-Q

USEFUL LINKS FOR COMPLETING THIS FORM
The EMBO Journal - Author Guidelines
EMBO Reports - Author Guidelines
Molecular Systems Biology - Author Guidelines
EMBO Molecular Medicine - Author Guidelines

Reporting Checklist for Life Science Articles (updated January
This checklist is adapted from Materials Design Analysis Reporting (MDAR) Checklist for Authors. MDAR establishes a minimum set of requirements in transparent reporting in the life sciences (see Statement of Task: 10.31222/osf.io/9sm4x). Please follow the journal's guidelines in preparing your
Please note that a copy of this checklist will be published alongside your article.

Abridged guidelines for figures
1. Data
The data shown in figures should satisfy the following conditions:
- the data were obtained and processed according to the field's best practice and are presented to reflect the results of the experiments in an accurate and unbiased manner.
- ideally, figure panels should include only measurements that are directly comparable to each other and obtained with the same assay.
- plots include clearly labeled error bars for independent experiments and sample sizes. Unless justified, error bars should not be shown for technical
- if n<5, the individual data points from each experiment should be plotted. Any statistical test employed should be justified.
- Source Data should be included to report the data underlying figures according to the guidelines set out in the authorship guidelines on Data

2. Captions
Each figure caption should contain the following information, for each panel where they are relevant:
- a specification of the experimental system investigated (eg cell line, species name).
- the assay(s) and method(s) used to carry out the reported observations and measurements.
- an explicit mention of the biological and chemical entity(ies) that are being measured.
- an explicit mention of the biological and chemical entity(ies) that are altered/varied/perturbed in a controlled manner.
- the exact sample size (n) for each experimental group/condition, given as a number, not a range;
- a description of the sample collection allowing the reader to understand whether the samples represent technical or biological replicates (including how many animals, litters, cultures, etc.).
- a statement of how many times the experiment shown was independently replicated in the laboratory.
- definitions of statistical methods and measures:
  - common tests, such as t-test (please specify whether paired vs. unpaired), simple χ^2 tests, Wilcoxon and Mann-Whitney tests, can be unambiguously identified by name only, but more complex techniques should be described in the methods section;
  - are tests one-sided or two-sided?
  - are there adjustments for multiple comparisons?
  - exact statistical test results, e.g., P values = x but not P values < x;
  - definition of 'center values' as median or average;
  - definition of error bars as s.d. or s.e.m.

Please complete ALL of the questions below.
Select "Not Applicable" only when the requested information is not relevant for your study.

Materials

Newly Created Materials	Information included in the manuscript?	In which section is the information available? (Reagents and Tools Table, Materials and Methods, Figures, Data Availability Section)
New materials and reagents need to be available; do any restrictions apply?	Yes	Materials and Methods

Antibodies	Information included in the manuscript?	In which section is the information available? (Reagents and Tools Table, Materials and Methods, Figures, Data Availability Section)
For **antibodies** provide the following information:<br>- Commercial antibodies: RRID (if possible) or supplier name, catalogue number and or/clone number<br>- Non-commercial: RRID or citation	Yes	Materials and Methods

DNA and RNA sequences	Information included in the manuscript?	In which section is the information available? (Reagents and Tools Table, Materials and Methods, Figures, Data Availability Section)
**Short novel DNA or RNA including primers, probes**: provide the sequences.	Yes	Materials and Methods, Table EV1,Table EV2

Cell materials	Information included in the manuscript?	In which section is the information available? (Reagents and Tools Table, Materials and Methods, Figures, Data Availability Section)
**Cell lines:** Provide species information, strain. Provide accession number in repository **OR** supplier name, catalog number, clone number, and/**OR** RRID.	Yes	Materials and Methods
**Primary cultures:** Provide species, strain, sex of origin, genetic modification status.	Yes	Materials and Methods
Report if the cell lines were recently **authenticated** (e.g., by STR profiling) and tested for mycoplasma contamination.	Yes	Materials and Methods

Experimental animals	Information included in the manuscript?	In which section is the information available? (Reagents and Tools Table, Materials and Methods, Figures, Data Availability Section)
**Laboratory animals or Model organisms:** Provide species, strain, sex, age, genetic modification status. Provide accession number in repository **OR** supplier name, catalog number, clone number, **OR** RRID.	Not Applicable	
**Animal observed in or captured from the field:** Provide species, sex, and age where possible.	Not Applicable	
Please detail **housing and husbandry conditions**.	Not Applicable	

Plants and microbes	Information included in the manuscript?	In which section is the information available? (Reagents and Tools Table, Materials and Methods, Figures, Data Availability Section)
**Plants:** provide species and strain, ecotype and cultivar where relevant, unique accession number if available, and source (including location for collected wild specimens).	Not Applicable	
**Microbes:** provide species and strain, unique accession number if available, and source.	Not Applicable	

Human research participants	Information included in the manuscript?	In which section is the information available? (Reagents and Tools Table, Materials and Methods, Figures, Data Availability Section)
If collected and within the bounds of privacy constraints report on age, sex and gender or ethnicity for all study participants.	Not Applicable	

Core facilities	Information included in the manuscript?	In which section is the information available? (Reagents and Tools Table, Materials and Methods, Figures, Data Availability Section)
If your work benefited from core facilities, was their service mentioned in the acknowledgments section?	Yes	Acknowledgements section

Design

Study protocol	Information included in the manuscript?	In which section is the information available? (Reagents and Tools Table, Materials and Methods, Figures, Data Availability Section)
If study protocol has been **pre-registered, provide DOI in the manuscript**. For clinical trials, provide the trial registration number **OR** cite DOI.	Not Applicable	
Report the **clinical trial registration number** (at ClinicalTrials.gov or equivalent), where applicable.	Not Applicable	

Laboratory protocol	Information included in the manuscript?	In which section is the information available? (Reagents and Tools Table, Materials and Methods, Figures, Data Availability Section)
Provide DOI OR other citation details if **external detailed step-by-step protocols** are available.	Not Applicable	

Experimental study design and statistics	Information included in the manuscript?	In which section is the information available? (Reagents and Tools Table, Materials and Methods, Figures, Data Availability Section)
Include a statement about **sample size** estimate even if no statistical methods were used.	Not Applicable	
Were any steps taken to minimize the effects of subjective bias when allocating animals/samples to treatment (e.g. **randomization procedure**)? If yes, have they been described?	Not Applicable	
Include a statement about **blinding** even if no blinding was done.	Not Applicable	
Describe **inclusion/exclusion criteria** if samples or animals were excluded from the analysis. Were the criteria pre-established?<br><br>If sample or data points were omitted from analysis, report if this was due to attrition or intentional exclusion and provide justification.	Not Applicable	
For every figure, are **statistical tests** justified as appropriate? Do the data meet the assumptions of the tests (e.g., normal distribution)? Describe any methods used to assess it. Is there an estimate of variation within each group of data? Is the variance similar between the groups that are being statistically compared?	Yes	Meterials and Methods (Statistical Analysis section); Figures and Figure legends

Sample definition and in-laboratory replication	Information included in the manuscript?	In which section is the information available? (Reagents and Tools Table, Materials and Methods, Figures, Data Availability Section)
In the figure legends: state number of times the experiment was **replicated** in laboratory.	Yes	Figure legends
In the figure legends: define whether data describe **technical or biological replicates**.	Yes	Figure legends

Ethics

Ethics	Information included in the manuscript?	In which section is the information available? (Reagents and Tools Table, Materials and Methods, Figures, Data Availability Section)
Studies involving **human participants**: State details of **authority granting ethics approval** (IRB or equivalent committee(s), provide reference number for approval.	Not Applicable	
Studies involving **human participants**: Include a statement confirming that **informed consent** was obtained from all subjects and that the experiments conformed to the principles set out in the WMA Declaration of Helsinki and the Department of Health and Human Services Belmont Report.	Not Applicable	
Studies involving **human participants:** For publication of **patient photos**, include a statement confirming that consent to publish was obtained.	Not Applicable	
Studies involving experimental **animals**: State details of **authority granting ethics approval** (IRB or equivalent committee(s), provide reference number for approval. Include a statement of compliance with ethical regulations.	Not Applicable	
Studies involving **specimen and field samples:** State if relevant **permits** obtained, provide details of authority approving study; if none were required, explain why.	Not Applicable	

Dual Use Research of Concern (DURC)	Information included in the manuscript?	In which section is the information available? (Reagents and Tools Table, Materials and Methods, Figures, Data Availability Section)
Could your study fall under dual use research restrictions? Please check biosecurity documents and list of **select agents and toxins** (CDC): https://www.selectagents.gov/sat/list.htm	Not Applicable	
If you used a select agent, is the security level of the lab appropriate and reported in the manuscript?	Not Applicable	
If a study is subject to dual use research of concern regulations, is the name of the **authority granting approval and reference number** for the regulatory approval provided in the manuscript?	Not Applicable	

Reporting

The MDAR framework recommends adoption of discipline-specific guidelines, established and endorsed through community initiatives. Journals have their own policy about requiring specific guidelines and recommendations to complement MDAR.

Adherence to community standards	Information included in the manuscript?	In which section is the information available? (Reagents and Tools Table, Materials and Methods, Figures, Data Availability Section)
State if relevant guidelines or checklists (e.g., **ICMJE, MIBBI, ARRIVE, PRISMA**) have been followed or provided.	Not Applicable	
For **tumor marker prognostic studies,** we recommend that you follow the **REMARK** reporting guidelines (see link list at top right). See author guidelines, under 'Reporting Guidelines'. Please confirm you have followed these guidelines.	Not Applicable	
For **phase II and III randomized controlled trials**, please refer to the **CONSORT** flow diagram (see link list at top right) and submit the CONSORT checklist (see link list at top right) with your submission. See author guidelines, under 'Reporting Guidelines'. Please confirm you have submitted this list.	Not Applicable	

Data Availability

Data availability	Information included in the manuscript?	In which section is the information available? (Reagents and Tools Table, Materials and Methods, Figures, Data Availability Section)
Have **primary datasets** been deposited according to the journal's guidelines (see 'Data Deposition' section) and the respective accession numbers provided in the Data Availability Section?	Not Applicable	
Were **human clinical and genomic datasets** deposited in a public access-controlled repository in accordance to ethical obligations to the patients and to the applicable consent agreement?	Not Applicable	
Are **computational models** that are central and integral to a study available without restrictions in a machine-readable form? Were the relevant accession numbers or links provided?	Not Applicable	
If publicly available data were reused, provide the respective **data citations in the reference list**.	Not Applicable	
